# Nanoscale Patterning of Carbon Nanotubes: Techniques, Applications, and Future

**DOI:** 10.1002/advs.202001778

**Published:** 2020-11-23

**Authors:** Alexander Corletto, Joseph G. Shapter

**Affiliations:** ^1^ Australian Institute for Bioengineering and Nanotechnology The University of Queensland Brisbane Queensland 4072 Australia

**Keywords:** carbon nanotube devices, carbon nanotubes, device development, dielectrophoresis, field effect transistors, nanoscale patterning, nanotube dispersions

## Abstract

Carbon nanotube (CNT) devices and electronics are achieving maturity and directly competing or surpassing devices that use conventional materials. CNTs have demonstrated ballistic conduction, minimal scaling effects, high current capacity, low power requirements, and excellent optical/photonic properties; making them the ideal candidate for a new material to replace conventional materials in next‐generation electronic and photonic systems. CNTs also demonstrate high stability and flexibility, allowing them to be used in flexible, printable, and/or biocompatible electronics. However, a major challenge to fully commercialize these devices is the scalable placement of CNTs into desired micro/nanopatterns and architectures to translate the superior properties of CNTs into macroscale devices. Precise and high throughput patterning becomes increasingly difficult at nanoscale resolution, but it is essential to fully realize the benefits of CNTs. The relatively long, high aspect ratio structures of CNTs must be preserved to maintain their functionalities, consequently making them more difficult to pattern than conventional materials like metals and polymers. This review comprehensively explores the recent development of innovative CNT patterning techniques with nanoscale lateral resolution. Each technique is critically analyzed and applications for the nanoscale‐resolution approaches are demonstrated. Promising techniques and the challenges ahead for future devices and applications are discussed.

## Graphical abstract

Carbon nanotube (CNT) devices are achieving maturity and directly competing with devices using conventional materials. However, a major challenge to fully commercialize is the scalable placement of CNTs into nanoscale patterns/architectures into macroscale devices. This review comprehensively explores the recent development of nanoscale resolution CNT patterning techniques. Each technique is critically analyzed, applications are demonstrated, and challenges/opportunities are discussed.

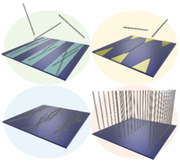

## 1 Introduction

In the past few decades, carbon nanotubes (CNTs) have attracted a lot of research interest due to their excellent electrical and physical properties. To exploit these properties in new technologies, it is critical to control the placement of CNTs into desired patterns and architectures within devices. However, CNTs have relatively large, high aspect ratio structures that must be preserved to maintain their functionalities. Patterning them is consequently more difficult than traditional materials like metals and polymers that act as continuous bulk solids or liquid phases. The challenge is to use innovative patterning techniques that can preserve the structure of the nanomaterials, while maintaining desirable patterning characteristics (high resolution, low cost, low temperature, high throughput, etc.). CNTs have been patterned using a variety of techniques since their discovery, and each approach has particular advantages and disadvantages. Some patterning techniques have high throughput and are inexpensive, but often have poor lateral resolution and electrical properties (ink‐jet printing, gravure printing, screen printing, microcontact printing).^[^ ^1^ ^]^ Other techniques have high lateral resolution, but can be expensive, have slow throughput or require modified substrates or printing conditions that limit their utility.^[^ ^2^ ^]^ There is often a trade‐off in desirable properties. The aim of researchers is to find CNT patterning techniques that can simultaneously utilize CNTs’ superior properties in devices, while being practical for mass production.

There is an increasing demand to develop new viable manufacturing techniques that can pattern CNTs in devices at the nanoscale while retaining excellent properties. Semiconductor manufacturers are dealing with increasing issues with scaling down components for the next technology nodes and are actively exploring alternative materials and lithographic/patterning methods. CNTs are a leading candidate for replacing silicon in transistors and copper in interconnects.^[^ ^3^ ^]^ Recently, an advanced 16‐bit all‐CNT transistor computer was reported,^[^ ^4^ ^]^ demonstrating the current maturity of CNTs as an integrated circuit material. However, one of the current bottlenecks in using them is the controlled patterning in devices down to nanoscale resolution. Controlled placement of individual CNTs is also critical for photonic applications to ensure high‐quality emission and absorption down to the single photon limit. Flexible, biocompatible, and cheap electronics are now the subject of intense research with CNTs being one of the leading materials of choice for these devices. The ability to pattern CNTs at nanoscale resolution on flexible substrates at high throughput will be critical for fabricating an array of new accessible sensors and electrical devices. Cheaper and prolific high‐performance electronics are critical to certain nascent, emerging technologies, as they will provide more data inputs to realize their full potential. Technologies and innovations that benefit from greater amounts of input data include big data, machine learning, AI, and Internet‐of‐Things (IoT).^[^ ^5^ ^]^ Other important applications for CNT electronics include thin film transistors, transparent conducting films, wearable electronics, human–machine interfaces, healthcare, and flexible, and large area displays.^[^ ^1^, ^6^ ^]^ Patterning CNTs at the nanoscale is important for these technologies in order to boost the speed and power densities, shrink their total size for different applications and portability, decrease cost and use of resources, and decrease energy usage.

**Figure 1:**
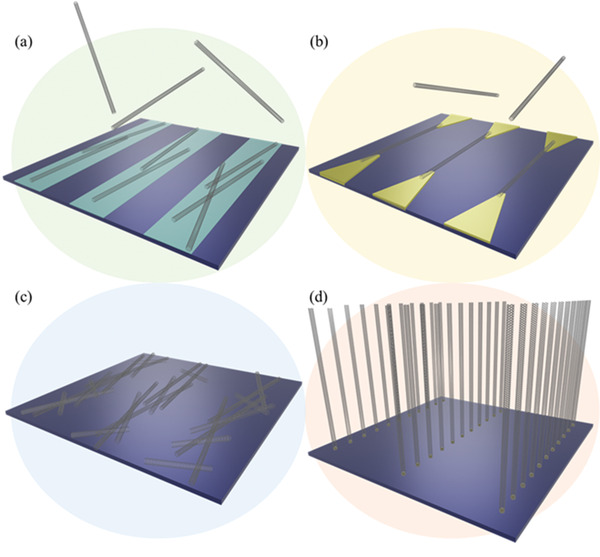
Examples of current nanoscale CNT patterning techniques including: a) self‐assembly of CNTs from dispersions on self‐assembled monolayer (SAM) patterned substrates, b) DEP patterning of CNTs from dispersions, c) oxidation etching of CNT networks through patterned resist, and d) prepatterning of CNT growth catalysts.

Therefore, a review of the research in the field over the past few decades and future challenges is critical. This review comprehensively explores and analyzes nanoscale lateral resolution CNT patterning techniques that have been explored up to this point, including self‐assembly of CNTs from dispersions on self‐assembled monolayer (SAM) patterned substrates (**Figure** **1**a), dielectrophoresis (DEP) patterning of CNTs from dispersions (Figure 1b), oxidation etching of CNT networks through patterned resist (Figure 1c), prepatterning of CNT growth catalysts (Figure 1d), and more. The physical and chemical concepts of CNTs that enable or limit the patterning techniques and current applications will also be discussed. Current challenges and future directions will be discussed to further develop this research area to discover improved patterning techniques that will be required for future technologies.

### 1.1 CNT Properties

**Figure 2:**
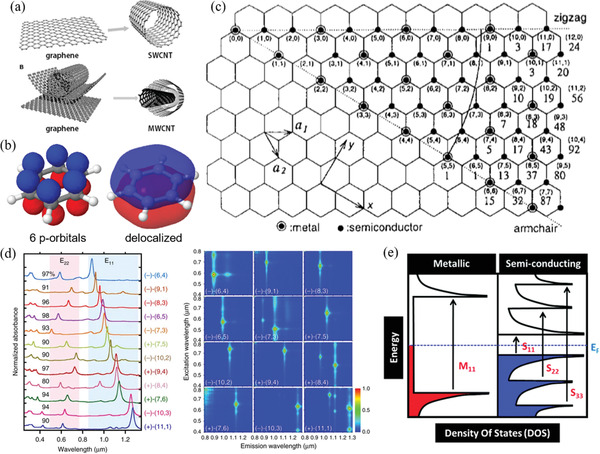
a) CNTs are essentially tubes of rolled‐up graphene. SWCNTs consist of one wall of rolled‐up graphene and MWCNTs have multiple walls. Reproduced under the terms of the CC BY 3.0.^[^ ^20^ ^]^ Copyright 2014, The Authors, published by Frontiers. b) The delocalized p‐orbitals of a benzene ring form a resonance‐stabilizing *π*‐electron system. This same structure occurs in CNTs along the whole lattice. c) Chiral indices of CNTs represented graphically on the lattice. The basis vectors **a_1_** and **a_2_** are presented for reference. Each “step” of **a_1_** and **a_2_** is represented in *n* or *m*, respectively. The chirality of a CNT is determined by the number of exact *n* and *m* steps to circumnavigate the CNT. Reproduced with permission.^[^ ^12a^ ^]^ Copyright 1995, Elsevier. d) Optical absorption spectra and photoluminescence maps of various CNT chiralities. Reproduced under CC BY 4.0.^[^ ^21^ ^]^ Copyright 2016, The Authors, published by Springer Nature. e) The typical density of states (DOS) of metallic and semiconducting SWCNTs. The arrows represent electronic transitions between the van Hove singularities that appear in the optical spectra. M_11_ and S_11_ are the lowest energy electronic transitions for metallic and semiconducting SWCNTs, respectively. Note the gap in DOS present in the semiconducting SWCNTs. Reproduced with permission.^[^ ^22^ ^]^ Copyright 2012, The Royal Society of Chemistry.

CNTs are large macromolecules that consist of many sp^2^‐hybridized carbon atoms connected in a planar hexagonal lattice in a curled, tube morphology.^[^ ^7^ ^]^ It is effectively a tubular graphene sheet (**Figure** **2**a). CNTs can also be multiple nanotubes sitting inside each other, with each nanotube layer called a “wall.” They are named by the number of walls the CNT contains, including single‐walled carbon nanotubes (SWCNTs) (1 wall), double‐walled carbon nanotubes (DWCNTs) (2 walls), or multi‐walled carbon nanotubes (MWCNTs) (>2 walls). CNTs can have a very high aspect ratio, with SWCNTs having typical diameters of 0.8–2.0 nm, MWCNTs typical diameters of 5–20 nm, and both having lengths up to centimeters long.^[^ ^8^ ^]^ This high aspect ratio can allow for very high conduction over a long distance in devices. CNTs as a large lattice of sp^2^ carbon atoms have delocalized p‐orbitals above the lattice (Figure 2b). This forms a resonance stabilizing *π*‐electron system, similar to aromatic molecules like benzene, except extending along the whole length of the CNT. This delocalized electron system provides the highly conducting path along the CNTs. CNTs are highly conductive along individual nanotubes with ballistic conductance achievable for distances under their extraordinary mean free path at room temperature of ≈1 µm. Conductance for longer CNTs is also very high at ≈2 *e* ^2^/*h* compared to ballistic conductance of ≈4 *e* ^2^/*h*, which allows CNTs to surpass conventional conductors like Cu and Ag. They also have very high current densities of up to ≈4 × 10^9^ A cm^−2^, which is three orders of magnitude higher than Cu before breakdown,^[^ ^9^ ^]^ and high thermal conductivity of up to 3500 W m^−1^ K^−1^ (room temperature SWCNTs).^[^ ^10^ ^]^ However, there is a high electric potential barrier of 0.1–10 MΩ between adjacent CNTs (intertube junction).^[^ ^11^ ^]^ This results in a higher resistance along CNT films or networks that require charge carriers to transport through intertube junctions, so these networks can have lower conductance than similar geometry Cu/Ag.

Each CNT wall comes in a variety of chiralities (Figure 2c).^[^ ^12^ ^]^ The CNT chirality is determined by the chiral vector expressed as **C_h_** = *n* **a_1_** + *m* **a_2_**, and are most often represented by the two integers (*n,m*) that constitute the chiral index. *n* and *m* represent the number of basis vector “steps” (**a_1_** and **a_2_**) along diagonal adjacent carbon atoms in the lattice that circumnavigates the CNT wall. The chiral angle is the angle of the chiral vector compared to the default “zig‐zag” conformation chiral vector (*n*,*0*). Different CNT chiralities can have very different electronic properties due to the varying electronic band structure from the different twisted arrangements of the lattice. Consequently, SWCNTs can come in a variety of different bandgaps, producing a spectrum of electronic varieties from metallic to semiconducting. Standard SWCNT synthesis methods produce a “natural” distribution of SWCNT chiralities, with ≈1/3 metallic and 2/3 semiconducting with a very wide range of different bandgaps.^[^ ^13^ ^]^ Electronic devices require only semiconducting or only metallic materials for different functions, so a heterogenous mix of SWCNT chiralities will generally have inferior properties. Optical and photonic devices generally require even more specificity to single chiralities to control absorbance and emission spectra. Sorting the heterogenous mix into specific chiralities or synthesizing chirally homogenous SWCNTs is therefore imperative and much research has investigated these objectives.^[^ ^14^ ^]^ MWCNTs on the other hand have multiple walls with different chiralities, which tends to average out the properties and result in almost exclusively metallic MWCNTs. DWCNTs have more complex electronic and optical properties from complex wall interactions that still express specific properties.^[^ ^15^ ^]^ CNTs express 1D‐like properties in their allowed electron momentum states due to the tight confinement of electrons with their nanometer diameter dimensions.^[^ ^12^ ^]^ This results in CNTs, especially SWCNTs, expressing van Hove singularities in their electronic density of states; large sharp peaks of electron state densities at particular energy levels and at the band gap edge (Figure 2d). Allowed electronic energy transitions between the singularities (typically metallic M_11_ transitions and semiconducting S_11_, S_22_, and S_33_ transitions) provide strong characteristic peaks in optical and photoluminescence spectra that are useful for many optical and photonic applications (Figure 2e).^[^ ^16^ ^]^ Remember that a range of chiralities can be selected to tune the band gaps and thus tune the optical spectra in a wide range.

CNTs therefore have some significant advantages over conventional materials. CNTs can have very high conductance and current density, which is useful for high power electronics and extreme scaled devices. CNTs are inherently flexible and are quite stable in a variety of conditions, giving them an advantage for use in more rugged, extreme, or complex environments like the human body, farms, manufacturing, transport, robotics, etc. The tunable electronic properties of CNTs allow them to take on a variety of different roles within devices and be tailored to the specific application. CNTs can also experience ballistic transport at shorter lengths, providing very low resistance conduction, reducing heat production in the device, and lowering operating voltages for low‐power electronics.^[^ ^17^ ^]^ CNTs can be made from natural carbon sources, using minimal or no mined material for their production. Electronics, photonics, computers, and sensors produced using nonmined materials help to prevent the environmental impacts of mining. Certain metals used for electronic devices are also rare or have certain limited worldwide reserves. Using nonmined materials for electronics avoids the issue of running low or depleting the materials for electronics, especially with an exponentially increasing demand for these devices in the future. Recent research even demonstrated that CO_2_ can be used to electrochemically synthesize CNTs, allowing a greenhouse gas to be reduced in the atmosphere while simultaneously producing a valuable material product for electronics.^[^ ^18^ ^]^

### 1.2 Importance of Nanoscale Patterning of CNTs

While alignment and patterning of CNTs both require control of CNTs at small scales, patterning of CNTs requires controlling the specific location of CNTs on substrates. CNT alignment simply involves the orientation of the CNTs relative to each other. Actual patterning of the CNTs and control of their specific locations on substrates are required to produce functional components in devices like transistor channels, electrodes, wiring, interconnects, etc. Conversely, although alignment can improve the quality of devices, many devices do not require aligned CNTs to be functional, as random CNT networks are used for fabricating many functional devices. CNT alignment and patterning can use similar forces for control, but there are fundamentally different techniques used to control their precise position on substrates rather than just orientation. It is also important to note that many of the nanoscale patterning techniques presented in this review for CNTs can also be used for patterning other 1D materials that have been discovered and fabricated recently, including different metal, metal alloy, or semiconducting 1D nanowires.^[^ ^19^ ^]^

### 1.3 Figures of Merit for CNT Patterning

Particular properties of the CNT patterning techniques and the resulting CNT patterns are important for enhancing their effectiveness for different applications. These properties can be considered figures of merit for the patterning technique, and different figures of merit will be important for different applications. “Coverage” is the proportion of the patterned substrate area that is completely covered in deposited/adhered CNTs. Many applications will clearly aim to achieve 100% coverage to ensure uniform properties, however some applications do not need or should not have full coverage. Sometimes, one or a few CNTs deposited in each patterned region of a substrate is all that is required for the application. “Yield” or “efficiency” will vary for each application, but it is the proportion of devices or substrate area that is correctly patterned with CNTs. For example, some carbon nanotube field effect transistors (CNTFETs) require at least one SWCNT to be deposited across an electrode pair to function, and so the yield or efficiency is the proportion of total electrode pairs that have at least one SWCNT deposited. “Density” or “CNT density” is the countable number of nanotubes patterned per substrate area. A substrate area of 1 µm^2^ that has 10 individual CNTs deposited in it has a CNT density of 10 µm^−2^.

## 2 CNT Processability and Physical/Chemical Impacts on Patterning

While CNTs have remarkable properties that make them a leading contender to become the critical material for creating next‐generation devices, the synthesis and processing of CNTs for their use in manufacturing is not trivial. CNTs have unique physical and chemical properties that have created difficulties for researchers and manufacturers to process them for use in devices. Understanding the chemistry and physics of CNTs and their interaction with the environment is critical to determine the limitations in current patterning techniques and identify potential paths to circumvent these limitations to advance the field.

### 2.1 Dispersion of CNTs

**Figure 3:**
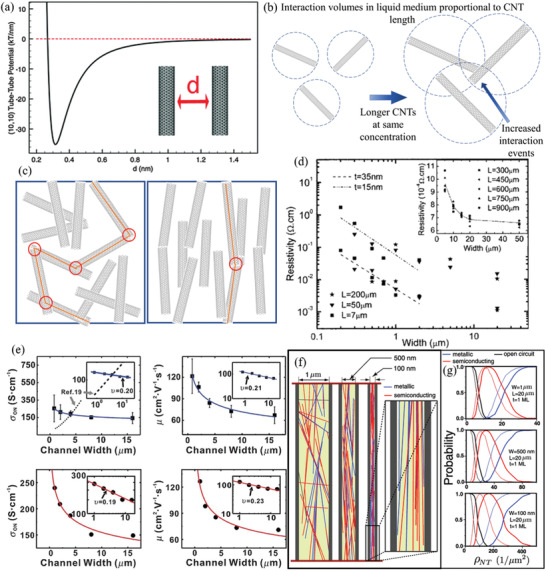
a) Example tube–tube interaction potential for two (10,10) CNTs. Reproduced with permission.^[^ ^25^ ^]^ Copyright 2012, The Royal Society of Chemistry. b) Blue dotted circles represent interaction volumes of CNTs in dispersions. When longer CNTs are present at the same concentration, their interaction volumes are more likely to overlap, causing more interactions per unit time and consequently faster aggregation. c) Random CNT networks tend to have increased resistance due to their meandering percolation pathways (orange lines) having more intertube junctions (red circles) than aligned CNT networks. d) Plot of resistivity against line width for lines of randomly oriented CNT networks patterned via oxidation etching. *L* is line length and the best fit lines plotted are for line thicknesses of 35 and 15 nm. Scaling is resistivity ∝ *W* ^−1.53^ for *t* = 15 nm and ∝*W* ^−1.43^ for *t* = 35 nm. The inverse relationship is similar to conventional conductive materials. Reproduced with permission.^[^ ^28^ ^]^ Copyright 2006, AIP Publishing. e) Plots of conductivity (left) and mobility (right) against channel width for self‐aligning lines of sub‐monolayer CNT networks patterned via CNT dispersion on patterned SAM substrate. Experimental results (blue) and simulations (red) are compared and insets are log–log plots showing scaling behavior. Note that conductivity/mobility increases with decreasing line width due to self‐alignment, in contrast to conventional conductive material. Reproduced with permission.^[^ ^29^ ^]^ Copyright 2009, Wiley‐VCH. f) 2D simulation of a natural distribution of metallic (blue) and semiconducting (red) SWCNTs randomly distributed in confined 20 µm channels (5 µm length shown). Note the increasing alignment with decreasing width. g) Plots of the probability of metallic or semiconducting behavior of the lines against CNT density. Note the increasing CNT density required to achieve semiconducting behavior as width decreases. Reproduced with permission.^[^ ^30^ ^]^ Copyright 2010, American Chemical Society.

The main method to manipulate CNTs in bulk is using dispersions of CNTs in liquids.^[^ ^23^ ^]^ However, to effectively and stably disperse CNTs, the strong attractive van der Waals forces between contacting CNTs must be overcome to separate CNT bundles into individually dispersed CNTs. Contacting CNTs have a cohesion energy per contacting length of ≈30–40 kT nm^−1^, which varies depending on the CNT diameter (**Figure** **3**a).^[^ ^24^ ^]^ Removing/peeling a CNT from a bundle involves overcoming the cohesion energy between approximately three CNT–CNT contacts and therefore requires an energy of up to ≈120 kT nm^−1^.^[^ ^25^ ^]^ This is close to the energy of the CNT carbon—carbon bonds of ≈190 kT, meaning the energy required to separate or destroy CNTs is similar. Perpendicularly contacted CNTs have similar cohesive energies at ≈56–60 kT.^[^ ^26^ ^]^ Separation by sliding is much more difficult, as the force of static friction can dominate cohesion force at overlapping lengths >3 nm.^[^ ^25^ ^]^ Another difficulty is their very long lengths and large aspect ratio. CNTs’ large aspect ratio gives them a large interaction volume while dispersed in liquids. This leads to more contact events with neighboring CNTs during Brownian motion/liquid flow and inevitably leads to more successful adhesion events per unit time. This is exasperated with longer CNTs as they can interact over larger volumes of the medium (Figure 3b). Increased successful adhesion events per time between CNTs results in quicker aggregation of the dispersion. This is similar to higher molecular weight polymers generally being more solid or having higher viscosity, or pulp fiber suspensions thickening at much lower concentrations than similar diameter spherical colloids. It is evident that these issues are why debundling and dispersing CNTs is difficult.

CNTs are argued to never truly be in “solution,” so CNTs separated in a liquid medium are considered a dispersion.^[^ ^27^ ^]^ Most CNT dispersions are actually transient; they eventually aggregate over a certain time period. The less successful readhesion events between adjacent CNTs per unit time, the longer the CNTs will stay dispersed. CNTs are considered successfully dispersed when they remain mostly individualized for long enough to be useful, e.g., weeks, months dispersed instead of minutes. To properly disperse CNTs, both the attractive force must be overcome to separate them, and then readhesion between CNTs must be prevented to keep them dispersed for a long period. Methods of applying energy to separate CNTs into the dispersion can include ultrasonication, rotational sheer forces, and microwave energy. Readhesion is prevented through a few ways: 1) CNTs are placed in a liquid with compatible surface energy/wetting that increases the CNT–solvent attraction to compete with the CNT–CNT attraction. 2) Dispersing agents like surfactants or polymers are added with the CNTs. 3) CNTs are functionalized, which is the chemical modification of their sidewalls to covalently attach functional groups.

#### 2.1.1 CNT Bundle Separation Methods

The most common method employed to overcome the CNT–CNT attractive forces and separate them is through ultrasonication of the CNTs in the dispersion. Ultrasonication produces microbubbles within the dispersing medium, which violently expand and implode in a process called cavitation. Cavitation can produce high temperatures and pressures in small volumes that can separate CNTs into the dispersion and individualize them. It is an easy and effective method but still has issues. The microbubble formation can be relatively random and not concentrated on the CNT aggregates that need to be separated, resulting in sometimes long ultrasonication times (hours) to fully individualize the CNTs. This reduces the commerciality of using ultrasonication to make CNT inks for fabricated CNT devices. The strong forces produced during cavitation can also damage the CNTs and/or shorten them.^[^ ^31^ ^]^ This can impair their electrical/physical properties; a major issue if using the CNT dispersion for device fabrication. Rotational sheer forces can also be applied to separate CNTs into dispersion.^[^ ^32^ ^]^ The localized forces are not as intense and more controlled than ultrasonication. While rotational sheer forces can cause less damage to the CNTs, the separation force is generally not as strong, so other good dispersing conditions are required (good dispersing liquid/good dispersing agents/CNT functionalization).

#### 2.1.2 Dispersing Liquid Medium

Finding appropriate liquids with strong attraction to CNTs to reduce readhesion and disperse CNTs has been difficult for researchers. Testing to determine the best liquids is usually done by attempting to disperse pristine (nonfunctionalized) CNTs in different liquids and determining which liquids can disperse the highest CNT concentration for a functional time period. The best dispersing liquids found mostly have higher surface energies around 35–45 mJ m^−2^, e.g., *N*‐methyl‐2‐pyrrolidone and *N*,*N*‐dimethylformamide (DMF).^[^ ^33^ ^]^ However, higher surface energies are also often associated with much lower evaporation rates. Quicker evaporation of the dispersing liquid is critical for many patterning techniques to increase throughput and reduce subsequent displacement of CNTs from the intended deposition location. Liquids with too high surface energies have poor attraction to CNTs, so unfortunately water with 72 mJ m^−2^ surface energy cannot disperse pristine CNTs. Hansen solubility parameters have also been investigated and are stated to be more predicative than simpler surface energy comparisons.^[^ ^34^ ^]^ Hansen solubility parameters are specific to different types of intermolecular forces that can cause attraction, and include polar, dispersion, and hydrogen‐bonding parameters. Liquid molecules that have aromatic groups with delocalized *π*‐orbitals have proven to be quite attractive to CNTs due to *π*–*π* stacking that occurs between the molecules and the CNTs.^[^ ^35^ ^]^ In fact, this is the cause of the strong attractive force between CNTs in the first place, so it is sensible that liquids that experience *π*–*π* stacking with CNTs are also strongly attractive. Although some liquids can effectively disperse pristine CNTs, the dispersed particle concentrations are still very low compared to other colloidal dispersions. Compare the aspect ratio of nanoparticles to CNTs; the reason is pure statistics. As explained above, CNTs have very long lengths and large aspect ratios, which inevitably result in easier and faster aggregation than lower aspect ratio particles.

Highly charged SWCNTs can also be very well dispersed as polyelectrolytes and are even argued to be true solutions of SWCNTs.^[^ ^36^ ^]^ Intense protonation by superacids^[^ ^36^, ^37^ ^]^ can form SWCNT polycations and negative charging by elemental sodium^[^ ^38^ ^]^ or electrolysis^[^ ^39^ ^]^ can form SWCNT polyanions. These SWCNT polyelectrolytes experience intense repulsive forces, which can even overcome the highly attractive van der Waals forces, providing a method for the true solvation of SWCNTs. They can form the highest concentration SWCNT solutions that can self‐align by forming liquid crystals. However, these SWCNT polyelectrolyte solutions are currently difficult to use in patterning techniques due to their ambient instability or safety/handling issues.

#### 2.1.3 Dispersing Agents

Dispersing agents can be added along with CNTs into a dispersion to prevent readhesion and provide a stable dispersion.^[^ ^23^, ^40^ ^]^ The dispersing agents adhere onto the CNTs and provide steric hindrance or electrostatic repulsion to block CNT–CNT contact.^[^ ^25^ ^]^ They can generally facilitate much higher CNT concentrations in the dispersions than using just liquids for dispersion, because they can prevent direct CNT–CNT contact and possible readhesion. The concentrations are still not as high as other colloid dispersions, however. Surfactants are commonly used for CNT dispersions (e.g., sodium dodecyl sulfate (SDS), sodium cholate (SC), sodium deoxycholate, etc.). The surfactant nonpolar/hydrophobic tail adheres to the CNTs through strong van der Waals forces or *π*–*π* stacking and the polar/hydrophilic head group provides electrostatic or steric repulsion through the liquid medium to prevent contact with neighboring CNTs. The CNT–surfactant binding quality and surfactant availability mainly determine the surfactant dispersing ability.^[^ ^41^ ^]^ Polymer dispersing agents are also common, especially polyfluorenes, polythiophenes, or DNA which have shown chirality selectivity. Polymers can wrap around and adhere to the CNTs, sterically hindering direct CNT–CNT contact or polar functional groups on the polymer can provide electrostatic repulsion. Many other CNT dispersing agents have been explored and they can be suitable for different applications.^[^ ^23b^ ^]^ The dispersing medium properties are still important to consider when using dispersing agents. A dispersing medium must have a high enough dielectric constant to support polar head groups and provide a medium for the electrostatic repulsion. Water is the vastly most common and obvious choice. Also, a low electrolyte concentration and controlled pH must be kept to prevent charge screening which decreases the electrostatic repulsion between polar functional groups.^[^ ^42^ ^]^

While dispersing agents are effective for dispersing CNTs, their presence can impair the electrical properties of CNT devices that have CNTs patterned from dispersions. Surfactants and polymers are less conductive than CNTs or insulating, providing additional resistive barriers along patterned CNT networks.^[^ ^43^ ^]^ The dispersing agents’ lower conductivity can also result in trapping charges within the patterned CNT network. High dispersing agent concentration can also decrease thermal conductivity resulting in unwanted temperature increases in fabricated devices. These issues can be dealt with by subsequently removing the dispersing agents or using more conducting/appropriate dispersing agents. Removing the dispersing agents after patterning is difficult. They adhere well all around the CNTs, and when deposited and dried there is little room for the dispersing agents to move away from the CNTs. Annealing the patterned CNTs can thermally eliminate some dispersing agents,^[^ ^44^ ^]^ however the high temperatures required may also damage other components on the printed devices like the substrate. CNTs are fortunately quite resilient to heat and are not damaged from this process.

#### 2.1.4 Covalent Functionalization

CNTs can be chemically modified to covalently attach chemical functional groups to the CNTs’ sidewalls.^[^ ^23b^ ^]^ These functional groups can again provide steric hindrance or electrostatic repulsion to prevent readhesion between CNTs and keep them dispersed. Covalent functionalization can be very effective at keeping CNTs dispersed and can even keep them dispersed indefinitely. Again, the dispersing medium properties must be considered to ensure effective dispersion. However, covalent functionalization effectively breaks the sp^2^ carbon lattice on the outer wall of the CNT, turning them into sp^3^ carbon atoms that are covalently bonded to the functional group. This interrupts the delocalized *π*‐orbitals and impairs conduction on the CNT outer wall. This can drastically change the electrical properties of SWCNTs, but MWCNTs and DWCNTs are less affected as they can still conduct well through their inner walls.^[^ ^15^ ^]^ Increased covalent functionalization of the CNTs can increase the quality of the dispersion, but clearly also increases the impairment on the electrical properties. Certain functionalization methods, like concentrated acid functionalization, can also shorten CNTs.^[^ ^31^ ^]^ Covalent functionalization has shown to be reversible in some cases through high‐temperature annealing; removing the functional group and repairing the sp^2^ carbon lattice.^[^ ^7^, ^45^ ^]^ However as before, high‐temperature annealing is not always compatible with device fabrication if it damages other components in the devices.

#### 2.1.5 Rheological and Evaporative Effects

Rheological properties of the CNT dispersion must also be adequately controlled to ensure effective patterning.^[^ ^23^, ^46^ ^]^ Increasing CNT concentration in the dispersion can increase the viscosity.^[^ ^47^ ^]^ The viscosity can drastically increase at a certain relatively low critical concentration due to CNTs’ low percolation threshold. Low viscosity of the CNT dispersion can enable faster flows and higher throughput of the CNT patterning technique, so is generally desired. Different CNT patterning techniques require viscosity to be in a certain range to patterning reliably depending on the underlying physics of the patterning process. Surface energies of the liquid medium also need to be controlled to ensure accurate CNT patterning from dispersions.^[^ ^23a^ ^]^ Surface energies must be tuned to allow adequate wetting of the desired patterning regions of the substrate. Surface energies of the liquid medium can also affect the final structure of the deposited CNT patterns due to the surface tension applied during drying/evaporation. After patterning CNT dispersions onto a substrate, the dispersion medium is then generally evaporated to leave CNTs deposited in the desired patterns. However, evaporation of the liquid medium can also apply forces to the dispersed CNTs before they deposit onto the substrate. The coffee ring effect can pull dispersed solids like CNTs to the edge of an evaporating droplet at the three‐phase contact line.^[^ ^48^ ^]^ This occurs due to capillary flows induced by higher relative evaporation at the droplet edge. Conversely, inward Marangoni flows can deposit dispersed solids in the droplet center due to surface tension gradients. These evaporative effects must be considered and controlled to ensure accurate CNT patterning.

#### 2.1.6 Effect of the Chemistry and Physics of CNT Dispersions on Patterning Techniques

The main important but competing aspects when considering the utility of different CNT dispersion patterning techniques is the manufacturability of the technique, and the resulting quality of the CNT patterns. “Manufacturability” of a CNT dispersion patterning technique means the technique's throughput speed, ease of manufacturing, utility, and cost. Manufacturability will depend on the CNT deposition rate and deposition density to increase throughput, and the range of compatible substrates to broaden the technique's utility and reduce manufacturing steps/conditions. CNT “Pattern Quality” is determined by the feature resolution and pitch, alignment, uniformity, electrical/physical properties, etc. Pattern quality will depend on the precise control of the CNT deposition location, the deposited CNT density, and the CNT quality/length/purity. There is a complex interplay between the different properties of the patterning techniques that occur due to the chemical/physical realities of CNT dispersions. These properties are very interdependent and have trade‐offs between beneficial properties. This makes it exceptionally difficult to simultaneously achieve both high‐quality CNT patterns with high manufacturability in a single technique.

**Figure 4:**
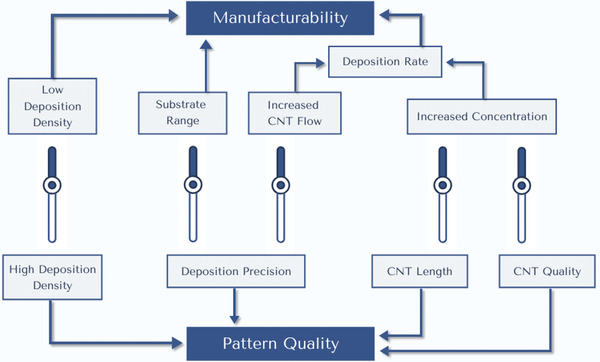
Overview of the interdependent properties of CNT patterning. The sliders in the middle represent the trade‐offs that can be selected between different beneficial properties of the patterning process.

**Figure** **4** illustrates the interdependence of the different properties that affect CNT dispersion patterning, and these will be explained: CNT deposition rate can be increased by increasing CNT concentration in dispersion. Increased CNT concentration is difficult to achieve though. Increased concentrations are achieved through shorter CNTs, increased/better dispersing agents, or covalent functionalization. However, these measures also impair the electrical/physical properties of the CNTs and the resulting CNT patterns. Conversely, longer CNTs and minimal dispersing agents/functionalization will increase the electrical/physical properties of the CNTs and patterns but decrease the CNT dispersion concentration. Additionally, too high CNT concentrations can also reduce the deposition rate due to the drastically increasing viscosity, resulting in an optimal concentration depending on the CNT length/functionalization. CNT deposition rate can also be increased by increasing the flow of CNT dispersion onto the substrate or flow of CNTs through the dispersing medium. However, faster flow can increase CNT aggregation and decreases the control of the deposition location as positioning forces (electrostatics, wettability, EM fields, templates, etc.) are overcome by the faster flow. Reduced control of the deposition location decreases the CNT pattern quality and vice versa. CNTs also have a terminal velocity through the dispersion medium limited by the medium density/viscosity. Alternatively, modifying the substrate with prepatterned features can vastly increase the precise control of the CNT deposition, increasing the CNT pattern quality. However, substrate modifications increase manufacturing steps and can reduce compatible substrates. Increasing the deposited CNT density will generally increase CNT pattern quality but decrease the throughput by slower patterning or increasing patterning runs.

It is important to note that the trade‐offs between desired properties are not linear, nor have they been proven to apply in every situation. Novel methods that can bypass these trade‐offs between properties will provide an avenue to advanced CNT patterning techniques. An ideal CNT dispersion patterning technique will be able to deposit CNTs at a fast rate with great precision, allowing the fabrication of complex, high‐performance, nanoscale CNT devices with high manufacturability.

#### 2.1.7 Analysis of CNT Dispersions

Analysis of the dispersion status of CNT dispersions is critical for patterning and manufacturing so that the quality and uniformity of the CNT dispersions can be monitored. CNT dispersions status refers to the CNT functionalization, CNT structure, CNT concentration, individualization of the CNTs, and the dispersion stability. The reliability of the CNT dispersion patterning techniques is only as reliable as the CNT dispersions used. However, exact quantification of these dispersion properties is actually quite difficult, mainly due to the structural diversity of the CNTs that constitute a typical dispersion. Each dispersed CNT will be a slightly different size with varying defect/functionalization amount and location on the CNT and varying bundling/individualization. Common methods for qualitative analysis of the dispersed CNT structure involve drying the CNT dispersion on a substrate and imaging the deposited CNTs using transmission electron microscope (TEM), scanning electron microscope (SEM), optical, and atomic force microscope (AFM) imaging. Preparing the sample and imaging can be slow, and aggregation can occur making analysis through the images more difficult. CNT functionalization can also be analyzed by Raman spectroscopy,^[^ ^49^ ^]^ Fourier‐transform infrared spectroscopy,^[^ ^50^ ^]^ and other spectroscopy approaches.^[^ ^51^ ^]^ Some other methods such as UV/Vis/IR spectroscopy,^[^ ^50^, ^52^ ^]^ photoluminescence (PL) spectroscopy,^[^ ^53^ ^]^ dynamic light scattering (DLS),^[^ ^34d^ ^]^ and zeta potential^50^, ^51^, ^53^ ^]^ can analyze the liquid dispersions themselves and obtain quantitative data. Both MWCNTs and SWCNTs absorb in the UV/Vis/IR spectrum only when individualized,^[^ ^51^, ^52^ ^]^ and only individualized SWCNTs (not MWCNTs) emit through PL.^[^ ^52^, ^54^ ^]^ This allows researchers to measure the degree of individualization in CNT dispersions using UV/Vis/IR or PL.^[^ ^53b^ ^]^ The aggregation of CNTs in dispersion over time can consequently also be monitored using these techniques.^[^ ^52^, ^55^ ^]^ It should be noted that absorbance/emission is only relative, so only relative difference in CNT individualization in the dispersion can be monitored. Measurement of zeta potential is an effective and common method for determining the stability of electrostatically stabilized colloids like CNT dispersions.^[^ ^50^, ^53^ ^]^ Zeta potential is effectively a measure of the surface potential of the dispersed particle, so higher magnitude of negative or positive charge results in a more stable dispersion due to electrostatic repulsion. Generally, dispersions with zeta potential >15 mV or < −15 mV are considered just stable, while >30 mV or < −30 mV is well‐stabilized. Sun et al. also demonstrated that zeta potential magnitude generally scales with the dispersion quality following a few different metrics.^[^ ^53c^ ^]^ The fractal dimension of CNT dispersions can be analyzed to determine the dispersion status, where a higher fractal dimension means increased fractal structures that cover the dispersion volume/area (increased space filling of CNTs).^[^ ^51^, ^56^ ^]^ Fractal dimension can be measured in a liquid CNT dispersion through light‐scattering measurements.^[^ ^56a^ ^]^ Increased light‐scattering through the dispersion indicates a higher fractal dimension as more light is scattered by the increased coverage of CNT structures. Aggregation results in decreasing fractal dimension and decreased light‐scattering, and this aggregation can thus be monitored over time.^[^ ^56a^ ^]^ Fractal dimension of CNT dispersions deposited on substrates or in solid composites can be measured by analysis of SEM images using the box‐counting algorithm.^[^ ^56b,c^ ^]^ Images of CNT dispersions will show a fractal dimension between 1.0 and 2.0, with 2.0 meaning the whole image is space‐filled with completely debundled CNTs.^[^ ^56c^ ^]^ Fractal dimension analysis can be used for higher concentration dispersions, unlike UV/Vis, PL, or DLS that require dilute dispersions.^[^ ^51^, ^56^ ^]^ Although there is no reported work yet, fractal dimension analysis may also be considered for determining the coverage and deposition uniformity of CNT patterns resulting from the many CNT dispersion patterning techniques discussed in Section 3.

### 2.2 CNT Growth Catalysts and Conditions

There have been many advances in CNT synthesis recently, but also many challenges lie ahead for their effective use in new technologies and applications.^[^ ^57^ ^]^ Many factors of the chemical vapor deposition (CVD) growth process affect the resultant properties of the CNTs synthesized. These factors can include catalyst material, catalyst size/shape, gas composition and pressure, process temperature and duration, substrate properties, and others.^[^ ^58^ ^]^

#### 2.2.1 Growth Catalysts

Common growth catalysts for CNT synthesis include Fe, Ni, Co, and Mo metal nanoparticles. These catalysts are used as they provide a rough template for the initial carbon nanotube cap to form, and a scaffold for the continual growth of the tube. These particular metals are also used due to having high carbon diffusivities allowing the carbon from the chamber to collect and form at the growing end of the CNT. However these metals are liquid/highly mobile at high temperatures, and so do not maintain a particular structure during synthesis resulting in randomized cap structure, and consequently nonspecific chirality of the synthesized CNTs.^[^ ^59^ ^]^ Ceramic, metal oxide, and semiconducting catalysts have also been investigated as CNT growth catalysts.^[^ ^60^ ^]^ Growth catalysts can be synthesized during the patterning step by an innovative method of applying the catalyst in gas phase during the CVD process step. Wei et al. used a vapor mixture of ferrocene catalyst and xylene for CVD growth of CNTs at 800 °C.^[^ ^61^ ^]^ The MWCNTs grew selectively on silica areas patterned on silicon wafer substrates, due to Fe catalyst only forming on the silica. Although they only presented micrometer resolutions patterns, the technique demonstrates a simplified method of growing patterned dense MWCNT forests on silicon wafers. Carpena‐Núñez et al. extended this technique down to nanometer scale by using electron beam lithography (EBL) to modify and pattern sapphire wafers.^[^ ^62^ ^]^ The modified sapphire areas were able to convert ferrocene vapor precursor into Fe catalyst for patterned CNT growth. In a similar method, Chen et al. used a vaporized iron phthalocyanine catalyst during the CVD process, and focused ion beam (FIB) etched trenches in a silicon wafer caught the catalyst and grew CNTs from the patterned trenches.^[^ ^63^ ^]^

#### 2.2.2 CNT Diameter

The diameter of CVD‐grown CNTs are highly dependent on the diameter of the catalyst metal nanoparticles used. Catalyst nanoparticles act as “seeds” where diffusing carbon atoms assemble into tubes wrapped around and protruding from the catalyst nanoparticle, and so the inner diameter of the grown CNTs closely matches the diameter of the catalyst nanoparticles. During the high‐temperature CVD process, metal atoms on the substrate can diffuse and coalesce. Controlling the metal atom coalescence is crucial to synthesize metal nanoparticles of required diameter to catalyze CNT growth. Metal nanoparticles that are more densely packed on the substrate before the CVD process will coalesce into larger particles faster due to having a shorter required distance to diffuse. Consequently, patterning of the catalyst nanoparticles at the nanoscale with adequate spacing can help to control catalyst nanoparticle coalescence and help control grown CNT diameter.^[^ ^64^ ^]^ Longer heating times may also cause larger nanoparticles due to a longer time for diffusion and coalescence. Similarly, higher temperatures will increase the speed of diffusion and hence speed of coalescence. Introduction of H_2_ before the carbon‐source gas may accelerate coalescence, as H_2_ can reduce any passivating oxide layer on the metal/nanoparticles.^[^ ^65^ ^]^

#### 2.2.3 CNT Quality and Length

The quality and purity of grown CNTs can be controlled by a water‐assisted CVD process.^[^ ^66^ ^]^ Hata et al. demonstrated that the addition of water vapor during the CVD process enhances catalyst activity, resulting in 99.98% pure, super dense, vertically aligned SWCNT forests from a variety of catalysts and substrates.^[^ ^66^ ^]^ Water vapor is a weak oxidizer and they suggested that it removes amorphous carbon, without damaging SWCNTs. Removing the amorphous carbon from the catalysts increases their activity and lifetime and keeps the grown CNT forests free from impurities. Growth speed increased up to 2.5 mm long SWCNTs grown in 10 min, with a large potential for scalability.^[^ ^67^ ^]^ Further research by Zhang et al. suggested that the oxygen content in the water helps to scavenge reactive H species.^[^ ^68^ ^]^ The H species can inhibit formation of sp^2^‐carbon structures, and so oxygen species in the reaction chamber can prevent this inhibition. A similar effect is found when using ethanol as the carbon feedstock. Amama et al. demonstrated that water vapor can also suppress Ostwald ripening (coalescence) of metal catalyst nanoparticles during CVD due to decreasing metal diffusion rates.^[^ ^69^ ^]^ Suppression of Ostwald ripening and reliable catalyst diameters can also be achieved by roughening of the substrate surface and densification of the substrate bulk.^[^ ^70^ ^]^

The length of the CVD‐grown CNTs is generally proportional to the CVD reaction time (but not necessarily linearly).^[^ ^61^, ^71^ ^]^ Larger catalytic patterns also produce longer CNTs than patterns with finer features, however CNTs from larger patterns will have lower number of walls.^[^ ^72^ ^]^ This is caused by the interplay between the availability of carbon feedstock within the vicinity of the catalyst nanoparticles (local partial pressure), and the thermodynamically preferred growth process. Patterning of CNTs can thus be used to control CNT length and wall number. Similarly, increased flow rate of the carbon feed stock can increase the growth rate/CNT length due to increased available carbon. CNT length may also equal the length of the deep pores in porous template substrates that are used for templating patterned CNT growth (Section 5.1).^[^ ^73^ ^]^

#### 2.2.4 Process Temperature

Generally, temperatures >500 °C are required for the CVD growth process of CNTs. This is a severe restriction for some applications of CNT growth catalyst patterning, as high temperature‐sensitive materials cannot already be present on the devices during CNT synthesis. However using certain carbon feedstocks that decompose at lower temperatures, plasma decomposition techniques, and catalyst engineering, <500 °C growth temperatures can be achieved under certain conditions.^[^ ^74^ ^]^ Some studies have shown that using microwave synthesis, catalyst nanoparticles can be selectively heated to the required CNT growth temperature, while keeping the rest of the substrate at lower temperatures <150 °C.^[^ ^75^ ^]^ Metal nanoparticles are selectively heated because they can absorb the microwave frequencies and heat up, while the rest of the substrate is microwave transparent or reflective. This allows for synthesis of patterned CNTs on a device where other heat‐sensitive parts of the device have already been fabricated, and the microwave synthesis process takes only minutes. However, this technique currently still has issues with synthesizing high quality, monodisperse, and long CNTs, as heat dissipation through the substrate has not been fully investigated and understood. In a similar way MEMS (micro electro mechanical systems) microheaters on the substrate can produce localized resistive heating at desired location on the substrate, which adds more precise electronic control to the heating method.^[^ ^76^ ^]^ Alternatively, process temperature can be locally controlled by using laser‐assisted CVD synthesis.^[^ ^77^ ^]^ This technique directs a laser beam to a particular location on the substrate to efficiently heat the substrate at that location, avoiding possibly thermally sensitive parts of the substrate. The heating is much quicker than conventional CVD too, lowering processing time and the polarization of the laser can encourage directional growth. Localized heating is useful for pre‐synthesis CNT patterning techniques, making them compatible for fabricating a wider variety of CNT devices.

### 2.3 Sorting and Purification

SWCNT chirality determines its band gap (Section 1.1). Common SWCNT synthesis methods result in a natural wide range of chiralities and consequently a wide range of different band gaps. Different band gaps vary conductivities from semiconducting to metallic as well as produce uniquely different optical spectra. However, different applications will require different conductivities and particular optical spectra, therefore it is critical to sort or selectively grow SWCNTs by chirality for each particular application. Highly semiconducting‐pure SWCNT dispersions are extremely useful for electrical applications like CNTFETs, where any metallic components in the channel of a CNTFET can severely impair the FET qualities. SWCNTs can also be used for conducting components where metallic SWCNTs are more useful. Optical and photonic applications of SWCNT require control of the optical spectra, and so chirally pure SWCNTs providing more defined optical spectra is critical. CNTs can also be sorted by length, which is useful for certain applications. MWCNT have mostly metallic properties due to the averaging properties of multiple walls. DWNCTs are more complex and can require alternative sorting strategies, particularly if the inner wall chirality is to be sorted.^[^ ^15^ ^]^

#### 2.3.1 SWCNT Sorting

SWCNT sorting methods have made tremendous advances recently with some sorting methods able to acquire purified SWCNT dispersions with 99.99% semiconducting chirality SWCNTs.^[^ ^14^, ^78^ ^]^ Selective adhesion and wrapping of rationally chosen molecules (polymers,^[^ ^79^ ^]^ surfactants,^[^ ^80^ ^]^ DNA^[^ ^81^ ^]^) on target chirality SWCNTs is an effective and scalable technique for chirality sorting in dispersions. The sorting molecules that adhere to SWCNTs also double as the dispersing agent to create stable SWCNT dispersions. The selectivity of different molecules to different chirality SWCNTs is dependent on the matching of structure and chemistry that can increase adhesion. SWCNTs can be sorted by chirality and length with separation techniques including density gradient ultracentrifugation,^[^ ^82^ ^]^ gel column chromatography,^[^ ^83^ ^]^ ion exchange chromatography,^[^ ^84^ ^]^ aqueous two‐phase extraction,^[^ ^85^ ^]^ DEP,^[^ ^86^ ^]^ and gel electrophoresis.^[^ ^87^ ^]^ These techniques are close to reaching the 99.9999% semiconducting purity threshold that is required for CNTFETs to compete with conventional semiconductor materials (Section 7.1).^[^ ^78^, ^88^ ^]^ The ability to easily acquire chirally pure SWCNT dispersions with new sorting techniques is a major advantage of techniques that pattern CNTs from dispersions over pre‐synthesis patterning techniques.

#### 2.3.2 Chiral‐Specific SWCNT Growth

Chirality‐specific SWCNT growth has also advanced significantly recently, with specific SWCNT chirality purity of over 97% being achieved from controlled growth conditions.^[^ ^58^, ^89^ ^]^ These advances allow the pre‐synthesis patterning techniques to also use chiral‐specific CNTs. The type and shape of catalysts used are the major influence for chiral‐specific SWCNT growth, although carbon source, gas composition, temperature, pressure, and growth time are also influences.^[^ ^89^, ^90^ ^]^ Controlling the shape/size of the metal nanoparticle catalysts requires control/suppression of coalescence and diffusion of the catalysts through substrate stabilization or catalyst composition. Using metal alloy catalysts that are more solid at high temperatures provides a fixed scaffold during growth to produce specific end‐cap structures.^[^ ^89a–c^ ^]^ Molecular “seeds” of pre‐sorted short CNT segments or sorted CNT end‐caps have also been investigated as chiral‐specific CNT growth catalysts.^[^ ^57^ ^]^ Perhaps in further studies these molecular seeds can also be patterned onto substrates to synthesize chiral pure CNTs in patterns for future devices. Recently, Zhu et al. reported obtaining semiconducting CNTs with a remarkable 99.9999% purity via growth rate differences between the chiralities when growing ultralong CNTs.^[^ ^91^ ^]^ Metallic CNTs have a tenfold faster decay rate, allowing the researchers to effectively find only semiconducting CNTs growing longer than 15.4 cm along the substrate. However, this technique clearly requires a large growing area on the substrate to synthesize the purified semiconducting CNTs.

#### 2.3.3 Selective Destruction of SWCNTs

SWCNTs of particular chiralities may be removed after synthesis/deposition by different methods including selective electrical breakdown,^[^ ^92^ ^]^ laser irradiation,^[^ ^93^ ^]^ thermocapillary effects,^[^ ^94^ ^]^ chemical etching,^[^ ^95^ ^]^ gas phase etching,^[^ ^95^ ^]^ and others to achieve semiconducting purity of ≈99.99% or over.^[^ ^58^, ^96^ ^]^ However, selective destruction of SWCNTs to enhance chiral purity will result in lower SWCNT density. An interesting technique presented by Kanungo et al. chemically converts metallic SWCNTs through cycloaddition reactions into semiconducting SWCNTs, thus maintaining high CNT densities.^[^ ^97^ ^]^

### 2.4 CNT Networks and Alignment

CNTs can have excellent conductivities and mobilities along individual tubes but also have high intertube junction resistance. Resistance along patterned CNT networks is therefore mostly due to these intertube junctions. More conductive pathways that have fewer intertube junctions will consequently decrease resistance through CNT networks. This can be achieved with longer CNTs, increased CNT density, and aligning CNTs in the preferred direction of conduction. Long CNTs are more difficult to manipulate and disperse effectively (Figure 3b), increasing the difficulty of fabricating high‐quality conductive devices with long CNTs at sufficient densities. Long CNTs can be grown on substrates for use in devices, but often must be patterned after synthesis. CNT density in the network must be at least above the percolation threshold to ensure a conduction path through the network. Fortunately, CNTs’ percolation threshold is very low due to their very large aspect ratio (e.g., ≈0.57% required surface coverage for 1000 aspect ratio CNTs).^[^ ^23^, ^98^ ^]^ Alignment of CNTs in the preferred conduction direction is an effective method to reduce CNT network resistance. Straight conducting pathways are shorter and pass less intertube junctions than zig‐zagging random pathways (Figure 3c). Slightly aligned networks also increase the percolation pathways, consequently reducing the percolation threshold and increasing conductivity.^[^ ^99^ ^]^

Recent intense research into alignment has produced many useful methods of alignment during or after CNT synthesis.^[^ ^58^, ^78^ ^]^ These methods most often align CNT arrays evenly over whole substrates. These arrays are used to fabricate high‐quality CNT devices due to their superior electrical/physical properties and high densities. However, these arrays must then be further processed and patterned after alignment to achieve their specific function in devices. Techniques that can pattern whole substrate CNT arrays are a smaller subset of the CNT patterning techniques where the patterning step is post‐deposition. Commonly, post‐deposition patterning is done by oxidation etching through lithographically defined photoresists (Section 4.1). It is a simple and reliable technique that is compatible with complementary metal oxide semiconductor (CMOS) manufacturing processes but can be slow and costly for mass manufacturing. Importantly though, some CNT patterning techniques can also produce aligned CNTs during the patterning process (Section 2.4.3).

#### 2.4.1 Random Networks

Random networks of unaligned CNTs are commonly used as components in devices and are sufficient for a variety of purposes.^[^ ^11^ ^]^ Most high throughput and lower resolution CNT patterning technique will produce random networks. For many simpler applications that do not require higher performance and prefer less cost and flexibility, random networks from high throughput patterning techniques are suitable. However, for higher performance devices, the increased resistance in random networks can be prohibitive. High variability of the quality of random CNT networks is an issue when attempting to fabricate consistent and reliable devices.^[^ ^11^, ^100^ ^]^ Random CNT networks also tend to have more charge traps throughout the network, increasing resistance and resulting in increased recombination of charge carriers in p‐n junction devices like solar cells.^[^ ^101^ ^]^ Resistance in random CNT network lines can increase with decreasing line width and thickness (Figure 3d).^[^ ^28^ ^]^ This is unsurprising, as the same effect occurs in common metal wiring/lines like Cu. Conversely though, sub monolayer CNT networks can actually experience increasing conductivity/mobility with decreasing line width due to self‐alignment (Section 2.4.3, Figure 3e).

#### 2.4.2 Alignment of CNTs from Dispersions

Shearing of the CNT dispersion can produce strong alignment along the shear direction. The forces of the shearing liquid push the CNTs in alignment over time, with greater shear forces pushing the CNTs to alignment faster. There are diverse ways to induce this shear force, including by spin‐coating,^[^ ^102^ ^]^ blade‐coating,^[^ ^103^ ^]^ and dry shear aligning.^[^ ^38^, ^104^ ^]^ Shearing can be applied quickly and often simultaneously with other fabrication techniques. Most shear‐aligning techniques are performed over whole substrates, rather than in patterned areas. Consequently, shear‐aligned CNT networks are commonly patterned post‐deposition.

Many effective and popular alignment techniques crowd CNTs in dispersion together causing densification, tight packing, and alignment of the CNTs. Densification of the CNTs without aggregation allows them to form essentially liquid crystals, which have approximately periodic structure in dispersion. However, high concentration of CNTs in dispersion without aggregation is difficult to achieve as stated earlier in Section 2.1. The most common solution is densifying the CNTs on the 2D surface of the dispersing liquid medium, often just before the CNTs are deposited on a substrate so aggregation is not an issue. Alignment of CNTs occurs at the evaporating contact line between dispersion and substrate due to the increased evaporation at the contact line causing additional liquid and CNTs to flow toward the contact line (evaporation‐driven self‐assembly,^[^ ^105^ ^]^ floating evaporative self‐assembly^[^ ^106^ ^]^). This is a simple alignment technique but does rely on the evaporation rate which is slow. CNTs films can be densified and aligned on a liquid surface through the Langmuir–Schaefer method which can achieve essentially full monolayer surface coverage of aligned CNTs at 500 CNTs µm^−1^.^[^ ^107^ ^]^ Very dense and aligned CNT films have recently been produced through slow controlled vacuum filtration.^[^ ^108^ ^]^ The CNTs can be pseudo 2D confined by repulsion from the filter membrane surface and liquid flow, allowing them to densify and align with the 2D plane of the filter. This is a highly effective aligning technique with ±1.5° global alignment and 10^6^ CNTs µm^−2^ achievable. However, these CNT film techniques require subsequent patterning of the film to be used as most components in devices (Section 4), where patterning of CNT films has had limited investigation currently. An interesting densification alignment technique uses aligned CNT “rafts,”^[^ ^109^ ^]^ CNTs are densified into rafts by attractive depletion forces caused by entropic effects at high surfactant concentration.

Electric fields can be used to align CNTs in a medium along the electric field direction. Constant electric fields will attract and align all CNTs as charge carriers in the CNTs collect at separate ends of the CNTs and cause a net positive attractive force along the increasing electric field gradient. Oscillating electric fields generated by AC current in the electrodes are different, as the CNTs’ response to the oscillating field is dependent on their conductivity and relative permittivity (dielectric constant) and the oscillating frequency. CNT DEP patterning uses these oscillating electric fields to not only align CNTs but also precisely place CNTs on substrate for patterning. Details of manipulating CNTs with oscillating electric fields are explained in Section 3.3.

#### 2.4.3 Self‐Alignment of CNTs Patterned from Dispersions

A simple method of CNT alignment that can be achieved simultaneously with patterning CNTs from dispersion is to reduce the width of patterned line features to less than the average length of the CNTs.^[^ ^2^, ^30^, ^78^, ^110^ ^]^ CNTs that are longer than the line widths clearly cannot deposit perpendicular to the patterned lines, as they are geometrically confined within the patterned lines (Figure 3f). The greater the ratio of CNT length to line widths, the greater the alignment parallel with the patterned lines, as the increasing confinement only allows increasingly aligned orientations. This alignment can produce an unconventional effect in monolayer CNT films, where decreased line widths result in increased conductivity and mobility, in contradiction to CNT patterning techniques that result in random CNT networks, post‐deposition patterning, or conventional conducting materials.^[^ ^29^ ^]^ Lee et al. demonstrated through experiment and simulations that thinner line widths of CNT lines patterned through deposition of CNTs from dispersion on SAM‐patterned substrates resulted in a much higher alignment of the CNTs in the network and consequently increased conductivity/mobility (Figure 3e). They found the scaling behavior of (conductivity) ∼ (line width)^−0.2^, in the region from ≈8 µm width to a saturation of ≈80 nm width. Thinner width lines resulted in much higher *I*_on_/*I*_off_ from fabricated CNTFETs. Note however that these results were from sub monolayer (<1 nm) low density SWCNT films. Thicker, multilayer SWCNT networks with natural metallic proportion (33%) would easily form metallic percolation paths through the network and result in metallic SWCNT lines with higher conductivity and low *I*_on_/*I*_off_.^[^ ^110h,i^ ^]^ Aligned, multilayer SWCNT networks also experience more typical conducting behaviors where decreasing line width increases resistance.^[^ ^110h^ ^]^ Decreasing line width for multilayers networks to <0.5 µm can drastically increase the resistivity, as the semiconducting behavior dominates from lack of metallic conducting pathways.^[^ ^110h^ ^]^ Somu et al. presented simulations showing that whether the SWCNT line expresses semiconducting or metallic properties is dependent on both the SWCNT density and the line widths (Figure 3g).^[^ ^30^ ^]^ Clearly, tuning the line widths and SWCNT density is important to obtain SWCNT lines with properties appropriate to the application. CNT self‐alignment is a particular advantage for patterning CNTs from dispersions at the nanoscale.

## 3 Patterning from CNT Dispersions

There are a large variety of techniques to deposit pre‐synthesized CNTs from dispersions into desired patterns on substrates. The various methods guide the CNT dispersions into desired patterns onto substrates and adhere/deposit CNTs at the desired locations. CNTs patterned from dispersions effectively decouple the CNT synthesis step and the patterning step, allowing for a wider range of CNT synthesis methods to be employed to obtain a wider range of CNT types and qualities. CNTs synthesized before patterning can also be sorted/filtered/purified by chirality, length, and diameter, further increasing the control of CNT type and quality that are used for these patterning techniques. The other advantage of patterning CNT dispersions is the simplicity of the methods. They generally involve simple processes done at ambient temperatures, low costs, and are amiable to large area and/or high‐throughput production. Flexible or polymer substrates can even be patterned due to the low‐temperature requirements and processability.

Some CNT dispersion patterning methods are high throughput and/or roll‐to‐roll (R2R) techniques, which are used for high‐throughput manufacturing of electronics and enables wider and cheaper access of future electronics.^[^ ^1^, ^6^, ^111^ ^]^ These high‐throughput techniques include: inkjet printing,^[^ ^1^, ^112^ ^]^ electrohydrodynamic jet printing,^[^ ^113^ ^]^ aerosol jet printing,^[^ ^114^ ^]^ gravure printing,^[^ ^115^ ^]^ screen printing,^[^ ^116^ ^]^ flexographic printing,^[^ ^117^ ^]^ and others. These techniques can easily pattern conductive CNT networks onto flexible substrates and so they are often used for fabricating flexible electronics. However, these faster throughput patterning techniques generally suffer from poorer lateral resolution of the printed patterns (>10 µm) and poorer electronic properties of the patterned CNTs. The general trade‐off though is between resolution and throughput, where increased throughput speed results in decreased pattern resolution. Device minimization is consequently restricted with larger lateral resolution patterning techniques, but this is acceptable for particular applications. CNT networks patterned from high throughput techniques are almost exclusively randomly aligned networks with decreased conductivities/mobilities (Section 2.4.1). Faster throughput can also result in decreased deposited CNT density, as the CNT “inks” used have limited concentration. These issues prevent high‐throughput techniques from fabricating higher‐performance devices.

There are techniques though that can pattern CNTs from dispersions with nanoscale resolution. This section details the currently explored and invented nanoscale resolution techniques and assesses their pros and cons. Lessons learned from these techniques can guide device fabricators in selecting the appropriate technique as well as frame current knowledge of nanoscale patterning of CNT dispersions. Future research into patterning CNT from dispersions should explore methods to circumvent the apparently inherent trade‐offs currently present (Section 2.1.6), to achieve both high throughput and nanoscale precise patterning of the CNTs on substrates.

### 3.1 Substrate Modification Patterning

One of the most common CNT nanoscale patterning techniques involves modifying the surfaces of substrates in patterns of CNT‐attractive and CNT‐repelling areas. A CNT dispersion is then placed across the whole substrate, and the CNTs are attracted to the CNT‐attractive areas only, creating a controlled pattern of CNTs on the substrate. Commonly this means patterning hydrophilic and hydrophobic regions on the substrate, where CNT dispersions are attracted to the hydrophilic regions. Surface modifications can include patterning self‐assembled monolayers (SAMs),^[^ ^2^, ^110^, ^118^ ^]^ oxidizing the substrate,^[^ ^118^, ^119^ ^]^ or otherwise chemically modifying the substrate surface.^[^ ^120^ ^]^ The patterned modifications of the substrates are often done with common patterning techniques including photolithography,^[^ ^118g,118j^ ^]^ electron beam lithography,^[^ ^2^, ^110^, ^118^, ^120^ ^]^ contact printing (CP),^[^ ^110^, ^118^ ^]^ and even probe lithography.^[^ ^110^, ^118^, ^121^ ^]^

**Figure 5:**
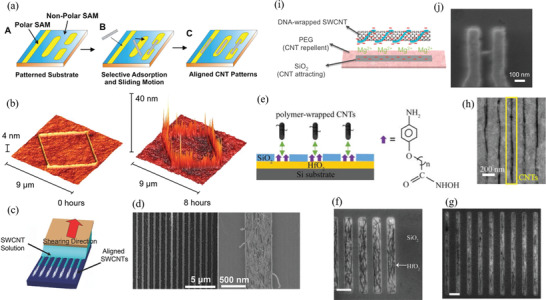
a) Figure depicting adhesion of CNTs onto polar hydrophilic patterned SAM on substrates, followed by “sliding” of CNTs protruding on hydrophobic regions toward hydrophilic regions. Reproduced with permission.^[^ ^118e^ ^]^ Copyright 2006, AIP Publishing. b) AFM image of Si substrate patterned by etching an SAM resist through probe lithography, and then subsequent amide attachment of CNTs to the patterned area. Reproduced with permission.^[^ ^118h^ ^]^ Copyright 2007, Elsevier. c) SWCNT solution shearing over nanoscale SAM patterned substrate. d) Scanning electron microscope (SEM) images of nanoscale, aligned SWCNT lines patterned using the technique. Reproduced with permission.^[^ ^110j^ ^]^ Copyright 2015, Wiley‐VCH. e) Polyfluorene‐sorted semiconducting SWCNTs deposited via complexation with diazonium head group on SAM attached to HfO_2_ regions. SEM images of SWCNTs patterned inside f) 100 nm and g) 50 nm wide trenches, and h) SWCNTs that were deposited in the 50 nm trenches after etching of SiO_2_. Reproduced with permission.^[^ ^133^ ^]^ Copyright 2017, American Chemical Society. i) DNA‐wrapped semiconducting SWCNTs attracted to hydrophilic SiO_2_ patterned regions mediated by a magnesium ion charge inversion layer. j) SEM image of a single SWCNT CNTFET fabricated with the patterning technique. Reproduced with permission.^[^ ^126c^ ^]^ Copyright 2016, American Chemical Society.

Substrate modification patterning often can attain nanoscale or sub‐micrometer resolution, but efficiency of CNT placement is often much lower at that resolution.^[^ ^2^, ^110^, ^118^, ^119^ ^]^ Alignment of the CNTs on the patterned regions is not guaranteed, but a simple method of alignment is to reduce the width of patterned lines to less than the average length of the CNTs (Section 2.4.3).^[^ ^2^, ^29^, ^110^, ^122^ ^]^ Some long CNTs may partially deposit over the edge of the patterned lines into the undesired regions, depending on conditions. This effect can increase line edge roughness of the patterned line features. However, Im et al. presented experiments demonstrating that long CNTs hanging over into unattractive regions can “slide” onto the patterned attractive regions (**Figure** **5**a).^[^ ^118e^ ^]^ CNTs’ freedom to bend and rotate aids their ability to reconfigure fully onto the patterned attractive regions, allowing even >1 µm long CNTs to be confined to <100 nm wide patterned features.^[^ ^121^ ^]^ CNTs can even be bent (which increases elastic energy) to stay confined to the attractive patterned regions, showing that the CNTs are attracted to the patterned regions due to their low potential energy. Smaller CNTs have a higher adsorption probability onto patterned hydrophilic regions than larger CNTs, due to the faster movement of smaller CNTs in the dispersion.^[^ ^110d^ ^]^ Dispersing surfactant chemistry is also important to consider for increased CNT adsorption.^[^ ^123^ ^]^ Certain residual surfactants in the dispersion can densely pack onto the patterned hydrophilic regions, preventing CNT adhesion. Other surfactant types (e.g., steroid‐based) have poor stacking on the hydrophilic regions, allowing CNTs to interact and adhere effectively to the patterned hydrophilic regions. Modification of the substrate may be substantially disruptive to the substrate and not possible when making devices with multiple layers and different material requirements for different regions. There is also possibly uneven or incomplete coverage of CNTs along the patterned regions.^[^ ^2^, ^29^, ^110^, ^119^ ^]^ The CNT patterns made are often sub‐monolayer or monolayer, as the attraction between the substrate and CNT drives the adhesion.^[^ ^29^, ^124^ ^]^ These thin films can have higher resistances than multilayer, thicker CNT patterns that are formed with other patterning techniques.^[^ ^110h,i^ ^]^ However, monolayer CNT patterns can be desirable if semiconducting channels are required as multilayers can cause negative screening effects.

Many SAM methods have been developed, with common SAMs including 1‐octadecanethiol (ODT) or octadecyl trichlorosilane (OTS) as hydrophobic regions, and aminopropoyl trimethoxysilane (APTMS), aminopropoyl triethoxysilane (APTES), 16‐mercaptohexadecanonic acid (MHA), or cysteamine as hydrophilic regions.^[^ ^110^, ^118^, ^119^, ^121^, ^125^ ^]^ Hydrophilic regions may also just be bare SiO_2_, glass, Au, or Al substrate, with hydrophobic SAM regions blocking CNT adhesion.^[^ ^110^, ^118^ ^]^ CNTs are generally functionalized to be more highly charged, as the Coulombic interaction between oppositely charged SAMs and CNTs increases the adhesion forces. Common functionalizations include surfactants,^[^ ^2^, ^110^, ^118^ ^]^ polymer or DNA wrapping,^[^ ^118^, ^126^ ^]^ or chemically modifying the CNTs surface with acid or amine groups.^[^ ^118h^ ^]^ Functionalization is not always necessary though. Pure CNTs have been shown to adhere well to hydrophilic SAM regions, and also may have higher deposition density than surfactant‐functionalized CNTs.^[^ ^110^, ^121^ ^]^ The deposited CNT density can be increased by choosing a solvent/SAM combination where the solvent only wets the hydrophilic SAM, as the dispersed CNTs localize to those regions.^[^ ^118^, ^121^ ^]^ Thickness control of the SAM layer is also important, as nonmonolayer thickness can reduce adhesion between the CNTs and SAMs.^[^ ^110b^ ^]^ This is because the end groups of the SAMs no longer cover the entire substrate surface, changing the chemistry of the surface. Zhang et al. recently reported simulations of the mechanism of surfactant‐dispersed SWCNTs self‐assembling onto SAM‐patterned substrates.^[^ ^124^ ^]^ They showed that competition with H_2_O molecules can limit adhesion of SWCNTs on the hydrophilic SAM regions until the adsorbed H_2_O layer evaporates from the substrate. This means drop‐coating and blade coating, which encourage faster and constant solution evaporation, can result in higher deposited SWCNT density than spin coating and dip coating. They also demonstrated that a “shielding effect” from the dispersing surfactants prevents adhesion of SWCNTs to the SAM substrate within a radius of an already adhered SWCNT. The shielded radius is proportional to the length of the dispersing surfactant. This results in monolayer or less deposition and might also be exploited in the future to control spacing between deposited SWCNTs by controlling the length of the dispersing surfactants.

Burghard et al. first used SAMs for CNT patterning.^[^ ^118a^ ^]^ Using conventional lithography, the patterned metal electrode regions onto a SiO_2_ substrate. They selectively silanized a positively charged amine‐terminated SAM onto the SiO_2_ regions, and then subsequently attached the negatively charged 3‐mercaptopropionic acid SAM onto the Au regions. The surfactant that was dispersing the CNTs had negatively charged groups, and so attracted the CNTs to the positively charged SAM on the SiO_2_ regions and repelled the CNTs from the negative Au regions. This allowed them to deposit MWCNTs selectively onto nanoscale regions. Liu et al. patterned a methyl‐terminated SAM which was hydrophobic and positively charged amine SAM which was hydrophilic using AFM and EBL.^[^ ^118b^ ^]^ The negatively charged surfactant‐dispersed CNTs were spontaneously attracted and adhered via Coulomb interactions to the positively charged amine regions and repelled from the hydrophobic regions, revealing CNT patterns on the substrate. They could even controllably adhere a single CNT between two electrodes, measuring its conductance. Other groups reported depositing negatively charged amine SAMs through EBL‐patterned resists onto SiO_2_ substrates, and using the resist as the repelling hydrophobic region.^[^ ^110^, ^118^ ^]^ Rao et al. demonstrated a massive advancement to this technique in 2003 by demonstrating large‐area scaling on substrate areas of 1 cm^2^ simultaneously. They patterned hydrophilic amine or carboxyl‐terminated SAMs, and hydrophobic methyl‐terminated SAMs on gold substrates using contact printing (CP) and dip pen nanolithography (DPN).^[^ ^110c^ ^]^ After submerging the patterned substrate in CNT dispersion, CNTs were patterned on the attractive hydrophilic regions. Using lower CNT concentration suspensions, they were able to adhere single CNTs to single hydrophilic regions with ≈90% efficiency over a whole substrate.

Auvray et al. tuned the chemistry of the patterned SAM to optimize the electrical contact at CNT/electrode and CNT/substrate interfaces for electrical devices like CNTFETs.^[^ ^125^ ^]^ Selective protonation or deprotonation of the SAM can modify and improve carrier injection and consequently doping in the CNTs. Through this method, they could reduce Schottky barriers established at CNTs/metal electrodes interfaces to near ohmic contact. This is an extended functionality that bare silicon wafer substrates do not have. They applied trifluoro‐acetic acid in vapor phase to protonate the amino end group of the SAM, resulting in a sub‐threshold slope for the fabricated CNTFETs that was tenfold better than the untreated SAM devices and twofold better than bare SiO_2_ devices. Myung et al. presented a “lensing” effect, where SAM patterns with density gradients could focus the adhesion of CNTs to the middle of the patterned hydrophilic SAM regions.^[^ ^110e^ ^]^ This technique could essentially increase the patterning resolution down to 10s of nanometer scale while only using micrometer resolution SAM patterning techniques. The SAM density gradient was created by applying the CP stamp that was printing the hydrophobic SAM negative patterns for a longer time than normal. This allowed hydrophobic SAM to laterally diffuse away from the negative patterns, creating a density gradient of hydrophobic SAM into the desired patterned areas. When the CNT‐attractive hydrophilic SAM was backfilled into the desired patterns, a density gradient was created with the highest density in the center of the patterns. The depositing CNTs are subsequently attracted to the more attractive region in the center of the patterns due to energy minimization, focusing the CNTs. A similar lensing effect was reported by Sharma and Strano, however, the cause was different.^[^ ^118k^ ^]^ They deposited aqueous SWCNT surfactant dispersions onto substrates with periodic lines of hydrophilic SAM between hydrophobic SAM regions. The dispersion formed cylindrical droplets along the hydrophilic lines, due to the strong attractive interfacial force between the water/hydrophilic region, and the strong repulsive force between the water/hydrophobic region. They found that hydrophilic lines with <1 µm widths have SWCNTs deposited aligned in the center of the line, whereas >3 µm width lines had SWCNTs deposited on the edges of the lines. They suggested that the reason was the evaporation mechanism. Thinner lines experienced a depinning of the droplet contact line during evaporation, resulting in radially inward flows to the line center. Thicker lines experienced pinned contact line evaporation, resulting in radially outflows and edge deposition similar to the coffee ring effect.^[^ ^127^ ^]^ Both evaporation modes result in high alignment of the deposited SWCNT with the line direction.

DPN is a powerful technique to pattern SAMs on the target substrate allowing for arbitrary patterns to be patterned at nanometer resolution (see Section 3.6 for details).^[^ ^118^, ^121^ ^]^ Wang et al. patterned <100 nm resolution patterns of hydrophilic SAM MHA onto Au substrates using DPN.^[^ ^121^ ^]^ Dispersed CNTs could then strongly adhere to the patterned hydrophilic SAMs via strong van der Waals forces. The adhesion force to the MHA SAM was strong enough to tightly bend 1–3 µm long CNTs in circular patterns with down to 650 nm radius of curvature. Using Monte Carlo simulations, they calculated that only ≈19% of the length of an SWCNT needs to interact with the hydrophilic SAM pattern to successfully adhere to the substrate. MHA SAM was also calculated to adhere CNTs much better than amine‐terminated SAMs, resulting in approximately tenfold thinner line widths required for adhesion, and tenfold increased CNT coverage density. Flavel et al. presented a series of reports demonstrating an interesting probe lithography‐based method to pattern silicon substrates for patterned CNT deposition.^[^ ^118^, ^119^ ^]^ A methyl‐terminated hexadecyl trichlorosilane SAM was applied to a silicon substrate as a resist for electrochemical anodization using an AFM probe. The probe was traced along the substrate in the desired pattern while applying a voltage bias, which etched away the SAM resist and resulted in hydroxyl‐terminated silica regions. The amine‐terminated 3‐aminopropyltriethoxysilane (APS) could also be self‐assembled onto the etched regions. Carboxylic acid group‐functionalized SWCNTs could then covalently bond very selectively to the etched regions via condensation reactions to form strong ester or amide linkages (Figure 5b). However, higher yields of attached SWCNTs were achieved through amide linkages due to easier condensation reactions to form amides. Increased scanning speed and decreased applied voltage resulted in decreased patterned line width. The covalently bonded SWCNTs were also very vertically aligned, which is useful for particular applications like chemical sensing. They could attach ferrocene to the end of the attached SWCNTs to electrochemically detect redox reactions to the ferrocene at spatially defined regions.^[^ ^119a,b^ ^]^ The patterned vertically aligned SWCNTs could also be used as a frame for high‐resolution patterned deposition of conducting polymers like polyaniline.^[^ ^119c^ ^]^ A similar method was presented by Druzhinina et al., where they used a probe to oxidize OTS SAM to form nanoscale patterns of SAMs with carboxylic acid head groups.^[^ ^119d^ ^]^ These patterned polar head groups could then adhere surfactant‐dispersed CNTs directly, or covalently attach APTMS polar SAM, which could then adhere dispersed CNTs. They found that the surfactant‐dispersed CNT method was more effective at patterning individual CNTs, as the CNTs did not aggregate and form bundles in solution. Line patterns < 40 nm also resulted in too low deposited CNT densities where many patterned lines contained no deposited CNTs. More simply, nanoscale patterns of charge can be written onto substrates by applying voltage pulses through a scanning AFM probe.^[^ ^128^ ^]^ These charge patterns on poly(methyl methacrylate) (PMMA)/Si substrates can electrostatically attract surfactant‐wrapped CNTs from aqueous solutions into nanoscale patterns. This technique of patterning surface charges is called nanoxerography. Charged stamp CP can be used instead of scanning AFM probe to facilitate high‐throughput production,^[^ ^129^ ^]^ however charged stamp CP has not been used to pattern CNTs yet. Nanoxerography may show great potential for patterning of CNTs and is worthwhile exploring to achieve both nanoscale resolution and higher throughput.

Park et al. demonstrate an innovative method where a SWCNT dispersion was blade sheared over a SAM‐patterned substrate to simultaneously shear‐align and nanoscale‐pattern SWCNTs (Figure 5c).^[^ ^110j^ ^]^ Alternating regions of wetting/de‐wetting SAMs were patterned on the substrate and a shearing blade with semiconducting‐enriched SWCNT dispersion was sheared over the whole substrate. SWCNT line patterns with down to 500 nm width were fabricated with alignment along the line length due to solution shearing and self‐alignment for the narrower lines (<0.7 µm) (Figure 5d). SWCNT density was high at ≈150–200 SWCNTs µm^−1^ in the patterned region although maximum monolayer thickness. The aligned SWCNT lines were found to increase on‐current up to 45‐fold compared to random aligned SWCNT lines. Whole wafers were patterned demonstrating the scalability, manufacturability, and potential R2R compatibility of nanoscale patterning using SAM‐patterned substrates. However, speed of patterning was limited in this example to ≈0.4–0.8 mm s^−1^ due to relying on evaporation‐driven flow to densely deposit the SWCNTs. Lee et al. demonstrated a novel nanoscale CNT patterning technique that exploits the faster corrosion rate of a lower reduction potential material when in electrical contact with a higher reduction potential material.^[^ ^130^ ^]^ Nanoscale Ag patterns were fabricated via photolithography on a substrate and a CNT film was deposited over the top of the whole substrate. The substrate was annealed, and the Ag regions selectively corroded the CNT film, leaving nanoscale CNT film patterns remaining on the non‐Ag regions. Noble metals (Au, Ag, Pt) have high reduction potentials and were found to easily oxidize the CNTs at low temperatures when in contact.

Covalent functionalization of CNTs with species containing acid end groups (alkylphosphonic acids,^[^ ^118d^ ^]^ alkylhydroxamic acids^[^ ^2^, ^110^, ^118^ ^]^) allows CNTs to selectively bind to patterned metal oxide surfaces (Al_2_O_3_, HfO_2_) on Si/SiO_2_ substrates. This technique can effectively and precisely pattern CNTs with nanoscale resolution onto important high‐*k* dielectric materials used in CMOS electrical circuits. However, deposition can potentially take hours.^[^ ^2^, ^110^ ^]^ An additional annealing step can restore excellent electrical properties to the CNTs by removing the organic functionalization.^[^ ^110^, ^118^ ^]^ Bardecker et al. applied a photosensitive SAM to a HfO_2_ substrate, and patterned the SAM by converting the SAM from hydrophobic to hydrophilic upon UV‐exposure through a mask.^[^ ^118^, ^131^ ^]^ Aqueous SWCNT dispersions were then deposited on the substrate, depositing the SWCNTs onto the hydrophilic regions due to the hydrophobic regions repelling the dispersion. However, producing nanometer resolution patterns can be timely and costly, due to the limitations of UV photolithography. EBL may instead be used to more easily define nanometer resolution patterns. Schopf et al. spin coated *α*‐functionalized poly(poly(ethylene glycol) acrylate) (polyPEGA) onto a silicon wafer, and exposed the polymer to EBL.^[^ ^120^ ^]^ EB exposure created PEG cross‐links with the silicon oxide surface, adhering the polymer to the substrate surface. Excess polymer was washed away, revealing cross‐linked polymer in desired nanometer resolution patterns on the substrate. CNTs from dispersions were adhered to the polymer patterns, but the deposited CNT density appeared to be low with high line width roughness.

Park et al. with Hannon and Haensch from the IBM Thomas J. Watson Research Center reported a massive improvement to the technique, achieving a very high patterned CNT density of 10^9^ cm^−2^ with 90% yield of at least one CNT in a patterned trench.^[^ ^2b^ ^]^ This density is approaching the minimum 10^10^ cm^−2^ with a <10 nm pitch required for high‐performance logic.^[^ ^132^ ^]^ They could place CNTs into a minimum of 70 nm wide trenches with 200 nm pitch with up to 78% yield of a single CNT in each trench. The key for them to obtain high CNT density was forming a strong electrostatic attraction between the CNT‐dispersing surfactant and the patterned SAM. Negatively charged head groups on the anionic surfactant SDS were attracted through strong Coulombic interactions to the positively charged pyridinium SAM (4‐(*N*‐hydroxycarboxamido)‐1‐methylpyridinium iodide). The SAM also had a hydroxamic acid end group, which can selectively assemble monolayers on HfO_2_ and not SiO_2_. This allowed them to selectively pattern the attractive SAMs on prepatterned HfO_2_/SiO_2_ substrates. Cleanliness of the substrates was important to obtain high patterning yields, and an annealing step could remove the SAM and drastically improve device electrical performance. In 2017, they then reported an improvement again, tuning the chemistry of the SAM on HfO_2_ to strongly bond to the sorting polymer used to produce highly semiconducting‐enriched SWCNT dispersions (Figure 5e).^[^ ^133^ ^]^ This simplifies the process of getting sorted SWCNTs into devices, as the sorting polymer can stay wrapped on the SWCNTs and aid in guiding the SWCNT patterning. They used the copolymer poly((9,9‐dioctylfluorenyl‐2,7‐diyl)‐alt‐*co*‐(6,6′‐(2,2′bipyridine))) (PFO‐BPy) to sort the SWCNTs to obtain a toluene SWCNT dispersion with semiconducting purity of >99.9%.^[^ ^133^ ^]^ Nanoscale trenches of HfO_2_/Al_2_O_3_ on SiO_2_ were patterned by conventional lithography (EBL/photolithography), and a SAM consisting of 11‐(4‐aminophenoxy)‐1undecylhydroxamic acid (AMUHA) was formed over the trench area. The hydroxamic acid group selectively bonded to the HfO_2_ layer regions, and the amino end group was converted in situ to a diazonium salt by addition of amyl nitrite. The pre‐sorted polymer‐wrapped SWCNT dispersion was directly dropped onto the substrate and the pyridyl‐containing polymer formed a charge‐transfer complexation with the diazonium group on the SAM, selectively and strongly adhering the SWCNTs to the patterned HfO_2_ trench regions. Functional SWCNT device yields of ≈90% and 73% were obtained from 100 and 50 nm wide trenches, respectively, with a high density of ≈3−4 tubes/trench for the 50 nm wide trenches (Figure 5f–h). The high yields from hundreds of devices on a single chip demonstrates the uniformity and high density of the technique. They used this CNT pattering technique to fabricate complementary SWCNT ring oscillators using a CMOS compatible process with a stage switching frequency up to 2.82 GHz,^[^ ^78a^ ^]^ proving the potential of this CNT patterning technique to create next‐generation integrated circuits (ICs). Similarly, other groups have used polymer or even DNA‐sorted semiconducting SWNCTs for nanoscale patterning of SWCNT dispersions. Penzo et al. patterned 10 nm wide, 100 nm spaced hydrophilic SiO_2_ patterns on hydrophobic PEG‐coated Si substrate using EBL (Figure 5i,j).^[^ ^126c^ ^]^ They subsequently could deposit single DNA‐wrapped semiconducting‐enriched SWCNTs on the patterns with a remarkable 95% single SWCNT yield. Dispersing the SWCNTs in a buffered solution formed a magnesium ion charge inversion layer which was essential to provide adhesion between the both negatively charged DNA‐wrapped SWCNTs and SiO_2_ hydrophilic surface. Derenskyi et al. used polyfluorene derivatives to sort SWNCTs, and deposited single semiconducting‐enriched SWNCTs as FET devices with 100% yields for the devices they fabricated.^[^ ^134^ ^]^

Substrate modification patterning is a powerful nanoscale CNT patterning technique with great potential for fabricating advanced CNT devices with exceptional patterned CNT density, nanoscale lateral resolution, scalability, and CMOS compatibility. Recent work has achieved important milestones that allow the technique to compete with current commercial state‐of‐the‐art techniques. Individual semiconducting‐enriched SWCNTs patterned with SWCNT density of >10^9^ cm^−2^, pattern region resolution of 50 nm, and ≥95% device yields are now readily achievable. Substrate modification patterning consequently has a particular advantage for fabricating advanced nanoscale CNTFET devices using semiconducting SWCNTs as the channel material. Reliable yields must still be increased drastically though, as very low device failure is required for modern ICs to function effectively and reliably. Increasing yields may potentially be achieved by increasing the time the CNT dispersion remains on the patterned substrate, allowing more CNTs to randomly migrate within range for the short‐range electrostatic attraction to pull and adhere the dispersed CNTs onto the patterned regions. However, more interaction time will clearly decrease the technique's throughput. Future work to increase yield should consider adding extra forces like electric fields/Marangoni flows/etc. to pull dispersed CNTs toward the patterned regions and ensure each region has proximate CNTs that can be attracted to the region within a reasonable time. Throughput of substrate modification patterning is currently generally slow, taking up to hours to pattern a wafer substrate, and most methods are batch processes. However, certain methods like the blade coating with patterned substrate have higher throughput and are potentially R2R compatible, while still achieving nanoscale resolution.^[^ ^110j^ ^]^ Also, conventional lithographic processes of photolithography or EBL are often still required for prepatterning the substrate, limiting the throughput and manufacturability.

### 3.2 Template Patterning

Similar to substrate modification patterning, physical templates may be used to guide the flow of CNTs or CNT dispersion onto specific regions of a substrate. These methods generally employ polymer stamps, molds, or photoresist to direct the flow of CNTs in a variety of ways. Stamps and molds used for this method are also often reusable, simplifying and quickening the process to pattern CNTs in scale, and even allowing the possibility of R2R printing for some methods. As this technique mostly patterns CNTs from dispersions, the pattern quality/lateral resolution and the patterning speed/throughput are dependent on the CNT dispersion properties. Higher concentration CNT dispersions will allow increased density of deposited CNTs in the template patterns or a higher throughput speed. However, high concentrations of CNTs in dispersions are difficult to achieve. Using longer CNTs will generally allow for better conduction/mobility through the CNT patterns, but will also decrease the maximum CNT concentration possible. See Section 2.1 for more details on the dispersion of CNTs and the effects on patterning techniques. Longer CNTs do not have as much impact on pattern lateral resolution for template patterning techniques due to template confinement causing self‐alignment of CNTs (Section 2.4.3).

Yan et al. first demonstrated a template CNT patterning technique that could achieve submicron resolution.^[^ ^135^ ^]^ They fabricated a polydimethylsiloxane (PDMS) mold with ≈850 nm wide, ≈400 nm deep channels etched out of one side, inverted the mold, and placed it onto a Si substrate silanized with APTES amine‐terminated SAM. An aqueous dispersion of hydrophilic acid‐functionalized SWCNTs was placed at the openings of the submicron channels, and capillary forces pulled the SWCNT dispersion through the channels. It was important for the channels to have hydrophilic surfaces to increase the capillary effect of the aqueous dispersion. The SWCNTs were deposited from the dispersion along the substrate in the pattern of the channels, and the shear fluid flow through the channels helped to align the SWCNTs along the channel length. The PDMS mold could then be removed, leaving submicron width SWCNT lines on the Si substrate. Long SWCNTs and higher SWCNT concentration could block the channels and inhibit the flow. They partially alleviated this by introducing wider channel entrances that tapered to the desired width, and by applying a gas flow before and after the capillary process to align the SWCNTs in dispersion.^[^ ^135^, ^136^ ^]^ SWCNTs lines aligned by gas flow could even reach 20 nm widths.^[^ ^136^ ^]^ However, there was a limit on the SWCNT concentration and length that could be used. The group also demonstrated that the patterned SWCNT lines from this technique could be transferrable.^[^ ^137^ ^]^ They instead patterned the SWCNT lines on flat hydrophilized PDMS substrate, and then pressed the patterned flat PDMS onto a hydrophilized Si substrate with prepatterned Au electrodes, efficiently depositing the SWCNT lines on the electrodes. Transfer could be done at ambient temperature in seconds and over rough/uneven surfaces, and SWCNTs were adhered well even through rinsing. There was a balance in SWCNT concentration, with ≈1.0 mg L^−1^ dispersions providing incomplete coverage, and higher concentrations >3.0 mg L^−1^ causing entanglement and much less CNT alignment. Kim et al. were able to push this method to produce nanoscale features, using instead rigiflex polyethylene diacrylate molds.^[^ ^138^ ^]^ They were able to pattern 50 and 200 nm wide SWCNT lines with multilayers, however the evaporation process required hours.

**Figure 6:**
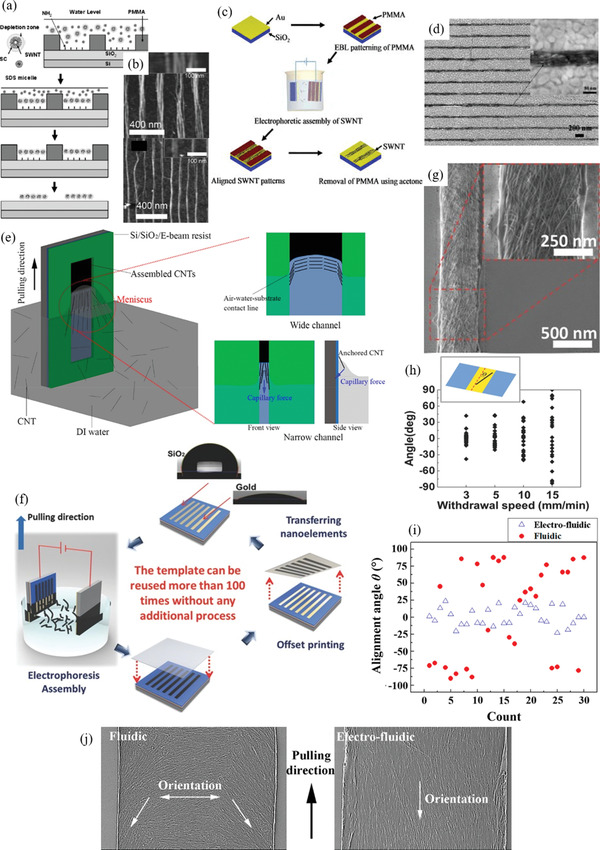
a) Diagram showing the formation of SWCNT line patterns with <200 nm width using PMMA template patterning and depletion forces. b) AFM images of the SWCNT line patterns with 200 nm pitch (top) and 100 nm pitch (bottom). Reproduced with permission.^[^ ^139c^ ^]^ Copyright 2014, Wiley‐VCH. c) Diagram depicting electrophoresis‐aided CNT template patterning and d) SEM image of resulting SWCNT line patterns on Au substrate with ≈80 nm width and ≈200 nm separation. Reproduced with permission.^[^ ^140^ ^]^ Copyright 2007, AIP Publishing. e) Diagram showing the process of microfluidic‐assembly of nanoscale SWCNT patterns on a templated substrate during dip‐coating. SWCNT alignment along contact line occurs with wide trenches and along trench length with narrow trenches. Reproduced with permission.^[^ ^141c^ ^]^ Copyright 2018, American Chemical Society. f) Electrophoresis‐enhanced offset printing of SWCNTs using a reusable Damascene template. g) SEM image of submicron resolution SWCNT line pattern from SWCNT offset printing process. h) Graph comparing SWCNT alignment with line direction and withdrawal speed of damascene template from SWCNT dispersion. Reproduced with permission.^[^ ^142^ ^]^ Copyright 2015, Wiley‐VCH. i) Plot and j) SEM image comparison of SWCNT alignment with and without electrophoresis. Reproduced with permission.^[^ ^141c^ ^]^ Copyright 2018, American Chemical Society.

Often templates can simply block regions on the substrate from CNT deposition, allowing the CNTs to deposit on the unblocked regions.^[^ ^122^, ^139^ ^]^ Templates are made by conventional photolithography, EBL, or nanoimprint lithography (NIL), and they are removed afterward to reveal patterns of deposited CNTs from dispersion. Layer‐by‐layer assembly of cationic/anionic layers of CNTs or polyelectrolytes can be applied to a substrate with a nanoscale patterned template to create CNT patterns with controllable thicknesses.^[^ ^122^, ^139^ ^]^ Additionally, charged SAMs can be applied to the unblocked region to electrostatically adhere the CNTs to the substrate.^[^ ^139b,c,e^ ^]^ The template can be washed away afterward, but appropriate materials should be considered to avoid also removing the CNT layers.^[^ ^122^ ^]^ Attractive depletion forces between CNTs that occur at surfactant concentrations greatly surpassing the critical micelle concentration can aid assemble of CNT patterns into the template patterns (**Figure** **6**a).^[^ ^139c^ ^]^ All these templating methods can produce aligned CNT patterns with <100 nm features and pitch (Figure 6b),^[^ ^139c^ ^]^ however the process can be slow (>1 h) and the patterned areas are not necessarily completely covered with CNTs.^[^ ^139c–e^ ^]^

Researchers with Busnaina and Jung presented a simple technique where a PMMA resist was patterned by EBL over a Au layer, and electrophoretic assembly was applied to selectively deposit SWCNTs onto the exposed Au regions (Figure 6c).^[^ ^140^ ^]^ The PMMA‐patterned substrate was placed in an aqueous SWCNT dispersion, and a bias was applied between the Au layer electrode and another reference electrode in the dispersion. The charged SWCNTs quickly migrate to the substrate, adhering selectively to the Au regions and staying adhered through the PMMA removal step. The electrophoretic assembly allows quick fabrication of concentrated 80 nm wide SWCNT lines in minutes (Figure 6d). Busnaina and Jung continued this effort with a series of papers using nanoscale patterned PMMA templates on hydrophilic substrates to direct the microfluidic assembly of SWCNT lines via dip‐coating method (Figure 6e).^[^ ^30^, ^110^, ^141^ ^]^ Si substrates were treated with dry plasma to greatly increase hydrophilicity by introducing Si—OH and other oxygen groups on the surface. A hydrophobic PMMA template is then applied and patterned via standard lithography methods, creating patterned trenches on the substrate. When the patterned substrate is slowly pulled out vertically from an aqueous SWCNT solution, the evaporating contact line stays high on the hydrophilic regions and low on the hydrophobic regions. The liquid evaporates faster from the hydrophilic regions due to increased liquid surface area, and thus pulls in more liquid from the dispersion to replace it. This liquid flow concentrates the SWCNTs to the hydrophilic regions, and deposits them at the receding contact line. The hydrophilic region and hydrophilic functionalized SWCNTs are also electrostatically attracted. This method allows uniform dense SWCNT lines to be patterned at nanoscale resolution on hydrophilic substrates. For full coverage though, the process must be done in opposite directions to pattern SWCNT at the last end of the channels.^[^ ^110g^ ^]^ The receding contact line and fluid flow help to align the CNTs along the trench lengths. Also thinner trench widths help to self‐align the CNTs, especially when the width is less than the average CNT length (see Section 2.4.3).^[^ ^110g^ ^]^ However, the patterning speed can be slow with substrate pulling speeds of 0.1–0.5 mm min^−1^, as the process relies on evaporation of water to concentrate the SWCNTs at the contact line. The SWCNT lines fabricated using this technique are dense and highly conductive, reaching resistivity of 10^−6^ Ω m due to their dominant metallic behavior.^[^ ^110i^ ^]^ The SWCNT lines can also be easily fabricated with lengths covering whole wafers and with micron widths.^[^ ^110i^ ^]^ They were also able to pattern the SWCNT lines onto a polymer substrate (parylene‐C) by treating it with oxygen plasma to hydrophilize it, and then pattern a hydrophobic photoresist layer on top.^[^ ^141b^ ^]^ The resistance of the SWCNT lines was generally higher at ≈300 Ω, but could remain conductive while bending the substrate. Decorating these SWCNT lines with Pt nanoclusters can decrease the resistivity by increasing the conduction channels available close to the Fermi level, while also allowing higher current densities of ≈10^7^ A cm^−2^ comparable to Cu.^[^ ^110h^ ^]^

Busnaina et al. reintroduced electrophoretic assembly into the technique to increase the deposition speed, so CNTs deposit into the desired patterns within minutes for whole substrates; creating a nanoscale offset printing process (Figure 6f).^[^ ^142^ ^]^ They made a reusable “Damascene” template of metal electrodes in the desired pattern, with an insulating SiO_2_ as the nonpatterned background. They placed the template in an aqueous SWCNT dispersion and then withdrew the template while applying a bias. This allowed CNTs to concentrate quickly via electrophoresis then deposit onto the patterned electrode regions at the receding water contact line as described earlier. Substrate withdrawal speeds of 1–20 mm min^−1^ could be achieved using electrophoresis with possible submicron resolution features (Figure 6g). The CNT patterns were then transferred to polyethylene naphthalate (PEN) substrates by first plasma oxidizing the PEN to make it hydrophilic and increase adhesion forces, and then the template was pressed at high pressure and temperature (above glass transition temperature) into the PEN substrate, engulfing and adhering the CNT patterns to the substrate. CNT alignment along the channel increased when the withdrawal speed was lower (Figure 6h). Under a certain threshold speed, CNTs were always aligned with the withdrawal direction regardless of the feature widths.^[^ ^141c^ ^]^ This was caused by the electric field polarizing the CNTs in dispersion and aligning them with the field (normal to the substrate) (Figure 6i,j). Resistivity of the CNT patterns could reach 4 × 10^−6^ Ω m for 10 µm wide features, and SWCNT cross patterns could achieve 31 Ω sq^−1^ with 90% transparency.^[^ ^142^ ^]^ They later demonstrated a NO_2_ gas sensor made from measuring the resistance through a 300 nm wide CNT channel printed using the technique.^[^ ^143^ ^]^ Lakshmanan et al. similarly used electrophoresis for fast deposition of vertically oriented individual SWCNTs in nanoscale holes through an EBL patterned insulator layer onto a metal substrate.^[^ ^144^ ^]^ The patterning technique was very fast as deposition occurred in seconds. However, SWCNTs longer than the hole depth tended to form networks around the holes due to aggregation.

Template patterning techniques show great potential to obtain nanoscale resolution CNT patterning with high throughput, allowing high performance CNT devices to be fabricated in a commercially viable way. The techniques which incorporate additional electrophoretic assembly are particularly important for increasing throughput,^[^ ^141^, ^142^ ^]^ as it allows a greater flux of CNTs to the patterned substrate than is normally possible with low concentration CNT dispersions. It is a good example of novel methods that can bypass the property trade‐offs in CNT dispersion patterning. Further research into template CNT patterning to make it R2R compatible will increase the manufacturability and allow the technique to be used in mass fabrication of advanced nanoscale CNT devices. Some of the recent methods reported like the “damascene” template method^[^ ^142^ ^]^ could likely be made R2R with engineering solutions, although there are still restrictions on which substrates could be used. Most of the template CNT patterning techniques would require a good transfer technique to allow R2R processing, to transfer the CNT patterns in the templates onto target substrates. A potential R2R‐compatible transfer technique called liquid bridge transfer that could be used with template CNT patterning techniques was reported recently by Corletto and Shapter^[^ ^145^ ^]^ They deposited SWCNTs into <10 µm wide channels on a polymer stamp and then inverted it onto a target hydrophilic substrate with a thin ethanol layer covering the target substrate. As the ethanol evaporates, a liquid bridge forms between the SWCNT patterns in the channels and the target substrate, consequently pulling the SWCNT patterns out of the stamp channels and adhering to the target substrate. A thin release layer of ethanol‐dissolvable Nafion polymer underneath the SWCNT patterns also aided in successful transfer to the target substrate.^[^ ^145^ ^]^ Transfer techniques like liquid bridge transfer that can transfer the template‐patterned CNTs onto target substrates will be critical to further develop and realize R2R nanoscale CNT patterning. Template patterning techniques have a particular advantage in effectively fabricating thicker and more conductive CNT patterns for use as electrodes or interconnects in applications. Reported template patterning techniques currently have mediocre control of the thickness of the CNT patterns, which can be an issue for fabricating semiconducting channels in CNTFETs as detrimental screening effects can dominate for thicker patterns. The coverage and yield of template CNT patterning techniques is also often very high, critical for quality and reliable device manufacturing. However, template patterning is poor at controlling the placement of individual CNTs, and so is better at patterning CNT networks but still with nanoscale resolution.

### 3.3 DEP Patterning

DEP patterning uses DEP to place particles between and across two electrodes with a voltage difference between them. DEP is the phenomenon where a dielectric particle has a force exerted on it when it is placed in an electric field. CNTs exhibit significant dielectrophoretic activity and consequently DEP has been used to pattern CNTs. DEP requires the particles to be suspended in a liquid medium, so that the particles have freedom to rotate and move to the appropriate position. The dielectric particles are attracted to the strongest point of the electric field, which is directly between the electrodes. The electric field tapers off quickly outside the gap between the electrodes. If the gap between the electrodes is small enough, the particles will bridge between the electrodes. The technique is limited though as electrodes are required to be fabricated on the substrate. This adds additional procedures and restrictions to device fabrication. Requiring electrodes also limits the potential throughput speed of fabrication using this technique.

**Figure 7:**
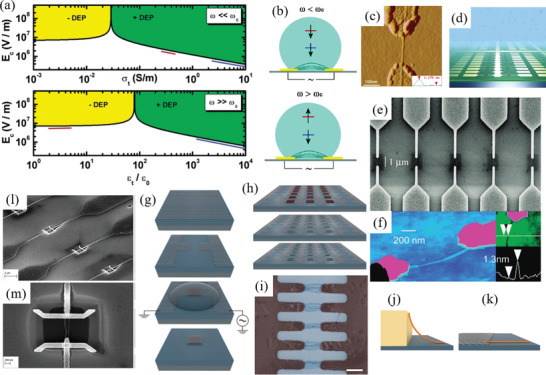
a) Example plots depicting DEP regimes for SWCNTs in 0.1% sodium dodecylbenzenesulfonate surfactant aqueous suspension. For low‐frequency AC DEP (top), conductivity and electric field strength determine whether CNTs are attracted (green, + DEP) or repelled (yellow, – DEP) from the electrodes. For high‐frequency AC DEP (bottom), relative permittivity (dielectric constant) and electric field strength determine attraction (green, + DEP) or repulsion (yellow, – DEP) from the electrodes. b) In this example, values of conductivity/relative permittivity of metallic (blue) and semiconducting (red) SWCNTs mostly result in attraction to the electrodes except for semiconducting SWCNTs in the high‐frequency regime. Note that this plot only represents values for a specific example and different solvents, dispersing agents, and CNTs will change these values. Reproduced with permission.^[^ ^146^ ^]^ Copyright 2004, American Chemical Society. c) AFM image of a single individualized SWCNT deposited across Ag electrodes with inset showing topography over the deposited SWCNT. Scale bar 100 nm. Reproduced with permission.^[^ ^156^ ^]^ Copyright 2003, American Chemical Society. d) Schematic of DEP CNT patterning for fabrication of high‐density CNT device array. e) SEM image and f) AFM images of fabricated single CNT devices. Reproduced with permission.^[^ ^2a^ ^]^ Copyright 2007, American Chemical Society. g) Schematic depicting DEP patterning of CNTs on graphene electrodes and subsequent etching of the graphene. h) The nanoscale DEP patterning technique can pattern different nanomaterials between removable electrodes over whole wafers. i) AFM image of deposited CNTs between graphene electrodes with nanoscale precision. Scale bar 1 µm. j) Curved deposition of CNTs on metal electrodes and k) flat deposition of CNTs on graphene electrodes. Reproduced under the terms of the CC BY 4.0 license.^[^ ^164c^ ^]^ Copyright 2005, The Authors, published by Elsevier. l) Array of nanoscale‐suspended SWCNT devices fabricated via DEP patterning. Scale bar 2 µm. m) Suspended SWCNT device with only two parallel SWCNTs suspended between the electrodes. Scale bar 200 nm. Reproduced with permission.^[^ ^183^ ^]^ Copyright 2015, Wiley‐VCH.

The general common theory for AC DEP patterning of CNTs was defined by Krupke et al.^[^ ^146^ ^]^ and supported by later experiments, simulations, and theory.^[^ ^147^ ^]^ They stated that AC electric field strength and frequency, and the dielectric constant and conductivity of the CNTs and liquid medium, determine whether the CNTs experience positive or negative DEP (**Figure** **7**a,b). The theoretical dielectrophoretic force exerted on the CNTs can be approximately described by the dielectrophoretic force equation for a long rod

(1) $$\begin{array}{c} F_{\mathrm{DEP}}=\frac{\pi d^{2}l}{8}\varepsilon_{s}Re\left(\frac{\varepsilon_{t}^{*}-\varepsilon_{s}^{*}}{\varepsilon_{s}^{*}+\left(\varepsilon_{t}^{*}-\varepsilon_{s}^{*}\right)L}\right)\nabla E^{2} \end{array}$$

(2) $$\begin{array}{c} \varepsilon_{t,s}^{*}=\varepsilon_{t,s}\;-i\frac{\sigma_{t,s}}{\omega } \end{array}$$

where *d* and *l* are the diameter and the length of the CNT, $\varepsilon_{t}^{*}$ and $\varepsilon_{s}^{*}$ are the complex permittivity (dielectric constant) of CNT and solvent, *ε* and *σ* are the real permittivity and conductivity, *E* and *ω* are the electric field strength and angular frequency, and *L* is the depolarization factor. At low frequency (*ω*), conductivity (*σ*) dominates the ε* term, and at high frequency, the real relative permittivity dominates. So for the low‐frequency regime, CNTs with conductivity lower/higher than the solvent experience negative/positive DEP, respectively. Likewise, in the high‐frequency regime, CNTs with permittivity lower/higher than the solvent experience negative/positive DEP, respectively. A certain threshold of electric field strength is also required to initiate DEP. This means that metallic CNTs experience positive DEP, while semiconducting CNTs can sometimes experience negative electrophoresis. However, the solvation shell around the CNTs must also be included when calculating the permittivity and conductivity of the CNTs.^[^ ^147^, ^148^ ^]^ Stern/Ion layer, free surface charges, diffuse double layer, surfactants, and impurities can all contribute to the conductivity/permittivity of the CNTs in suspension, affecting the applied dielectrophoretic force.

DEP patterning is very effective compared to other CNT patterning techniques at placing single individual CNTs at target locations with nanometer accuracy. This is important for applications that require that precise control so the excellent properties of CNTs can be exploited in nanoscale advanced devices, particularly for CNTFETs, sensors, and photonics. However, throughput is still somewhat limited as it requires batch processing, although whole substrate wafers can simultaneously be patterned. The technique requires particular substrates with patterned electrodes which can limit its applications. Recent work though has demonstrated DEP patterning with removable electrodes, which increases the possible range of devices that can be fabricated by removing the restriction on compatible substrate designs. Yield of successfully fabricated devices with currently reported DEP techniques is quite high at >90%.^[^ ^2a^ ^]^ However, to ensure reliable and high‐quality devices, much higher yields of >99% have to be achieved. This may be possible with more investigation into the self‐limiting single CNT/bundle deposition mechanism which could allow 100% yield of devices given enough assembly time (see Section 3.3.4). High CNT densities up to 50 CNT µm^−1^ have recently been reported for DEP techniques, showing the potential for the technique in fabricating very highly scaled, nanoscale devices. Achieving higher CNT densities will likely require higher resolution electrode fabrication, although self‐limiting deposition may potentially be exploited to deposit CNTs with equal and tight pitch along the same electrodes. DEP is an important CNT patterning technique that is already highly effective for manufacturing certain advanced CNT devices and should increase in effectiveness with further research in the field.

#### 3.3.1 Technique Development and Theory

DEP for patterning CNTs was first suggested by Fishbine in 1996,^[^ ^149^ ^]^ and realized experimentally using a DC‐bias by Yamamoto and Nakayama in 1996,^[^ ^150^ ^]^ and then Bezryadin and Dekker in 1997.^[^ ^151^ ^]^ Yamamoto et al. placed a few drops of arc‐discharge CNTs suspended in isopropanol (IPA) between Al electrodes with a 0.4 mm gap, and applied a DC electric field between the electrodes until the IPA had evaporated. They found that the CNTs moved toward the cathode, with average velocity proportional to the electric field, and that aligned CNTs have a higher average velocity. Bezryadin and Dekker were using DEP to obtain electronic transport measurements of single nanoparticles, including CNTs. SWCNTs were suspended in cyclohexane, and a drop of the suspension was applied between two electrodes with a 150 nm gap. ≈30 s after applying a DC bias across the electrodes, a current was detected, indicating that single nanotube bundles were trapped across both electrodes.

Yamamoto et al. in 1998 then used an AC bias instead for DEP alignment of CNTs.^[^ ^152^ ^]^ Again, arc‐discharge MWCNTs were suspended in IPA and dropped onto Al electrodes with a 0.4 mm gap. The frequency was varied between 10 Hz and 10 MHz and they found that higher frequencies resulted in increased tube orientation, and fewer contaminant particles attracted to the cathode. Yamamoto et al. stated that at low frequencies (<10 kHz), the ions surrounding the nanotubes and contaminant particles can move in response to the alternating electric field allowing the charged nanotubes and particles to move preferentially toward the closest electrode due to a larger electric field. There is no discrimination between the nanotubes and particles. However, at higher frequency (>10 kHz) the surrounding ions can no longer move in response to the alternating field, meaning charged particle movement does not occur, and the nanotubes and particles develop an electrical dipole. Nanotubes have a much longer electrical dipole than the particles, and so are preferentially attracted to the nearest electrode. Their theory was also supported by later studies.^[^ ^153^ ^]^ Nagahara et al. used AC DEP placement of aligned SWCNTs across Au electrodes with a 20–80 nm gap.^[^ ^154^ ^]^ A 5 µg mL^−1^ SWCNT Triton‐X 100 surfactant‐stabilized aqueous suspension was dropped onto an electrode array and AC bias of 0.5–2.5 V, 5 MHz was applied for 1–30 s. SWCNT bundles <100 nm diameter were attracted across the electrodes, bridging them together. This technique effectively allowed submicron patterning of CNTs between fabricated electrodes. Similar to Yamamoto et al., they found that using AC bias resulted in fewer contaminants (carbon impurities) co‐depositing on the electrodes with the SWCNTs than DC bias. Nagahara et al. theorized that the cause was that for AC bias, the effect of charge is greatly reduced while the effect of other variables like the dielectric constant of the particle, conductivity, and frequency of the alternating electric field are increased. DC bias will attract charges to an electrode over time, whereas an AC bias has a time‐averaged bias of zero for any electrodes, resulting in AC bias attracting much less charged particles. They showed that at varying AC field frequency, the dielectrophoretic force can be repulsive or attractive, depending on the particle and solution medium complex permittivities (dielectric constants). Frequency can then be used to selectively attract or repel different particles, even in the same solution.

Krupke et al. used AC DEP to selectively deposit single SWCNT bundles between electrode contacts.^[^ ^155^ ^]^ Carboxylic acid‐functionalized SWCNTs were suspended (10 ng mL^−1^) in DMF.^[^ ^155^ ^]^ The SWCNT suspension was dropped onto an Au or Ag electrode array with 80–150 nm wide electrodes with 100 nm pair distance, and an AC bias was applied until the suspension was removed typically after 1 min. Krupke et al. found that SWNCTs were only aligned across electrodes at higher frequencies (>1 kHz). The reason was similar to that theorized by Yamamoto et al. and Nagahara et al. Lower frequencies allows the ions to react to the alternating field, attracting them to the electrodes and creating a Helmholtz double layer at the electrodes and weakening the effective electric field and shielding the dielectrophoretic force applied to the CNTs. Higher frequencies stop this from occurring, allowing the dielectrophoretic force to be applied to the CNTs. Krupke et al. also found that Ag electrodes can control single or few bundle deposition, while Au electrodes resulted in larger numbers of bundles depositing, depending on SWCNT concentration and deposition time. COOH‐functionalized SWCNT bundles without surfactant were attracted to the Ag electrodes and made electrical contact while the suspension drop was still on the electrodes, but could not attach to the Au electrodes and only formed an electric contact when the suspension solvent was removed. This allowed many SWCNT bundles to collect between the Au electrodes before the solvent was removed, resulting in an uncontrolled number of SWCNT bundles between the Au electrodes. They hypothesized that the cause of the self‐limiting deposition was that the deposited CNTs bridging the electrodes reduced the electric field and prevented additional bundles being deposited. However, a later study by Vijayaraghavan et al. found that deposition of a single CNT instead changed the direction of the dielectrophoretic force around the deposited CNT, providing a repelling force instead.^[^ ^2a^ ^]^

Krupke et al. later went on to deposit SWCNT bundles/individual SWCNTs on arrays of multiple submicron Ag electrodes, demonstrating the ability of AC DEP to pattern a large area with multiple electrodes simultaneously (Figure 7c).^[^ ^156^ ^]^ The same SWCNTs suspended in DMF were dropped onto a chip with 16 Ag electrode pairs with 400 nm gap. The SWCNT dispersion was on the chip for 1 min under AC‐bias at 1 V, 1 MHz. At a certain frequency, one SWCNT bundle can be attracted across an electrode pair and then inhibit the attraction of any additional bundles. This results in mostly individual bundles being placed across the electrode pairs (≈70% electrode pairs with single bundle). Krupke et al. claimed that metallic CNTs and bundles containing them are selectively deposited across the electrode pairs, due to having a much higher dielectric constant, which means the positive dielectrophoretic force is much stronger for metallic CNTs. The authors also note that through standard probability, the chance for a metallic CNT to be present in a bundle of seven CNTs (average size for the experiment) is 94%, resulting in limited selectively for CNT bundles. However, in another paper, Krupke et al. demonstrated that indeed the metallic CNTs are selectively deposited during AC DEP over semiconducting CNTs under selected conditions.^[^ ^86^ ^]^ They individualized the SWCNTs using high power ultrasonication and centrifugation, with SDS surfactant in aqueous suspension. Using Raman spectroscopy, they demonstrated individualized metallic SWCNTs were preferentially deposited across the electrodes in AC DEP, enriching the metallic fraction to ≈80%, compared to the natural 33% metallic fraction. Dimaki and Bøggild added to this a numerical study on DEP sorting of CNTs under fluidic flow.^[^ ^157^ ^]^ They state that using this technique on a CNT suspension in fluidic flow, CNTs could be purified to over 99% semiconducting CNTs with high yield.

Krupke et al. published further work effectively showing that AC strength and frequency, and the dielectric constant and conductivity of the CNTs and solvent, determine whether the CNTs experience positive or negative DEP.^[^ ^146^ ^]^ They demonstrated experiments of increasing dielectrophoretic force on semiconducting SWCNTs through increasing the surface conductivity by increasing the concentration of sodium dodecylbenzene sulfonate surfactant that suspended the SWCNTs in D_2_O. Following from Krupke et al., Duchamp et al. investigated the importance of the solvent and substrate properties in DEP of CNTs.^[^ ^147i^ ^]^ MWCNTs dispersed in different solvents were dropped on electrodes with a 2 µm gap and 1 V DC bias and 1 V 1 MHz AC bias was applied for 2 min. They found that the general dielectric constant of the solvent does not necessarily predict the dielectrophoretic effect on CNTs in those solvents, as suggested earlier.^[^ ^146^ ^]^ IPA and cyclohexanone as solvents provided the highest and lowest yields, but with similar dielectric constants of 20.18 and 16.1, respectively, while water had middle yields with high dielectric constant of 78.36. They reasoned that the solvation shells provided the majority of the dielectric permittivity, as both water and IPA solvation shells around CNTs have high permittivity, while cyclohexanone's solvation shell has low permittivity. IPA adsorbed to CNTs still has a freely rotating OH functional group which can provide a large dipole moment. They suggested that when calculating dielectrophoretic force on CNTs, the solvation shell complex permittivity contribution must be added to the CNT complex permittivity.

#### 3.3.2 Deposited CNT Selectivity

The theoretical understanding can be used to engineer CNT dispersions and set‐ups to control the separation of metallic and semiconducting CNTs and their patterned deposition to particular areas on substrates. Some groups have modified the conductivity of the CNTs with functionalization or surfactants to modify the dielectrophoretic force applied to the CNTs.^[^ ^147a,g,147h^ ^]^ Burg et al. used the frequency dependence of the dielectrophoretic force to selectively deposit individual metallic SWCNTs with DEP using a high 200 MHz AC frequency.^[^ ^147k^ ^]^ Vijayaraghavan et al. were able to selectively assemble single‐chirality semiconducting SWCNTs into high‐density arrays with DEP, by using chirality‐selective polymers to selectively suspend CNTs according to their chirality.^[^ ^158^ ^]^ Li et al. introduced light‐assisted DEP to enhance the dielectrophoretic force on semiconducting SWCNTs.^[^ ^159^ ^]^ Semiconducting CNTs experience enhanced polarizability when irradiated with photons by triggering exciton dissociation in the CNTs, and this enhanced polarizability can increase the dielectrophoretic effect. CNT chiralities with stronger absorption at particular wavelengths will experience increased polarization when irradiated by that wavelength, and consequently experience increased DEP. Li et al. prepared separated (6,5) semiconducting SWCNTs in aqueous solution and irradiated a droplet of the solution on electrodes with a 532 nm laser at ≈10^6^ W m^−2^ power density while applying a 1 MHz AC bias across the electrodes. They found that the deposition of the SWCNTs was higher under irradiation than the control, and transistor devices created with the technique had a vastly improved *I*_on_/*I*_off._

#### 3.3.3 Electrothermal Flow

It is important to consider electrothermal flow during CNT DEP patterning. This flow originates from the electric field acting on conductivity/permittivity gradients over the liquid medium caused by nonuniform temperature in the solution. The nonuniform temperature occurs from Joule heating of a conductive solution, which is stronger in stronger electric fields. Calculations have shown that electrothermal flows are responsible for initially transporting the CNTs in aqueous surfactant suspension to the electrodes’ vicinity before the weaker dielectrophoretic force takes effect in the local area to trap the CNTs between the electrodes.^[^ ^147^, ^160^ ^]^ To deposit CNTs, the dielectrophoretic force needs to be strong enough to pull the CNTs from this flow across the electrode gap. The electrothermal flow is directed toward the electrode ends from above (flowing anti‐normal to the substrate), then pushes outward away from the gap along the substrate, and then circulates back over the gap again.^[^ ^147^, ^161^ ^]^ Increased voltage and increased solution conductivity (from surfactant concentration or solvent) can increase electrothermal flow.^[^ ^147^, ^161^ ^]^ A threshold electric field is required to initiate the electrothermal flow, which creates a minimum threshold voltage (and minimum frequency for capacitively coupled electrodes) for DEP deposition of CNTs, in agreement with experiments.^[^ ^146^, ^147^, ^162^ ^]^ High AC frequencies can reverse the electrothermal flow direction,^[^ ^147^, ^160^ ^]^ with lower conductivity solutions having a lower crossover frequency.^[^ ^163^ ^]^ Electrothermal flows in the solution are also strongest around sharper electrodes.^[^ ^161^ ^]^ It has been suggested that electrothermal flows may contribute strongly to the ability of semiconducting CNTs in surfactant suspensions to still deposit with DEP, as the electrothermal flows will carry any suspended particles.^[^ ^147l^ ^]^

#### 3.3.4 Deposition Rate, Density, and Distribution

The factors enabling control of CNTs deposited during DEP have been explored by many groups with the aim of enabling precise patterning of singular CNTs bridging electrode pairs. Generally, the density of CNT bundles deposited between electrodes can be controlled by varying the electric field strength.^[^ ^147^, ^161^, ^162^, ^164^ ^]^ An increased electric field will increase the acceleration of the CNTs due to dielectrophoretic and electrothermal forces, potentially allowing more CNTs to collect in a certain time period. CNT suspension concentration will affect CNT deposition rate, with higher concentration resulting in CNT bundling and increased deposition rate.^[^ ^164^, ^165^ ^]^ Clearly, increased deposition time will also increase the amount of deposited CNTs,^[^ ^166^ ^]^ where deposition time is the period when bias is applied between electrodes while covered in CNT suspension. Also, an increased solution viscosity will slow the rate of CNT deposition due to decreased CNT velocity through the solution medium.^[^ ^147m^ ^]^

A combination of both AC and DC components for DEP placement of CNTs was employed by Chung et al. to control CNT deposition rate.^[^ ^162a^ ^]^ Individualized MWCNTs in ethanol were dropped on pairs of Al electrodes with a 4 µm gap, and an electric field with AC and DC components was applied until the solvent evaporated. The addition of the DC field was used to control the deposition of only singular CNTs, achieving an ≈90% yield of single CNT deposition across electrode gaps. They stated that the addition of a small amount of DC electric field creates electroosmotic flow between the electrodes. The deposition of the initial CNT disturbs this flow to create vortexes around the initial first deposited CNT, even with imperfect electrical contact to the electrodes. These vortexes prevent additional CNTs from depositing near to the initial deposited CNT, allowing isolated CNTs to be deposited with DEP using a variety of electrodes. Using a square wave AC may also be used in place of adding an additional DC component.^[^ ^167^ ^]^ Another technique to control single tube deposition is attaching an external limiting resistor in series with the circuit.^[^ ^155^, ^162^, ^168^ ^]^ A large voltage drop occurs when a singular CNT bridges the electrodes with a large resistor connected in series, as the largest voltage occurs at the point of the largest resistance. This effectively reduces the electric field and consequently the dielectrophoretic force after single CNT deposition, creating a self‐limiting process. A high enough electric field can dominate any self‐limiting process, however.^[^ ^147^, ^164^ ^]^

Vijayaraghavan et al. demonstrated in 2007 that DEP can be used to fabricate a large scalable amount and high density of single CNT devices on single chips (Figure 7d,e).^[^ ^2a^ ^]^ They achieved a CNT device density of >10^6^ cm^−2^ (putting it within the realm of the ultra‐large‐scale integration (ULSI) for microelectronics) with a 90% yield of electrode pairs with a single deposited CNT (Figure 7f). 10 ng mL^−1^ individualized SWNCTs suspended with surfactants was dropped onto silicon substrates with thermal oxide layer and Pd/Ti electrodes with 0.8 µm gap. The high density and yield of devices were made possible by using a floating counter electrode for one of the electrodes. An AC bias of 2 V, 300 kHz was applied between the main electrode and a silicon gate electrode, which forms the substrate. The floating counter electrode is then capacitively coupled to the silicon gate electrode, acquiring a similar potential, while not requiring direct wiring. They also investigated and discussed the effective self‐limiting single CNT/bundle deposition mechanism, which inhibits additional CNT/bundles depositing. They found using impedance spectroscopy and finite element partial differential equations solver (FlexPDE) simulations, the total potential difference across electrodes is not significantly reduced after a single CNT/bundle is deposited (without an external resistor), as proposed previously.^[^ ^155^ ^]^ Instead, deposition of a single CNT changed the direction of the dielectrophoretic force around the deposited CNT, providing a repelling force. If the CNT suspension is diluted enough for only one CNT entering the attractive electrode gap region (≈5 nanotubes µm^−3^), then only single CNTs will be deposited. Later studies are consistent with this analysis.^[^ ^164^, ^169^ ^]^

Davis et al. added to this by demonstrating that a deposited CNT actually repelled in a certain radius, and that if the electrode end was wide enough, multiple CNTs could still deposit.^[^ ^164b^ ^]^ Under the same conditions, an average of 7.7 CNTs were deposited between 1 µm wide electrodes and 2.8 CNTs between 0.1 µm wide electrodes (both with the same electrode gap distance), showing that repelling CNTs can still be placed on the same electrode given enough space. Davis et al. also demonstrated massively parallel CNT device assembly with ≈10^6^ cm^−2^ device density.^[^ ^164b^ ^]^ They were able to create the devices on a nonconducting substrate by using floating electrode pairs capacitively coupled to a larger driving electrode. The floating electrodes area could then be made smaller than ≈10 µm^2^, which is required by floating electrodes capacitively coupled to a conductive substrate.^[^ ^2a^ ^]^ Monica et al. implemented wafer‐scale assembly of CNTs using DEP, except creating thicker networks instead.^[^ ^170^ ^]^ They used 10 µg mL^−1^ CNT suspensions, applying a 24 V 100 kHz AC bias to 432 Au electrode pairs over 5 s to 15 min. As expected, longer deposition times resulted in higher deposited CNT concentration on the electrodes. They also found that increased deposited CNT concentration resulted in increased unaligned deposition due to the electric field being disturbed by previously deposited CNTs. Naieni et al. demonstrated that for surfactant‐free solutions, the combination of electrothermal flow and DEP from a CNT bridged across flat electrodes will attract CNTs in the near range (<100 nm) and slightly repel CNTs in the far range (≈500 nm).^[^ ^160b^ ^]^ This can result in periodic stripes of DEP‐deposited CNTs between wide flat electrodes, with stripe period proportional to electrode gap. This is similar to earlier work by Diehl et al.^[^ ^171^ ^]^ where they reported the self‐assembly of SWCNT stripes that assembled between two electrodes during AC DEP. The periodic spacing between the assembled SWCNT stripes was approximately equal to the SWCNT stripe length. Diehl et al. suggested that the mutual repulsion of the screened‐Coulomb potential between SWCNT stripes positively charged with *ortho*‐dichlorobenzene is the cause of the periodic SWCNT stripe assemblies. Charge screening through additional charge/electrolytes in the solution can therefore dampen the separating, repulsive effect. Both the screened Coulomb potential and the electrothermal force generated by deposited bridging CNTs should be considered when analyzing periodic stripe patterns in AC DEP patterning.

#### 3.3.5 Deposition Process

The sequence of events involved in CNT deposition during DEP is not necessarily the same for all CNT solutions. Some earlier studies found that the rotation of the CNTs during DEP occurs much faster than the translation to the electrodes, ensuring that CNTs are well‐aligned before their deposition onto the electrodes.^[^ ^147b,f^ ^]^ However, later modeling by Oliva‐Avilés et al.^[^ ^148^ ^]^ suggested that solutions with both high aspect ratio CNTs (≥ 1000) and high CNT weight fractions (≥ 7 × 10^−4^) experience CNT‐to‐CNT contact and CNT‐to‐electrode contact concurrently before CNT rotation. The model also predicted that increased AC strength and frequency can increase the rate of CNT alignment, consistent with earlier experiments.^[^ ^172^ ^]^ Including the effects on conductivity and permittivity of an interface layer (Stern layer, free surface charges, diffuse double layer) between CNTs and water in the model was critical to obtaining the refined model. Berger et al. used the more accurate 3D rather than simply 2D electrostatic finite element analysis to simulate the deposition of CNTs to electrodes during DEP.^[^ ^173^ ^]^ Their simulations support earlier studies showing that CNTs generally align quickly before translation to the electrodes. They also found that only CNTs located directly above the electrode gap will initially contact the electrodes bridged, whereas other CNTs will initially contact one of the electrodes end‐first, almost normal to the electrode surface in line with the electric field lines. Only after initial contact, the CNTs can be swept over to the electrode gap. 3D finite element analysis simulates a slower deposition time than 2D due to lower predicted electric fields. The suggested reason was the 3D simulation considers the width of the electrodes, which can contribute significantly to the total electric field magnitude. CNTs, particularly SWCNTs, were also simulated to bend significantly during DEP.

Monica et al. reported that during DEP patterning, CNTs were preferentially attracted to the tip of the longest CNT chain until it bridges the electrodes. They claimed that longer chains have a greater collective dipole moment, therefore have stronger electric field gradients. Naieni et al. built on this by demonstrating with simulation and experiment that CNTs are preferentially attracted to already deposited not fully bridging CNTs forming chains, due to the enhanced electric field at CNT tips.^[^ ^160b^ ^]^ Oliva‐Avilés et al. reported additional experiments and simulations investigating the process of CNT chaining in dispersion during DEP.^[^ ^174^ ^]^ They found that far from the electrodes in the uniform electric field, CNTs can form end‐to‐end chains in the dispersion due to their mutual attraction of induced dipoles. If the CNT concentration is high enough, CNT chaining in dispersion can occur before CNT deposition. However, within a few µm of the electrodes the electric field is strong. CNTs are first attracted to the electrodes before other CNTs can chain to the end of the already deposited CNTs. The threshold distance from the electrode between electrode‐initiated and inter‐CNT‐initiated CNT chains depends on the conditions of the experiment but is generally in the 1–10 µm range. Regardless, long CNT chains can self‐assemble during DEP, which may allow bridging of large electrode gaps that are longer than the CNTs. This chaining process is consistent with previous studies showing similar chaining behavior.^[^ ^160^, ^170^, ^175^ ^]^

#### 3.3.6 Electrode Effects

Xu et al. performed calculations and simulations, along with complementary experiments to investigate the effect of electrode geometries on DEP deposition of CNTs.^[^ ^176^ ^]^ They calculated the dielectrophoretic force, torque, fluid viscous forces applied to a CNT and determined its rotational and translation motions during DEP for a variety of electrode geometries in ethanol. They found regardless of the size or position of the electrodes, the dielectrophoretic force was at a maximum at any corners of the electrodes, and consequently CNT finally deposited and bridged any corners of the electrodes. If the electrode end was fully rounded, then the most protruding point had the maximum force. They confirmed this experimentally by dropping MWCNT suspended in ethanol on Au electrodes. They also performed simulations and experiments demonstrating that electrode spacing to electrode width should be at least 2:1 or higher. Electrodes too close together have overlapping/interfering electric fields, resulting in incorrect CNT deposition. Electrode widths of 3 µm or less are required for single CNT deposition.^[^ ^177^ ^]^ Banerjee et al. also experimented with different electrode architectures, including floating electrode posts and crossed junctions.^[^ ^168^ ^]^ They demonstrated that sub‐micron floating electrode posts can guide the CNTs to deposit between the posts, allowing complex patterns to be formed. A combination of lower voltage and pointed electrodes also allowed them to fabricate crossed nanotube junctions with DEP. Zhang et al. noted that electrode–CNT interaction energy determines how well the deposited CNTs adhere to the electrodes.^[^ ^178^ ^]^ Electrodes with higher interaction energy have stronger adhesion, which can prevent loss of deposited CNTs during subsequent processing steps.

A recent big advance in DEP patterning of CNTs was reported by Engel et al. in 2018 where they demonstrated using graphene instead of metal electrodes.^[^ ^164c^ ^]^ The graphene electrodes can be easily removed via an oxygen plasma etch after DEP deposition, unlike standard metal electrodes which are difficult to remove without displacing the CNTs (Figure 7g). This allows for large‐scale, nanoscale‐precise, simultaneous patterning of CNTs (and other nanomaterials) without requiring permanent electrodes in the device (Figure 7h). They obtained a deposited CNT density between the electrode pairs of ≈15 CNT µm^−1^ average and over 50 CNT µm^−1^ maximum, with alignment 90° ± 10° compared to electrode edge, and no misplaced CNTs (Figure 7i). A normal AC bias of 1–10 V, 0.1–1 MHz for 1–10 min was required, as the graphene electrodes had adequate conductivity. CNTFETs were fabricated using the technique yielding devices with *I*_on_/*I*_off_ of 10^5^. An important benefit of graphene electrodes is their sub‐nanometer thickness, allowing CNTs to be deposited flat and accurately onto the substrate (Figure 7j,k).

#### 3.3.7 Substrate Effects

Vijayaraghavan et al. and Duchamp et al. demonstrated that conductive substrates can interfere with DEP patterning, and so insulating substrates or thick insulating layers should be used for accurate deposition between electrodes.^[^ ^2^, ^147^ ^]^ Semiconducting CNTs can have transverse polarizability that is comparable to the longitudinal polarizability. The transverse polarizability can begin to dominate in DEP deposition of semiconducting CNTs when very high electric fields are used, resulting in unaligned semiconducting CNT deposition^[^ ^179^ ^]^ or even perpendicular alignment.^[^ ^180^ ^]^ Padmaraj et al. exploited the very strong curved electric field of an electrode near a conducting substrate, and consequently were able to deposit metallic SWCNTs parallel and semiconducting SWCNTs orthogonally on the same electrodes simultaneously.^[^ ^180^ ^]^ A suspended structure can be advantageous as it removes negative electric and physical effects of the substrate on the CNT device. Schuerle et al. assembled CNTs suspended over deep trenches between electrode pairs by etching the supporting silicon dioxide layer underneath the DEP‐deposited CNTs.^[^ ^181^ ^]^ CNTs suspended between electrodes over deep trenches allow the devices to be TEM compatible to observe metal–CNT contact strength. Lee et al. instead used a PMMA underlayer to support the CNT.^[^ ^182^ ^]^ Using PMMA avoids the need to use strong acids to etch the underlayer, which may damage/modify the CNTs. Oikonomou et al. were able to produce suspended SWCNT structures between electrodes with no slack/sagging using DEP (Figure 7l).^[^ ^183^ ^]^ The key to their straight rigid structure was having the electrodes and supporting SiO_2_ layer at the same level during DEP deposition to ensure flat deposition. The SWCNTs were then clamped with additional electrode material on top before removal of the supporting layer and suspension of the structure. Devices with only two individualized suspended SWCNTs were able to be fabricated (Figure 7m). Zheng et al. were able to deposit CNTs with DEP on a flexible polyimide thin film.^[^ ^169^ ^]^ This allowed them to flex the substrate during DEP deposition, which then they could relax afterward to straighten the bridging CNTs.

#### 3.3.8 Contaminant Control

Wakaya et al. found that short evaporation times for the solvent and lower voltages resulted in less contaminants being deposited on the electrodes, due to CNTs’ higher mobility.^[^ ^184^ ^]^ They used DC‐electrophoresis to pattern CNTs dispersed in dichloromethane or IPA on Au/Ti electrodes with a 3 µm gap. Dong et al. found that using a control electrode with a much stronger electric field could be used to first collect the impurities and larger CNT bundles, allowing weaker electric field electrodes to then only trap individualized CNTs without impurities.^[^ ^185^ ^]^ Burg et al. simultaneously fabricated 223 devices on a single substrate with DEP, with ultrapure SWCNT individually suspended in surfactant aqueous solution, with no purification steps required.^[^ ^186^ ^]^ The key was to grow SWCNTs with Fe catalyst suspended on silica, which could be easily released in aqueous surfactant suspension without impurities, as the catalyst remains attached to the silica.

### 3.4 Self‐Assembly

Self‐assembly occurs due to a variety of interacting forces. Controlling these forces can produce complex and nanoscale CNT patterns that are useful for many applications. Generally, these self‐assembled patterns are natural, periodic, homogenous patterns, rather than arbitrarily designed. This is very effective for producing these patterns in bulk but prohibits the fabrication of more detailed or complex architectures that are required for many advanced CNT devices. Although the chemistry and physics can be complex, the methods involved are generally simple and efficient, simplifying the patterning process relative to other CNT patterning techniques. The coverage and density of self‐assembly techniques are also generally high.

**Figure 8:**
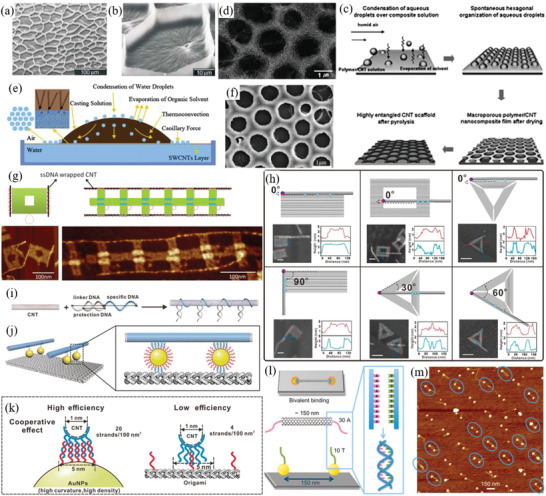
a) SEM image of capillary force self‐assembled CNT “honeycomb” architectures (scale bar 100 µm) and b) an enlarged image of a single “honeycomb” cell (scale bar 10 µm). Reproduced with permission.^[^ ^187^ ^]^ Copyright 2004, Wiley‐VCH. c) Self‐assembly of hexagonally ordered water droplets floating on CNT/benzene solution formed from condensed humid air, followed by drying and pyrolysis to form d) self‐assembled CNT patterns (SEM image, scale bar 1 µm). Adapted with permission.^[^ ^191^ ^]^ Copyright 2009, The Royal Society of Chemistry. e) Self‐assembly of hexagonally ordered water droplets floating on CNT/surfactant/CS_2_ solution due to solvent evaporation‐driving evaporative cooling. Water droplets acted as a mask to allow self‐assembly of CNT patterns f) SEM image of the resulting CNT network patterns (scale bar 1 µm). Reproduced with permission.^[^ ^193^ ^]^ Copyright 2019, Elsevier. g) DNA origami templates with ssDNA binding ssDNA‐wrapped SWCNTs, with fixed ≈100 nm spacing between parallel SWCNTs and bound SWCNTs over 500 nm long. Bottom images are AFM images (scale bars 100 nm). Reproduced with permission.^[^ ^200^ ^]^ Copyright 2013, American Chemical Society. h) Schematics and inset AFM images demonstrating DNA end‐functionalized SWCNTs precisely self‐assembled on DNA origami templates (scale bars 100 µm). End location and orientation can be precisely controlled. Reproduced with permission.^[^ ^202^ ^]^ Copyright 2019, American Chemical Society. i) Functionalization of CNTs with ssDNA that has one end for CNT adhesion and one end for target adhesion. j) DNA‐modified Au nanoparticles can operate as intermediate links between DNA‐CNTs and DNA origami. k) The DNA‐modified Au nanoparticles increase hybridization efficiency due to a higher surface density of ssDNA “hooks.” Reproduced with permission.^[^ ^203^ ^]^ Copyright 2020, Wiley‐VCH. l) ssDNA end‐functionalized SWCNTs can be anchored onto specific substrate locations, including specific locations for both ends (bivalent binding), m) AFM image of anchored DNA‐ended SWCNTs on a nanoscale patterned substrate. Bivalent binding is highlighted with blue ovals. Reproduced with permission.^[^ ^126b^ ^]^ Copyright 2015, American Chemical Society.

Capillary forces can be used to easily self‐assemble CNT films into advanced 2D and 3D architectures with minimal manual manipulation. CVD‐grown CNT forests can be directed and aligned after the CVD growth process, broadening the possible architectures that could be fabricated. Generally, vertically aligned CNT forests anchored to substrates can be densified and/or folded horizontally via capillary forces from liquids surrounding the CNTs. Liu et al. introduced a technique where water was placed onto CVD‐grown vertically aligned CNT films, and the capillary forces from the drying water pulled the CNTs together and bent and pulled the CNTs toward the hydrophilic substrate.^[^ ^187^ ^]^ They calculated that the capillary force exerted on just a single 19 µm long CNT could force up to 12 CNTs to be bent over toward the substrate, until the repulsive van der Waals repulsive force increases at higher densities and balances the net forces. Vertical wall “honeycomb” architectures could be made by laser etching dots in periodic arrays (**Figure** **8**a,b). Vertical walls formed mid‐way between etched dots, where two waves of collapsing CNTs met and pushed on each other. Dense CNTs walls were sub‐micrometer thick, with the “honeycomb” cells between 30 and 150 µm diameter. Although sub‐micrometer thick walls of CNTs could be produced, prior laser patterning of the CNT films was required to produce these complex patterns. Hayamizu et al. presented a technique of densifying and folding patterned SWCNT forests on wafers to assemble dense 3D architectures that could be processed into a variety of microelectromechanical devices.^[^ ^188^ ^]^ They drew the as‐grown vertically aligned SWCNT forest films through an IPA solution, and the capillary forces of the drying solvent densified the SWCNT forests, adhering them together with near‐ideal graphitic spacing. The SWCNT forests were pulled horizontally across and adhered to the wafer. Photolithography and oxidation etching could then be used to engineer them into useful nano/microscale devices on a massively parallel scale across whole wafers. The capillary force‐assisted self‐assembly of CNT honeycomb structures was used as the top p‐type layer and transparent conducting electrode in CNT/Si heterojunction solar cells by Cui et al.^[^ ^189^ ^]^ They instead used hot water vapor to initiate the process, and no laser etched dots were used to position the center of the honeycomb cells. The cell walls of the honeycomb structure operated as highly conductive pathways for the charge carriers to flow, while the rest of the flattened film operated as the p‐layer. After treatment, a sheet resistance down to 10^2^ Ω sq^−1^ could be achieved, giving the solar cell an excellent fill factor of 73%.

An interesting self ‐assembly method involves guiding SWCNTs into porous 2D networks via self‐assembled microsphere monolayers. Dionigi et al. coated SWCNTs onto monodisperse polystyrene microspheres in aqueous dispersions, and allowed these microspheres to self‐assemble into packed hexagonal patterns on silicon substrates.^[^ ^190^ ^]^ After drying, the microspheres were dissolved with organic solvent, leaving behind SWCNT networks with periodic sub‐micrometer “microcapsule” structures.

An all‐liquid self‐assembly technique was demonstrated first by Lee et al. (Figure 8c).^[^ ^191^ ^]^ A dispersion of functionalized MWCNT and amine‐terminated polymer in benzene was uniformly deposited over a substrate under a humid air stream. The evaporating benzene caused cooling of the humid air (evaporative cooling), condensing the humidity into water droplets floating on the benzene solution. These stable floating droplets self‐assembled on the surface into a hexagonal packing pattern, and upon full evaporation of the benzene and water, revealed porous CNT/polymer networks that had precipitated from the benzene. The networks were pyrolyzed to reveal an MWCNT porous network with down to ≈1 µm diameter pores and 4.3 × 10^3^ Ω sq^−1^ sheet resistance (Figure 8d). SWCNT honeycomb structures can be self‐assembled with this technique onto hydrophilic plastic substrates by simply depositing SWCNT/lipid conjugates dispersed in organic solvents at high relative humidity.^[^ ^192^ ^]^ Similar to before, water droplets from the highly humid air condense onto the hydrophilic substrate, spontaneously forming micrometer water droplets. These water droplets act as a mask when depositing the SWNCT/lipid dispersion, allowing SWCNTs to deposit in a honeycomb network pattern. After treatment to remove the conjugating lipids, the SWCNT networks on PET substrate could achieve 3.2 × 10^2^ Ω sq^−1^ sheet resistance, although the network structures had micrometer‐sized features. This method has recently been improved by Dong and Hao, who used a CS_2_ solvent to achieve a much more monodisperse and uniform pore size in the CNT network (Figure 8e,f).^[^ ^193^ ^]^ They suggested the network uniformity was caused by the more efficient evaporative cooling of the more volatile CS_2_ solvent. An ion‐exchange treatment was then used to remove the functionalization on the CNTs and improve their conductivity. Average pore sizes of ≈1.1 µm were created with < 1 µm wide network connections.

### 3.5 DNA‐Assisted Assembly

DNA is a useful dispersing agent for CNTs as unique DNA sequences allows for highly selective adhesion of dispersed CNTs to specific locations on substrates or other DNA structures. The technique has shown great promise for highly precise self‐assembly into different patterns and alignments on devices. A major drawback is the long assembly time (hours or days) to obtain sufficient yields of selectively adhered CNTs. Still, the precision and selectivity of the technique has prompted research into the area that may lead to more advanced techniques with increased manufacturability and precision.

The ability to selectively attach CNTs to specific locations on long DNA strands was demonstrated by Williams et al.^[^ ^194^ ^]^ They covalently functionalized SWCNTs with peptide nucleic acid that had a specific 12‐base‐pair sequence. They could then hybridize these with sections of DNA that had single strands of a complementary sequence, selectively adhering the SWNCTs to the desired section of DNA based on the specific sequence. In this way, SWCNTs of a specific type can be deposited at specific locations with DNA containing complementary sequences. Keren et al. were able to adsorb an SWCNT at a specific location along a DNA scaffold by precise DNA coding and antibody linkages.^[^ ^195^ ^]^ First, short single‐strand DNA (ssDNA) fragments (500 base long) were synthesized with specific coding that matched the desired location on a long double‐stranded DNA scaffold. Then a bacterial protein (RecA) was polymerized onto the ssDNA, which itself hybridized (through a homologous recombination reaction) to the long DNA scaffold at the specific location according to the specific coding sequence. This localized RecA protein could then bind a protein‐functionalized SWCNT to the specific location on the DNA scaffold by using a double antibody linkage. The RecA protein also acted as a resist against metallization. This method allowed them to fabricate single aligned CNT transistors on the DNA scaffold, however the functional device yield was only ≈30%. The DNA scaffolds themselves were also difficult to pattern effectively. Xin and Woolley more simply functionalized DNA strands with aromatic pyrenyl groups that could adhere acid‐purified SWCNT through *π* conjugation.^[^ ^196^ ^]^ SWCNTs were adhered randomly along the functionalized DNA strands that were roughly aligned on a Si substrate, localizing them to the DNA but not at a specific substrate location. SWCNTs can also be functionalized with DNA cross‐linkers of specific length to self‐assemble parallel arrays of SWCNTs with controlled spacing matching the linker length.^[^ ^197^ ^]^

This technique was extended by Maune et al., as they could attach specific SWCNTs to specific locations and orientations on a large 2D DNA template, forming 2D CNT shapes on the DNA template.^[^ ^198^ ^]^ DNA “origami templates” were made of a rectangular DNA template sheet with ssDNA “hooks” in patterns extending perpendicular from the template sheet. The ssDNA hooks had specific sequences matching target SWCNTs. SWCNTs were noncovalently functionalized with ssDNA of the specific sequences, and they could be hybridized in solution to the ssDNA “hooks” at specific locations and orientations on the DNA template. This method allowed them to make different SWCNTs shapes and junctions on the DNA templates. The deposition location of the DNA template on a substrate was not controlled however. FET devices were fabricated using the SWCNT cross‐junctions, but device properties were poor, due to difficulties with DNA template interference when creating and testing the devices. Eskelinen et al. reported a similar technique of using a DNA origami template except using biotin/streptavidin as the connecting “hooks” on the template, slightly simplifying the method.^[^ ^199^ ^]^ Single SWCNT attachment yields of up to ≈78% could be obtained, but incubation times of 1–7 days were required to attach the SWCNTs to the DNA templates. Mangalum et al. used longer DNA origami templates with ssDNA sequences on the edges of the template.^[^ ^200^ ^]^ 500 nm long SWCNTs could be bound to these template edges to hold SWCNT pairs in a parallel alignment with fixed ≈100 nm spacing based on the DNA template (Figure 8g). Controlling the length of the SWCNTs can also be employed to form more precise patterns on the DNA template.^[^ ^201^ ^]^ Pei et al. used SWCNTs that had only the end functionalized with the specific ssDNA, which allowed SWCNT end placement on DNA origami templates to be controlled, rather than just angle and line placement.^[^ ^202^ ^]^ This additional control allowed them to fabricate more complex shapes on DNA templates (Figure 8h) Recently, Zhang et al. used DNA‐modified Au nanoparticles to increase the hybridization/reaction efficiency of the DNA‐CNTs by five times.^[^ ^203^ ^]^ Au nanoparticles have a higher surface density of ssDNA “hooks” on their surface than DNA origami templates, which results in more adhesion events with the DNA‐CNTs (Figure 8i–k).

Crossed, perpendicular junctions of CNTs can be used as Schottky barrier rectifying devices at nanometer size.^[^ ^204^ ^]^ Consequently, SWCNT junctions constructed on these DNA origami templates can be used to create innovative nanoscale electrical circuits and devices. Methods and design tools for using CNT electrical circuits self‐assembled from DNA templates have been investigated and presented, as there are big differences between it and conventional silicon CMOS fabrication.^[^ ^205^ ^]^ Design frameworks and methodology for the modular fabrication of full CMOS‐based nanoelectronic circuits have been investigated through simulation and presented as a guide for future fabrication.^[^ ^205e^ ^]^ However, these assume precise placement of the self‐assembled CNT DNA origami templates of different types onto device substrates. Although SWCNT attachment to DNA origami templates can be quite well controlled down to nanometer resolution, the placement of the DNA templates on device substrates must still be investigated further. Also, the yield and incubation/reaction speed of the CNTs adhering to the DNA templates must be increased quite significantly to be useful for manufacturing, and there is currently minimal work in increasing the yields and speed.

Specific substrate location patterning using DNA was first investigated by Hazani et al., by forming a SAM of thiol‐terminated ssDNA adsorbed onto Au electrodes.^[^ ^206^ ^]^ SWCNTs were functionalized with ssDNA of a complementary sequence, and they were dropped in a dispersion onto the electrodes. The complimentary sequences were then allowed to hybridize, selectively adhering the SWCNTs to the electrodes and bridging some electrode pairs to form electrical contact. However, this technique had poor yield (≈12%), and the DNA hybridization process could take a long time up to 12 h.^[^ ^206^ ^]^ This technique was similar to that reported by Xu et al., where they patterned ssDNA fragments onto Si substrate with CP.^[^ ^126a^ ^]^ SWCNTs were then functionalized with ssDNA that had a complimentary sequence to the prepatterned substrate ssDNA. When the functionalized SWCNT dispersion was placed on the substrate, the complimentary ssDNA on the substrate and SWCNTs hybridized, binding the SWCNTs in patterns to the substrate. This technique could produce sub‐micrometer resolution patterns, but the uniformity and alignment were not ideal. Slightly annealing the substrate aided in improving the alignment somewhat. Penzo et al. demonstrated an innovative end‐bonding technique where end‐functionalized SWCNTs were anchored onto specific patterned locations on a substrate through covalent bonding (Figure 8l,m).^[^ ^126b^ ^]^ SWCNTs with carboxyl or ssDNA segment ends are bonded to nanoimprint‐patterned metallic nanodot anchors with an amine or complimentary ssDNA functionalization, respectively. The nanodot anchors had 2–10 nm diameter with down to 15–20 nm spacing, demonstrating nanoscale resolution patterning. SWCNTs could be anchored at one end (monovalent) to the substrate with up to 93% of the nanodots anchoring a carboxyl‐ended SWCNT. If the spacing between the nanodot anchors and the SWCNT lengths was the same, then both ends of the SWCNTs could be anchored to separate nanodots (bivalent), with 53% nanodots anchoring one end of a double anchored DNA‐ended SWCNT. Although the yield is currently low, the technique demonstrated the ability to pattern CNTs at nanoscale resolution over whole substrates, while simultaneously controlling their orientation and connection across the substrate. More simply though, DNA can be used as a dispersing agent for CNTs (like surfactants) and allows them to be attracted and adhere to hydrophilic patterned regions on a substrate. A big advantage is that DNA functionalization can be used to sort CNTs by chirality. DNA‐dispersed chirality‐sorted CNTs can be patterned at nanoscale resolution with hydrophilic region patterned substrates.^[^ ^126c^ ^]^

### 3.6 DPN

DPN is a direct‐writing patterning technique that has developed to pattern a vast range of materials with potentially nanometer lateral resolution. DPN is similar to a dip pen or quill and inkwell used in pre‐modern times for writing, but down to the micrometer or nanometer scale. A probe with a sub‐micrometer diameter tip, similar to an AFM probe, is dipped into an ink, and traced along a target substrate surface. The ink contains the desired liquid or solid, and while tracing the probe, is transported through the ink meniscus from the probe to the surface in the desired pattern. The clear advantage of DPN is the ability to easily create arbitrary nanometer resolution patterns by preparing an ink of the target material. No masks are required, and it is done in ambient conditions. This is very useful for making many different prototypes or custom made devices. However, it is a serial process requiring each line and dot of target patterns to be traced, and so it has a very slow throughput. There is some recent work to increase throughput of the DPN technique, by creating probes with a large array of multiple pen tips, with some of these arrays having thousands of individual pen tips. This allows DPN to simultaneously pattern thousands of the same pattern periodically spaced on substrates.

**Figure 9:**
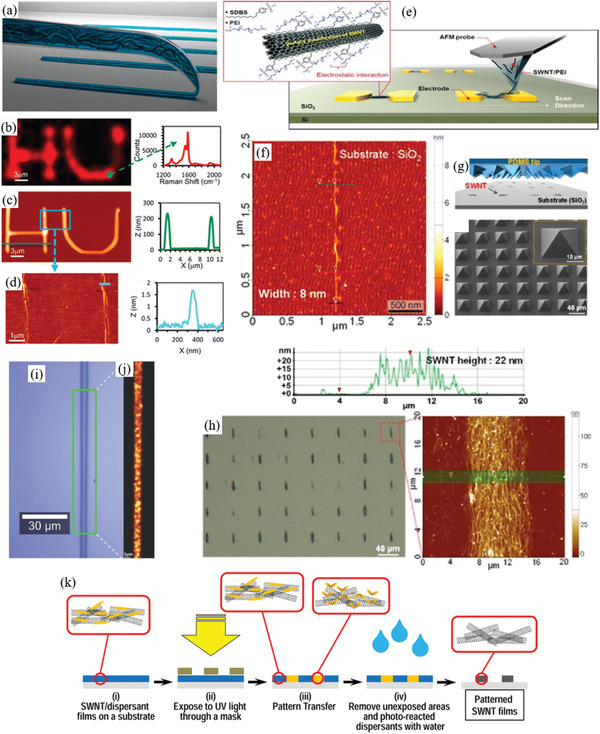
a) Direct‐writing fountain‐pen CNT patterning technique using an aqueous SC/SWCNT ink. b) Raman mapping of the G+ band of the fountain pen patterned SWCNT lines. AFM images and line profiles of the SWCNT line patterns c) before and d) after washing substrate and 4 days drying. Reproduced with permission.^[^ ^207b^ ^]^ Copyright 2016, American Chemical Society. e) Direct nanoscale patterning of SWCNTs on silicon substrate using dip pen nanolithography and SWCNT/SDBS/PEI ink. f) AFM image displaying the minimum width of SWCNT line obtained of 8 nm patterned from DPN. g) Polymer pen arrays with ≈90 000 pens were used to simultaneously pattern h) many line/dot patterns of SWCNTs using high throughput polymer pen lithography (PPL) technique (confocal and AFM images). Reproduced with permission.^[^ ^2d^ ^]^ Copyright 2016, American Chemical Society. i) Optical image and j) Raman map of the CNT G‐band (≈1590 cm^−1^) of the same SWCNT line patterned via DPN. The Raman map clearly reveals the presence and location of SWCNTs on the substrate for determining the quality of the lines patterned. Raman map scale bar 3 µm. Reproduced with permission.^[^ ^2e^ ^]^ Copyright 2018, Wiley‐VCH. k) Method for patterning CNT films via photoresponsive dispersing agents. Reproduced with permission.^[^ ^212a^ ^]^ Copyright 2016, American Chemical Society.

CNTs inks can be deposited onto target substrates using DPN, patterning the CNTs at nanoscale resolution in a simple and effective way. CNTs will also self‐align if the patterned lines are thinner than the average length of the CNTs (Section 2.4.3).^[^ ^2^, ^207^ ^]^ Baba et al. first demonstrated DPN patterning of CNTs, creating SWCNT lines with width down to 100 nm.^[^ ^208^ ^]^ They prepared SWCNT dispersions in ethanol with Alcian bluetetrakis(methyl pyridium) chloride as the ink, dipping the DPN probes into the dispersion and drying with a nitrogen stream to ink the probe. Tracing the probe in contact mode across the substrate deposited the SWCNT in desired line or dot patterns. Relative humidity >70% was required to obtain a sufficient spontaneous water meniscus between the probe and substrate to transport the SWCNTs to the substrate. They also used “fountain‐pen nanolithography” to deposit the SWCNT ink, which uses a microfluidic channel through the pen probe to supply the ink to the probe tip. Strain et al. also reported using fountain‐pen nanolithography to deposit surfactant/SWCNT ink dispersions.^[^ ^207a^ ^]^ However, they required SAM‐functionalized substrates to attract and adhere the SWCNTs from the ink dispersions. Yeshua et al. improved the fountain‐pen technique, using an aqueous dispersion of SC‐dispersed SWCNTs as the ink, and depositing onto substrates without modification (**Figure** **9**a).^[^ ^207b^ ^]^ They also investigated many writing parameters to determine the optimal conditions, allowing them to pattern aligned, conductive SWCNT lines with 10–200 nm width and 0.5–8 µm s^−1^ writing speed (Figure 9b). Washing the substrate after drying the ink for 3 h to 4 days removes much surfactant and leaves behind SWCNTs in the line pattern, although it is not known how many SWCNTs were also removed (Figure 9c,d). Lee et al. used an SWCNT ink composed of surfactant‐dispersed SWCNT mixed with polyethyleneimine (PEI) in aqueous solution to directly pattern the SWCNTs onto SiO_2_/Si substrates using DPN (Figure 9e).^[^ ^2d^ ^]^ The PEI was used to appropriately adjust the viscosity of the ink solution and was removed by methanol vapor wash after patterning. PEI removal was confirmed by the p‐type behavior of subsequently prepared FET devices, in contrast to the strong n‐type doping behavior of PEI.^[^ ^209^ ^]^ They were able to pattern monolayer SWCNT lines with widths down to reportedly 8 nm (Figure 9f). Pen arrays of ≈90 000 PDMS pens were also used to simultaneously pattern many SWCNT dot and line patterns, demonstrating the possibility for high throughput (Figure 9g,h). Corletto et al. extended this technique to pattern SWCNT lines to >1 mm long using a similar SWCNT/surfactant/PEI ink solution.^[^ ^2e^ ^]^ They then patterned these SWCNT lines onto SWCNT/Si heterojunction solar cells to operate as thin conductive pathways through the p‐type top layer, similar to front contact electrodes. The SWCNT lines helped to decrease the average series resistance in the cells by ≈44% and improve the efficiency by 24% relatively. They also demonstrated that the uniformity of SWCNT deposition along the lines could be effectively and easily characterized by mapping the intensity of the CNT G‐band peak in the Raman spectra over the substrate (Figure 9i,j).

### 3.7 Novel Methods

Other more novel nanoscale CNT patterning methods have been explored. One of the simplest conceptually but experimentally difficult was presented by Lefebvre et al., where they used an AFM probe to physically manipulate an SWCNT to a desired location on the substrate.^[^ ^210^ ^]^ An AFM tip is positioned over the SWCNT, and the tip‐to‐sample force is increased to interact with the SWCNT. Quickly scanning the probe can push part of the SWCNT across the sample, but usually at distances ≈10 nm at a time. Although individual SWCNTs can be precisely moved across the substrate, the process is very time‐consuming. Huang et al. used a substrate transfer process to position individual SWCNTs to within micrometer accuracy on target substrate.^[^ ^211^ ^]^ They grew an SWCNT via CVD over gaps up to 500 µm wide, and then inverted the growth substrate over the target substrate at the desired location. They cast polymer over the SWCNT from the back of the growth gap, which held the SWCNT in place while the growth substrate was removed. The polymer could then be removed, leaving a single 100s µm long SWCNT at a target location on the substrate. This is an effective technique to place long SWCNTs at desired locations on a substrate, however it is also a lengthy process if many individual SWCNTs are patterned.

Yoshida's group demonstrated an interesting patterning technique that used light to switch the dispersibility of CNTs through photoresponsive dispersing agents (Figure 9k).^[^ ^212^ ^]^ CNT/dispersant films or solutions were applied over substrate, and then exposed to UV light through a photomask like normal photolithography. The UV light switches the dispersibility of the dispersing agent, aggregating the CNTs onto the substrate at the UV patterned regions. A subsequent solvent wash removes nonswitched CNT/dispersing agent, leaving CNT films at the patterned regions. A similar photoimmobilization method was presented recently by Wang et al.^[^ ^213^ ^]^ They instead used a photosensitive polyfluorene‐based alternative copolymer dispersing agent that selectively reacted and attached to the substrate where it was exposed to UV light. The copolymer could also selectively disperse semiconducting SWCNTs to provide sorting, dispersing, and patterning utilities all in one additive. The advantage of these techniques is their simple implementation. Although only microscale patterns were fabricated, nanoscale features may be possible using cutting edge UV lithography equipment and appropriate dispersing agents.

Fukaya et al. fabricated sub‐10 µm feature lines of CNTs by collecting gas‐phase CNTs synthesized by dry floating catalyst CVD on a resist‐patterned filter template.^[^ ^214^ ^]^ They then could easily dry transfer the CNT film patterns onto polymer substrates for use as transparent conductive films. This technique is dry and requires no solvent or dispersing agent, reducing contamination and maintaining the excellent conductive properties of the CNTs. Although currently only microscale features have been reported, nanoscale features may be implemented with nanoscale templates.

## 4 Subtractive CNT Patterning

CNT films can be patterned after deposition or growth of the film by area‐selective removal of the CNTs in the film. Manufacturing processes that remove material to form the desired morphology are called subtractive processes and removing CNTs from a film to form the desired pattern is subtractive CNT patterning. CNTs are generally removed by destruction or adhesion to another substrate. Subtractive CNT patterning can use the high density and highly aligned CNT films produced over whole substrates through other techniques.^[^ ^215^ ^]^ Thus, these patterning techniques can exploit these higher quality CNT films to create high‐quality and high‐performance CNT patterns, a major advantage over other CNT patterning techniques that must control density and alignment during the patterning process. The techniques can however suffer from defects on the edges of the patterns, as the removal step can damage CNTs at the pattern edges or tear the CNT network irregularly causing a rough edge profile.

### 4.1 Oxidation Etching

CNTs can be eliminated by strong oxidation as the carbon reacts to form CO or CO_2_. Oxidation can be done efficiently by oxygen plasma using a standard reactive ion etching (RIE) process or an inductively coupled plasma reactive ion etching (ICP‐RIE) process. Patterning of CNT films by oxidation etching involves protecting the CNT film in the desired pattern and then using RIE or ICP‐RIE to etch the remaining exposed CNT film, leaving a patterned CNT film. The CNT film is most often protected by photoresist patterned via photolithography or EBL over the CNT film.^[^ ^28^, ^216^ ^]^ This process is relatively simple to perform and uses conventional CMOS manufacturing technology. This has allowed this CNT patterning technique to be seamlessly incorporated into CMOS manufacturing processes and allowed advanced CNT devices to be fabricated.^[^ ^216^, ^217^ ^]^ Even the recently reported advanced CNT computer was fabricating using this CNT patterning technique, demonstrating its viability for commercial application.^[^ ^4^, ^218^ ^]^ However, this process is still limited in its throughput and more costly compared to R2R techniques or other patterning techniques that can be performed in ambient conditions. The etching process can also affect the properties of the CNTs and subsequently affect the device performance. CNT etching produces edge defects on the edge of the patterned areas which can increase resistance and cause charge trapping,^[^ ^101^ ^]^ while photoresist residues can impair the CNT network conductivity and possibly dope the CNT network.^[^ ^216b^ ^]^

**Figure 10:**
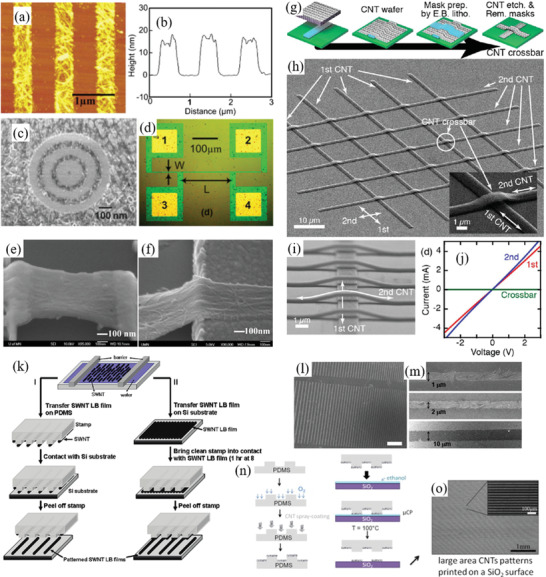
a) AFM image, b) AFM height profile, and c) SEM image of random aligned SWCNT networks patterned via oxidation etching. d) Optical image of four‐point probe structure used to measure resistance through the SWCNT network. Reproduced with permission.^[^ ^28^ ^]^ Copyright 2006, AIP Publishing. SEM images of suspended SWCNT beams patterned via e) FIB and f) oxidation etching through photoresist. Reproduced with permission.^[^ ^216c^ ^]^ Copyright 2009, AIP Publishing. g) Layer‐by‐layer assembly and oxidation etching patterning process to form 3D SWCNT crossbar structures. h,i) SEM images of the fabricated crossbar structures and j) *I*–*V* plots of the two CNT layers and the crossbar point. Reproduced with permission.^[^ ^216d^ ^]^ Copyright 2012, American Chemical Society. k) Schematic depicting SWCNT films aligned via Langmuir–Blodgett method and patterned via CP. The aligned film can be transferred to the stamp and then stamped on substrate (left), or the film can be transferred to substrate and stamp used to remove unwanted regions (right) (lift‐up). l) SEM image of SWCNT lines patterned via lift‐up method (scale bar 200 µm) with m) close‐up of lines. Reproduced with permission.^[^ ^222b^ ^]^ Copyright 2010, American Chemical Society. n) Spray‐coating of SWCNTs on patterned stamp followed by CP onto substrate using ethanol solvent mediator. o) SEM image of SWCNT lines patterned via CP. Reproduced with permission.^[^ ^221d^ ^]^ Copyright 2012, Elsevier.

Zhou et al. demonstrated this technique first in an unassuming mention in the method of a 2004 paper for creating CNT thin film transistors.^[^ ^216a^ ^]^ They synthesized a sub‐monolayer film of randomly aligned SWCNTs and then spun and patterned a PMMA resist layer using photolithography. They then etched the unprotected SWCNTs in the film using oxygen RIE, leaving the desired pattern of SWCNT film underneath the PMMA resist. The resist could be removed if required by solvents. From this method, they were able to produce SWCNT film channels with 3–4 µm widths for use in CNT thin film transistors. The group subsequently used this patterning technique for patterning SWCNT film strips for transistor applications,^[^ ^219^ ^]^ however not with nanometer resolution. Behnam et al. were able to achieve submicron, nanoscale resolution with this technique, creating lines of randomly aligned SWCNT films down to 50 nm wide (**Figure** **10**a–d).^[^ ^28^, ^216^ ^]^ The photoresist in this case was instead patterned by EBL to achieve submicron resolution. They also found that ICP‐RIE had faster etching and better selectivity than conventional RIE, improving the quality of the submicron resolution features.^[^ ^216b^ ^]^ The photoresist was found to affect the doping of the remaining SWCNTs, affecting the resistivity of the patterned films. Hayamizu et al. were able to scale this down to the nanometer scale.^[^ ^188^ ^]^ They used oxidation plasma etching to pattern dense horizontally aligned SWCNT forests into useful 3D architectures for micro/nanoelectromechanical devices. Yamada et al. continued this process, fabricating 3D nanoelectronic devices with dense nanoscale SWCNT crossbar 3D structures via layer‐by‐layer assembly, oxidation plasma etching patterning, and water densification of SWCNT forests (Figure 10g–j).^[^ ^216d^ ^]^ Lu et al. produced dense SWCNT beams down to 320 nm wide suspended between electrodes for testing nanoelectromechanical systems.^[^ ^216c^ ^]^ They used FIB lithography as well as oxidation etching through photoresist to define nanoscale beams (Figure 10e,f). FIB is much slower though. In a similar way, Chae et al. used an excimer laser to pattern CNT films through photoablation.^[^ ^220^ ^]^ A polymer resist was required to be cast on the film that engulf the CNTs in the film and when the resist was ablated, the CNT film in that resist areas was removed with the resist.

The oxidation etching patterning technique is currently one of the most important and common CNT patterning techniques used and likely the most commercially viable. This is due to its similarity to existing micro/nanofabrication techniques and easy incorporation into existing assembly lines. The similarity with existing commercial techniques also means it has increased reliability in production; critically important for commercial manufacturing. However, it is difficult to envision increased performance from this technique, particularly increasing the throughput or making it R2R compatible. Other CNT patterning techniques like the CNT dispersion patterning techniques appear to have a better ability to obtain high throughput and nanoscale resolution simultaneously, although this will need to be investigated further to increase the reliability for production.

### 4.2 CP

CNTs can be patterned simply by using CP.^[^ ^1^, ^126^, ^221^ ^]^ For this patterning technique, CNTs or CNT dispersions are deposited on polymer (usually PDMS) stamps with micro/nanosized patterns. The stamp is then contacted onto a target substrate and only the CNTs/CNT dispersion on the raised features is transferred, leaving the desired stamp pattern on the target substrate. This technique is quick, low cost, and often low temperature. The resolution is limited to the stamp's resolution, but CNT dispersions will spread on the substrate to make wider features than the stamp's features. Line widths can also be increased by CNTs imperfectly attaching to the desired raised area of the stamp, resulting in greater edge roughness of features laterally and vertically. Coverage is also often not always uniform.^[^ ^221a^ ^]^ The transfer process is dependent on the difference in surface energies of the stamp and substrate.^[^ ^221a^ ^]^ Higher surface energies on the substrate are better for efficient transfer, as CNTs adhere better with higher surface energies.^[^ ^221b^ ^]^ Flat surfaces consequently also adhere better, as there will be more contact area between CNTs and substrate. Heating and pressure may also help to adhere CNTs to the stamp.^[^ ^221b^ ^]^ Multiple stamping steps can be applied to the same substrate to create multilayer patterns with different orientations.^[^ ^222^ ^]^ There have been many reports on CP patterning of CNTs, however they most often do not result in sub‐micrometer resolution.^[^ ^1a^ ^]^

This CNT patterning method was first investigated by Meitl et al.^[^ ^221a^ ^]^ They used an innovative controlled flocculation method to cover patterned PDMS stamps with deposited SWCNTs. Controlled flocculation involves applying simultaneously both an SWCNT aqueous surfactant dispersion and methanol solvent on the patterned PDMS stamp during spin coating. The methanol operates as an antisolvent, allowing the SWCNT to controllably deposit and coat the stamp. They then contacted the stamp onto a target substrate, which allowed the SWCNTs deposited on the raised regions to adhere to the substrate in the desired pattern. Curved objects/substrates were also patterned with SWCNTs. Only micrometer resolution patterns were created, and the thickness of the SWCNT film was quite low at ≈5 nm on average. Choi et al. were able to make micrometer‐wide CNT line features with CP patterning, and with alignment of the CNTs in the line features (Figure 10k–m).^[^ ^222b^ ^]^ They first prepared aligned SWCNT films by the Langmuir–Blodgett method. They functionalized SWCNTs with thiophenyl groups so they could then disperse them in chloroform to float on the water bath. The SWCNT monolayer film was then transferred to a patterned PDMS stamp and then transferred to silicon substrates yielding patterns down to 1 µm width. Béduer et al. spray coated CNTs onto a PDMS stamp which formed thick CNT films on the stamp (Figure 10n,o).^[^ ^221d^ ^]^ This method is quick and allows for homogenous coverage but the CNTs are randomly aligned. They also transferred the CNT patterns using a liquid layer on the substrate to promote adhesion. While the authors reported minimum resolutions of 1 µm, nanoscale resolution is plausible if nanoscale feature stamps are used.

## 5 Pre‐Synthesis Patterning

CNTs can be assembled into sub‐micron resolution patterns by employing the patterning step before growth of the carbon nanotubes. CNTs grown via the CVD method of synthesis require nucleation points or growth catalysts, which are commonly transition metal nanoparticles. If the location of these growth catalysts are assembled into desired patterns, then CNTs can subsequently be grown in the desired patterns from the prepatterned growth catalysts. Pre‐synthesis patterning techniques are advantageous in that high CNT density patterns can be fabricated as there are no aggregation issues with CNT dispersions. Also, the patterned CNTs can be aligned perpendicular to the substrate (vertically aligned, standing up) which is useful for many applications. The main disadvantage of these techniques is the high temperature required for CNT synthesis (Section 2.2.4). The high synthesis temperature can negatively affect the substrate or other materials already patterned on the substrate, limiting when and how these CNT patterning techniques are used during the process of device fabrication, and prohibiting its use in back‐end‐of‐line.

### 5.1 Porous Substrate Templates

Different porous substrates with nanoscale pores can be used as templates to guide the growth of CNTs from CNT growth catalysts within the pores. This technique can be limited as the pores on the substrates are generally formed through self‐assembly, although later work used photolithographically defined pores. The technique can achieve nanoscale resolution with regular nanoscale pitch between the grown CNTs. However, the special porous substrate required for this technique is not viable for many applications, and transfer of the resulting CNT patterns to another substrate has not currently been achieved.

One of the initial papers developing this patterning method was published by Li et al. in 1996.^[^ ^71a^ ^]^ They embedded iron nanoparticle catalysts into the pores of a mesoporous silica substrate. The pores on the silica patterned the iron nanoparticles, while also operating as templates during CNT growth. They used CVD to grow individual CNTs with ≈30 nm diameter out of the pores, with an ≈100 nm spacing between grown CNTs, matching the pore spacing. The CNTs were grown highly aligned and perpendicular to the substrate, reaching lengths of up to 100 µm before starting to bend or collect amorphous carbon. This method of using mesoporous materials and embedding the pores with growth catalysts has shown to be an effective sub‐micron CNT patterning technique for certain applications, especially as field emission sources.

Porous anodic aluminum oxide (AAO) templates have also been used to synthesize patterned CNTs. CNTs have been synthesized without catalysts inside the pores of AAO films, using graphitized polymer or pyrolyzed carbon.^[^ ^223^ ^]^ CNT growth catalysts can also be deposited/formed inside the pores to stimulate CNT growth. Li et al. electrochemically deposited cobalt nanoparticles at the bottom of 20–100 nm pores of an AAO film template, to catalyze the synthesis of CNTs in the pores using pyrolysis.^[^ ^73^ ^]^ The length and outer diameter of the synthesized CNTs approximately matches the length and inner diameter of the pores, and so they could control the pore dimensions to control the synthesized CNT dimensions. They also found that the synthesized CNTs had open ends, and so they were able to fill the CNTs with metals such as nickel or cobalt via electroless deposition. Employing a two‐step anodization method for preparing the porous anodized alumina can improve the uniformity and quality of the pores.^[^ ^224^ ^]^ Li et al. used the two‐step anodization method to create 6 µm long and 32 nm diameter hexagonal close‐packed uniform pores.^[^ ^224b^ ^]^ After CVD, they synthesized individual perpendicular‐aligned CNTs with 6 µm length equal to the pores and ≈47 nm diameter, slightly larger than the original pore size due to widening of the pores during synthesis. The density of uniformly shaped and spaced CNTs could reach a very high 10^10^ cm^−2^. Multibranched CNTs can be easily synthesized using AAO templates.^[^ ^223c^ ^]^

The exact pore location is not directly controllable with mesoporous silica or AAO templates, so arbitrary patterns cannot be fabricated. Duesberg et al. used photolithography to define pores in silicon dioxide substrates; controlling the exact location of the pores, and allowing individual CNTs to be grown in desired locations.^[^ ^225^ ^]^ They fabricated 20–60 nm diameter holes in the silicon substrate by fabricating larger holes in amorphous silicon film, and then back filling with silicon to narrow the holes. Fe catalyst was deposited by sputtering and removing excess by ion milling, leaving Fe catalyst only in the holes. CVD of acetylene/hydrogen produced individual CNTs in each hole that was under 60 nm diameter, with the CNT diameter matching the hole. This technique therefore allows the CNT diameter and position to be controlled during CVD growth. Esconjauregui et al. demonstrated a similar technique, except using chemical mechanical polishing to remove excess metal catalyst nanoparticles from the raised features.^[^ ^226^ ^]^ 100–300 nm diameter 600 nm deep pores were etched in a silicon oxide wafer, metal film catalyst was deposited and rapidly annealed to form catalyst nanoparticles, sacrificial polymer was spun on, and chemical mechanical polishing was used to remove catalysts not in the pores. Bundles of CNTs were subsequently CVD grown from the pores filled with catalyst nanoparticles. However, CNTs were not well adhered to the substrate and pores, and longer CNTs were easily lifted off the substrate and out of the pores.

### 5.2 Beam Lithography

Beam lithography is a direct‐write process where up to nanometer lateral resolution patterns can be created on target substrates. The beams can directly synthesize CNT growth catalysts from precursors in desired patterns, or they can modify substrates for selectively depositing CNT growth catalysts on the patterns. Laser beam etching, EBL, and FIB lithography have all been used for CNT growth catalyst patterning. These patterning techniques can produce arbitrary patterns down to 10s of nanometer resolution. However, throughput speed is slow as the beam must directly write the patterns, limiting this technique's applicability to manufacturing. The direct writing of arbitrary patterns can be useful for quickly fabricating different prototypes for testing or for custom‐made devices, and can still achieve very high resolution.

The ability to prepare controllable, arbitrary patterns of growth catalyst for CNT synthesis was pioneered by Terrones et al.^[^ ^227^ ^]^ They were able to pattern lines of cobalt nanoparticle catalysts on a silica substrate by laser etching a Co film and converting into Co catalyst nanoparticles. They created lines of catalyst down to a width of 1 µm and up to 5 mm long. Well‐aligned CNTs were selectively grown from the patterned catalyst by pyrolysis of 2‐amino‐4,6‐dichloro‐*s*‐triazine.

**Figure 11:**
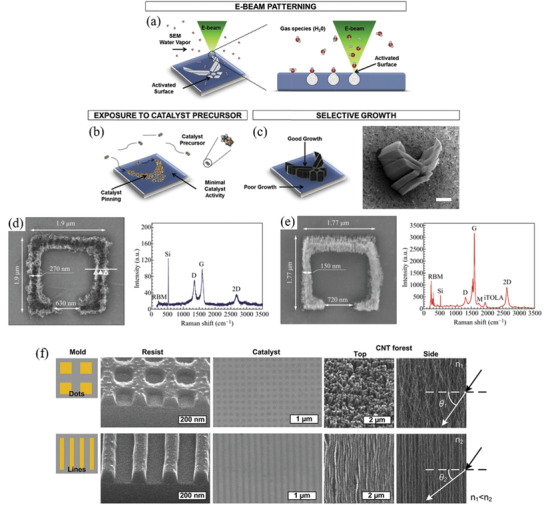
a) EBL patterning of substrate with water vapor to activate substrate. b) Catalyst selectively pins to patterned regions due to increased roughness and basicity. c) CNT forest selectively grows in patterned regions due to patterned catalyst. SEM image demonstrates successful patterned CNT growth (scale bar 10 µm). Reproduced with permission.^[^ ^62^ ^]^ Copyright 2018, Elsevier. SEM images and Raman spectra demonstrating the effect without d) and with e) a secondary FIB etching step to remove redeposited catalyst particles. Higher SWCNT forest density, finer pattern resolution, and higher quality SWCNTs (smaller D‐band in Raman spectrum) are fabricated. Reproduced under the terms of the CC BY 4.0 license.^[^ ^235^ ^]^ Copyright 2017, The Authors, published by Springer Nature. f) SEM images of resist patterned via NIL, catalyst patterned via deposition through the resist, and subsequently grown nanoscale patterns of high aspect ratio CNT forests. Reproduced with permission.^[^ ^244^ ^]^ Copyright 2017, Elsevier.

EBL is a common beam etching method to directly write patterns of catalyst onto a substrate. Metal films can be converted into metal catalyst nanoparticles, or e‐beam precursor films can be converted directly into catalyst in desired patterns with nanometer resolution. Semet et al. used E‐beam lithography to pattern Ni catalyst with nanometer lateral resolution, allowing single ≈5 mm tall ≈60 nm diameter MWCNTs to by vertically grown by plasma‐enhanced CVD of acetylene and ammonia.^[^ ^228^ ^]^ Each MWCNT in the arrays was found to act as field emission cathodes at up to 20 µA, with low current variation. Ishida et al. spin‐coated a Fe‐doped negative EB resist film onto a SiO_2_ substrate, and used EBL to pattern the substrate.^[^ ^229^ ^]^ Negative EB resist is rendered insoluble after e‐beam exposure, leaving only the exposed Fe‐doped negative EB resist film to remain after developing. After heat and oxygen treatment of the developed patterns, the organic components were pyrolyzed/oxidized, leaving patterned individualized Fe catalyst nanoparticles with 1.7 ± 0.6 nm diameter, and an impressive positioning accuracy of ± 5 nm. SWCNTs were subsequently grown from the patterned Fe nanoparticles, however they were not aligned. Patole et al. used EBL to pattern a Fe naphthenate film on Al/Si substrate, and then annealed to reveal patterned Fe catalyst.^[^ ^230^ ^]^ Beams of 100 nA, 25 kV with doses of 8 or 5 mC cm^−2^ could produce line widths of 500 or 100 nm, respectively. The thinner lines only produced a sparse distribution of grown CNTs, unlike the thick forests from the thicker lines. EBL can also be used to modify the growth substrate, allowing catalyst to be pinned to the modified regions. Carpena‐Núñez et al. patterned c‐cut sapphire substrates using EBL in water vapor, which increased surface roughness and basicity in the irradiated areas (**Figure** **11**a).^[^ ^62^ ^]^ Catalyst only deposited and activated in the patterned areas, as the metal reactivity was enhanced and the surface roughness prevented Ostwald ripening of formed metal catalyst nanoparticles (Figure 11b). Water vapor was critical for this method, as it provided neutral oxygen atoms and radiolysis products, which roughened and chemically modified the substrate. CNT forests could then be selectively grown on the patterned areas (Figure 11c).

FIB involves scanning a beam of reactive ions, often Ga^+^ ions, at a substrate to create desired patterns down to nanometer resolution. FIB can be used to either etch away material from a substrate, or to deposit material onto a substrate in controlled patterns. During the deposition process, organometallic gases in the vacuum chamber are decomposed and deposited by the beam into metallic film patterns on the substrate. This technique can deposit CNT growth catalyst in patterns onto a large variety of topographies and substrates, which is useful for applications where nonplanar structures already exist on device substrates. Peng et al. used (FIB) to pattern vapor‐deposited Fe growth catalyst.^[^ ^231^ ^]^ FIB allowed them to pattern the Fe catalyst with 100s nm resolution on a variety of different substrates, including Si, SiO_2_, Al_2_O_3_, and Si_3_N_4_. Hofmann et al. used FIB to deposit Pt film patterns as CNT growth catalyst on untreated Si substrates.^[^ ^232^ ^]^ Annealing the Pt films created Pt nanoparticles with sizes proportional to the film thickness and annealing temperature. They then used plasma‐enhanced CVD (PECVD) to grow single CNT width patterns. Sharma et al. were able to reach true nanoscale resolution by patterning individual 20 nm diameter Fe catalyst nanoparticles with 100 nm spacing on a substrate.^[^ ^233^ ^]^ They used an EB instead to decompose iron nonacarbonyl from the sample chamber at desired locations on perforated SiO_2_ substrate to deposit Fe catalyst particles. Particle size was dependent on beam current, exposure time, and probe size, with exposure times only in the 3–5 s region. The deposited particles were initially agglomerates of smaller iron oxide crystals with different crystallographic directions. Heating the agglomerates for 1–2 min sintered them into a Fe single crystal, and then into Fe_3_C cementite which was the CNT growth catalyst.

A second step of FIB can also be used to reduce the dimensions of the catalyst patterns to nanometer scale, reduce catalyst layer thickness, and remove randomly redeposited catalyst from the first step. Vigolo et al. patterned micrometer dots of Fe or Ni using photolithography, and then used FIB to reduce the dots diameter to 100s nm scale, allowing them to grow single MWCNTs from the reduced size catalyst patterns.^[^ ^234^ ^]^ Pander et al. used a second FIB etching step applied evenly to the whole substrate to remove randomly redeposited catalyst particles from the first patterning FIB etching step.^[^ ^235^ ^]^ This resulted in a decrease in the average roughness of the surface from ≈0.45 to ≈0.15 nm, and consequently higher density CNT forests down to 150 nm lateral resolution were synthesized (Figure 11d,e). Higher‐quality SWCNTs were also produced as indicated by a reduction in the D‐band of the Raman spectrum (Figure 11e). A complication from using FIB etching is that Ga^+^ ions from the beam can deposit on and around the catalyst nanoparticles. This slows the CNT CVD growth rate due to interaction of the catalyst with the deposited Ga. Chen et al. used FIB to etch 10–1000 nm wide trenches in a silicon wafer, and these trenches were used to catch vaporized iron phthalocyanine catalyst from the gas flow during the high‐temperature CVD process.^[^ ^63^ ^]^ CNTs were consequently grown from the catalyst caught in the trenches, creating CNT patterns from the trenches. The authors suggested that the catalyst was preferentially deposited in thinner trenches due to the capillarity effect and wider trenches likely to due to preferred deposition on corners/edges and/or on amorphous surfaces. This technique combines the catalyst deposition step with the CVD step, simplifying the process to pattern CNTs.

### 5.3 Patterned Resist

Polymer resists on substrates can be patterned with a variety of methods for use as templates for patterning growth catalysts. After patterning the resists on a substrate, precursors or catalysts are deposited over the whole substrate, and the patterned resists are lifted off to reveal patterned CNT growth catalyst. Resists can be patterned using conventional photolithography^[^ ^65^, ^236^ ^]^ or E‐beam lithography.^[^ ^237^ ^]^ Then catalyst is deposited onto the substrates by either sputtering catalyst metal films,^[^ ^237a,b^ ^]^ dipping in precursor solution,^[^ ^236^, ^238^ ^]^ or dropping a pre‐formed catalyst nanoparticle solution.^[^ ^65^, ^236^ ^]^ After lift‐off of the photoresist, up to 100 nm resolution patterns of catalyst are revealed on the substrates, and from them CNTs can be grown via CVD. Wong et al. found that PMMA resist produces cleaner lift‐offs than other photoresists.^[^ ^65^ ^]^ They also found that premade Fe nanoparticles can be cleanly patterned via self‐assembly onto the substrate if the particles were capped with oleic acid, allowing them to form more stable colloids. E‐beam lithography of the resists can be used to achieve down to 100 nm resolution patterning, resulting in individualized CNTs.^[^ ^237^, ^238^, ^239^ ^]^

Javey and Dai demonstrated controlled clustering of metal atoms into monodisperse metal nanoparticle catalysts down to ≈1 nm diameter, which were used to grow individualized monodisperse SWCNTs in desired locations with ≈200 nm pitch.^[^ ^64^ ^]^ EBL was used to pattern 40 nm diameter wells in photoresist, < 2 nm precursor metal thin films were evaporated and the photoresist lifted off to reveal precursor metal thin film circles. The metal film was then annealed at 700–900 °C, forming individual discrete metal nanoparticles at the well locations by diffusion and beading up of the metal atoms. The nanoparticle diameter was controlled by the thickness of the precursor metal films, with diameter distributions of ≈5 and ≈17% for ≈7 and ≈2.2 nm particles, respectively. Over a critical well diameter, multiple nanoparticles were formed during the annealing process. Häffner et al. were able to use E‐beam lithography with insulating resists by first sputter depositing the metal catalyst films and then the resist.^[^ ^237b^ ^]^ This provided the required conducting substrate for the resist during the E‐beam lithography step. They used a negative hydrogen silsesquioxane resist, which allowed the exposed resist to remain after developing, providing a barrier during ion etching of the metal catalyst films. The patterned metal catalyst layer was used to CVD grow SWCNTs with 0.8–2 nm diameters in patterns with a very high 10s nm lateral resolution. Kim et al. were able to produce nanometer‐resolution Ni nanoparticle catalyst patterns by controlling the wet‐etch of the precursor Ni film during patterning.^[^ ^240^ ^]^ They found longer etching times of the Ni film could laterally etch the film underneath the photoresist patterns, resulting in thinner line widths than possible with only photolithography. Vertically aligned CNTs under 50 nm diameter were subsequently grown with plasma‐enhanced CVD of acetylene and ammonia.

Vieira et al. pushed this technique to 10s of nm lateral resolution by employing

NIL to pattern the polymer resist.^[^ ^241^ ^]^ NIL presses a hard master with potentially sub‐10 nm features into a polymer resist that is coating the target substrate at high temperature and pressure, so that the polymer resist is above its glassy transition temperature. This molds the polymer resist into the negative of the hard master, and after RIE of the residue, a sub‐nm resolution pattern forms in the polymer resist. The advantage of NIL is the sub‐nm resolution patterning over a large substrate area simultaneously, allowing higher throughput than other sub‐nm resolution techniques like E‐beam lithography. This technique has been scaled up to full 2 in. NIL stamps for mass production.^[^ ^242^ ^]^ Vieira et al. patterned a PMMA resist with NIL to make 270 nm diameter holes, to sputter deposit 7 nm thick Ni catalyst layer circles.^[^ ^241^ ^]^ After PECVD, individual MWCNTs were synthesized with ≈60 nm tip diameters and ≈3.5 µm lengths. The tip diameters were smaller than the pattern catalyst diameters due to a slight tapering of the MWCNTs, by etching of the perimeter Ni by high‐energy ions, and by coalescence of the Ni atoms toward the center of the pattern. Chang et al. patterned 200 nm diameter circles of Fe catalyst on TiN electrode substrate with NIL, consequently producing individualized 50–100 nm diameter MWCNTs.^[^ ^243^ ^]^ Park et al. used NIL to fabricate 80–350 nm grating patterns of up to 500 µm high CNT forests on a Si substrate (Figure 11f).^[^ ^244^ ^]^ They patterned a polymer resist with NIL, deposited alumina and Fe catalyst, then removed resist to reveal Fe/alumina patterns with nanoscale resolution over millimeter‐scale areas. The patterned CNT forests with impressively high‐aspect ratio (>1000:1) had controllable refractive index based on the pattern density.

Even scanning probe nanolithography can be used to pattern resists. This involves using conventional AFM tips in contact mode to scratch the polymer resist away in desired patterns. Parisse et al. created ≈350 nm wide trenches in PMMA resist and subsequently deposited Ni catalyst, which was used to grow ≈40 nm diameter CNTs from the resulting Ni catalyst patterned areas.^[^ ^245^ ^]^ Issues with the technique are poor throughput speed from the serial process, and currently poor line edge roughness due to ripping of the resist. A simpler way to use a resist is simply apply an ink‐based temperature‐resistant polymer over a catalyst‐coated substrate and grow CNTs via CVD from the uncovered regions. Li et al. used common marker ink containing the dispersant styrene‐maleic anhydride (SMA) copolymer and patterned it on Al foil using a regular household printer, or on Si substrates by laser etching an SMA film.^[^ ^246^ ^]^ During the CVD process, ferrocene was deposited onto the uncovered regions of the substrates, creating Fe catalyst and growing CNTs in desired patterns. They showed that the ferrocene precursor only adheres to the substrate and not the polymer, and that a high enough concentration of polymer is required to prevent adhesion. However, only 10 µm lateral resolution patterns could be produced so far.

Patterning CNT growth catalysts through resists is a relatively easy and simple patterning technique for pre‐synthesis patterning. Standard photolithography techniques are required, so the process can be incorporated into current device manufacturing facilities easily. However, most resist patterning methods are batch methods and are often slow, limiting throughput. NIL is the exception as it can pattern quickly over whole wafers at nanoscale resolution. NIL is therefore the most promising technique for efficient and quality CNT patterning using patterned resists.

### 5.4 Shadow Masks

Shadow masks present a scalable way to pattern CNT growth catalysts, as they can pattern whole substrates simultaneously. The technique is also relatively simple and quick compared to other patterning methods. However, the shadow mask itself can be difficult/costly to fabricate, which is inconvenient if different changing patterns are often desired. There are also difficulties with preparing arbitrary patterns of nanoscale dimensions, mainly because the shadow mask must be strong enough to survive handling. Periodic, nonarbitrary patterned shadow masks have also been prepared through self‐assembly methods, including using nanosphere monolayer^[^ ^247^ ^]^ and block copolymer^[^ ^248^ ^]^ self‐assembled templates.

**Figure 12:**
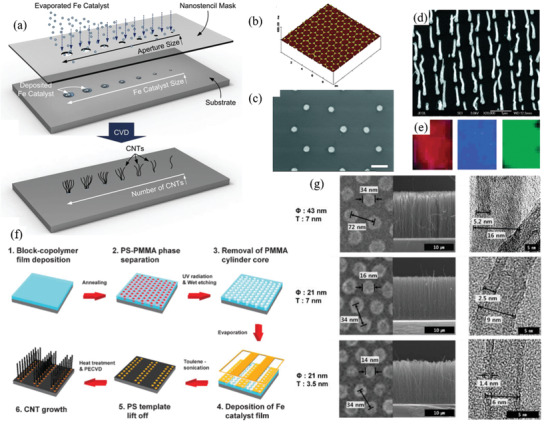
a) Using a shadow mask for nanoscale patterning of CNT growth catalyst. Reproduced under a CC‐BY 4.0 license.^[^ ^249^ ^]^ Copyright 2013, The Authors, published by Springer Nature. b) AFM image of catalyst metal layer on substrate patterned through a self‐assembled nanosphere mask. Image 10 × 10 µm. c) AFM image of catalyst nanoparticles formed into hexagonal formation. Scale bar 200 nm. d) SEM image of subsequently grown CNTs in hexagonal formation. Scale bar 1 µm. e) Diffraction colors produced from hexagonal CNT array. Reproduced with permission.^[^ ^250^ ^]^ Copyright 2003, American Chemical Society. f) Self‐assembled nanoscale shadow mask produced via phase separation of block copolymers followed by patterning of CNT growth catalyst. g) SEM and TEM images showing varying catalyst diameters and spacings, CNT dimensions due to varying nanopore size (*Φ*) and catalyst film thickness (*T*). Reproduced with permission.^[^ ^248^ ^]^ Copyright 2008, Wiley‐VCH.

Fan et al. implemented patterning of CNT growth catalyst by employing a simple shadow mask during the catalyst deposition step.^[^ ^71b^ ^]^ A 5 nm Fe film was E‐beam deposited through a shadow mask, and then oxidized to fabricate patterns of iron oxide nanoparticle catalyst on porous silica substrates. They were able to create highly aligned vertical arrays of ≈16 nm MWCNTs in desired arbitrary patterns. The dense packing of the catalyst nanoparticles aided in the array alignment, as the growing MWCNT outer walls interacted and strengthened each other. However, they only achieved micrometer lateral resolution. Choi et al. were able to push shadow mask patterning down to nanometer resolution by using a low‐stress silicon nitride membrane shadow mask.^[^ ^249^ ^]^ They deposited Fe catalyst via electron beam evaporation through nanoapertures on the shadow mask, which had diameters down to 40 nm and spacing down to 260 nm (**Figure** **12**a). Significant blurring occurred, with some deposited catalyst island diameters being more than double the respective nanoaperture diameter. They suggested decreased gap distance between the shadow mask and substrate causes decreased blurring. CNTs were then CVD grown at the location of the patterned catalyst, with the number of CNTs grown proportional to the Fe catalyst island diameter.

An innovative and simple way of assembling a mask is by using self‐assembled nanospheres layers on the substrate. Monodisperse polystyrene nanospheres placed onto a hydrophilic silicon wafer with surfactant will self‐assemble into a monolayer with a tight hexagon packing.^[^ ^247^ ^]^ This nanosphere monolayer can then be used as a shadow mask while evaporating a catalyst metal, resulting in catalyst metal layer only depositing in the spaces between nanospheres (Figure 12b).^[^ ^250^ ^]^ Upon PECVD of the substrate as demonstrated in their earlier work,^[^ ^237a^ ^]^ catalyst nanoparticles form in a hexagonal formation (Figure 12c) and vertical CNTs can grow from them (Figure 12d). Density and spacing of CNT arrays grown from this technique can be varied by simply controlling the nanosphere diameter. Bilayers of nanospheres can also be used, which can reduce the fabricated dot size by 33% and increase the spacing by 1.73 times.^[^ ^251^ ^]^ Using bilayers instead of reducing nanosphere diameter when increasing spacing will help to avoid multiple CNTs growing from the same dot pattern. These fabricated periodic arrays of CNTs reflect and diffract light in a way to appear strongly colored (Figure 12e).^[^ ^250^ ^]^ The CNT arrays can also behave as a 2D photonic band gap crystal with a band gap in and around the visible frequency range.

Ryu et al. were able to prepare monolayers of packed nanospheres with diameters down to 50 nm using spin coating, allowing for deposition of periodic catalyst patterns with nanometer lateral resolution.^[^ ^252^ ^]^ Upon heating of the patterned catalyst substrate during the CVD process, the triangular catalyst metal film patterns coalesced into spherical nanoparticles with volume dependent on the deposited metal film thickness, diameter of the nanospheres used, and angle of the metal deposition process. They produced well‐aligned periodic SWCNT arrays with CNT diameters of 1.8 ± 1.0 nm. Papadopoulos and Omrane applied the nanosphere monolayer patterning technique to deposit periodic catalyst patterns onto a 〈100〉 silicon wafer, growing from them straight and orthogonal ≈1.4 nm diameter SWCNTs.^[^ ^253^ ^]^ Man et al. simplified the deposition of metal catalyst by dipping the substrate with nanosphere monolayer into an aqueous solution of Fe(NO_3_)_3_ precursor, and heat treating the substrate to burn off the nanospheres and convert the patterned precursor to Fe_2_O_3_ catalyst.^[^ ^254^ ^]^

Block copolymers may be applied to substrates to self‐assemble periodic nanotemplates that can be used as shadow masks to pattern CNT growth catalyst. 5–50 nm periodic features can be assembled via this process, in a parallel process that can be scaled up. However, only specific periodic patterns based on the block copolymer and conditions used can be fabricated, not arbitrary patterns. Lee et al. used polystyrene‐*block*‐poly(methyl methacrylate) (PS‐*b*‐PMMA) to create uniform circular nanopore arrays with 34 and 72 nm spacing in a hexagonal formation (Figure 12f).^[^ ^248^ ^]^ Fe catalyst was then patterned through an additional micrometer‐scale shadow mask to determine large‐scale distribution. This resulted in Fe catalyst deposited in nanometer diameter circles in desired areas of the substrate, which upon heating formed into catalyst nanoparticles of proportional diameter. Uniform diameter CNTs were subsequently grown from the deposited catalyst. They stated that the nanopore diameter is proportional to the molecular weight of block copolymer to the 3/2 power. Also, the synthesized catalyst nanoparticle diameter and consequently CNT diameter are proportional to the nanopore diameter. They suggested and demonstrated that this means the synthesized CNT diameter can be controlled simply by varying the molecular weight of the block copolymer used, along with the deposited catalyst film thickness (Figure 12g).

### 5.5 Self‐Assembly

Diblock copolymers can be phase separated into nanoscale periodic patterns under controlled conditions. These self‐assembled diblock copolymer patterns can be used to pattern CNT growth catalyst by inserting the metal catalyst into one of the polymer blocks.^[^ ^255^ ^]^ Through controlling the polymer composition, molecule weight, and self‐assembly conditions, catalyst can be patterned with controllable size, period, and shape. After self‐assembly of the periodic pattern, the polymer is etched in a variety of ways to leave the metal catalyst in the desired periodic pattern. Lu et al. used polystyrene‐*b*‐polyferrocenylsilane diblock polymer, which contains Fe in one of the blocks, to self‐assemble into hexagonally packed cylindrical domains of the Fe block with 15.8 nm diameter and 30.1 nm spacing.^[^ ^255a^ ^]^ After UV‐ozone treatment and thermolysis, Fe catalyst nanoparticles were formed in a silica matrix, with the silica matrix helping to prevent coalescence of the Fe atoms and keeping the Fe nanoparticle diameters small. However, the CNT growth direction was not controlled and random, leading to random networks of CNTs connecting the catalyst islands. Similar work has been done using other catalyst‐loaded block copolymers, including polystyrene‐*b*‐polyferrocenylethylmethylsilane^[^ ^256^ ^]^ and FeCl_3_‐loaded poly(styrene‐block‐acrylic acid)^[^ ^257^ ^]^ diblock copolymers.

### 5.6 CP of Catalyst

CP involves using a stamp with CNT growth catalysts in ink dispersions adhered to it, and pressing the stamp onto the desired substrate. This technique is simple, low cost, and often low temperature. However, resolution is limited to the resolution of the fabricated stamp, and ink will also spread on the substrate to make wider features than the stamp's features.

Kind et al. first explored this technique, depositing and drying Fe(NO_3_)_3_⋅9H_2_O ink solution onto PDMS stamps, and transferring the Fe CNT growth catalyst patterns onto silicon substrates.^[^ ^258^ ^]^ They then used CVD to grow 8–20 nm diameter MWCNTs from the Fe catalyst in the desired patterns. They found that an advantage of using CP was the density of grown CNTs in the pattern could be controlled proportionally by the concentration of catalyst in the ink solution. However, too high concentrations (>150 × 10^−3^ m) led to no CNTs and only amorphous carbon, due to the catalyst particles aggregating too much for catalyzing CNT growth. Huang et al. first achieved submicron lateral resolution with this technique.^[^ ^259^ ^]^ Instead of directly patterning catalyst though, they deposited hydrophobic OTS SAM in patterns on the substrate. Diazonaphthoquinone (DNQ)‐modified cresol novolak photoresist was then deposited in the negative areas by submerging the patterned substrate in solution, as the photoresist only deposited on the hydrophilic OTS‐free regions. The polymer was converted to carbon black through pyrolysis, which Fe catalyst particles adhered to in the desired patterns from precursor iron(II) phthalocyanine (FePc) gas. Robertson's group demonstrated submicron patterning of Co nanoparticle catalyst with CP.^[^ ^232^, ^260^ ^]^ They fabricated silicon wafer master molds with V and pyramid shapes by anisotropic etching the wafers with KOH or FIB etching, and then created stamps by curing PDMS on the master molds. Stamp feature tips had sub‐50 nm widths, allowing 100 nm lateral resolution patterning of the Co catalyst. Increased loading of the Co catalyst resulted in broadening of the patterned line or dot diameters, as more Co catalyst was transferred to the substrate. Using PECVD, they were able to synthesize vertically aligned CNTs from the patterned Co catalyst, with smaller dot patterns producing individualized CNTs. They found that Co nanoparticle catalysts can produce quite uniform diameter CNTs.

### 5.7 Catalyst Deactivation

Rather than patterning CNT growth catalysts, deactivating metals can be patterned on a substrate instead. Certain reactive metals like Cu and Ag will deactivate CNT growth catalysts when contacting the catalysts. The catalysts can consequently be deactivated where the deactivating metals are patterned, and CNTs can be grown in the negative pattern where the catalysts are still active. This technique is useful as a compliment to other catalyst patterning methods when the catalyst material is more difficult to pattern. This complimentary technique may also be more suitable for particular stages of the device fabrication process. However, the technique is still not well understood and still requires high‐temperature CNT synthesis which restricts its application.

**Figure 13:**
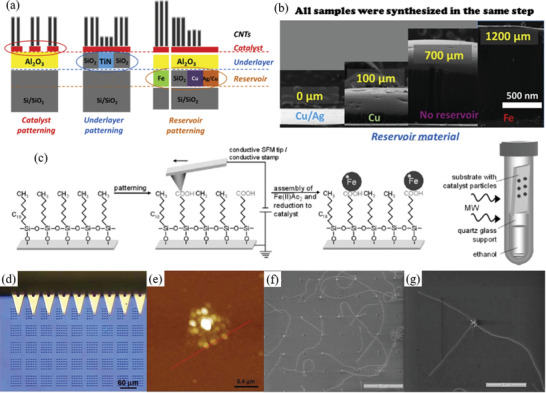
a) Patterning reservoirs of catalyst (de)activating metals can create regions of varying CNT growth speed within the same CVD growth process. b) SEM image of CNTs forests grown over various reservoir materials in the same CVD growth process. Cu/Ag regions will completely inhibit growth, while Fe regions will increase growth rate. The height of the grown CNT forest is displayed. Reproduced with permission.^[^ ^261^ ^]^ Copyright 2018, Elsevier. c) Electro‐chemical patterning of an SAM using a probe tip, followed by selective assembly of Fe metal catalyst onto patterned regions. The substrate was then irradiated with microwaves resulting in localized heating of the Fe catalyst and fast CNT growth. Reproduced with permission.^[^ ^75b^ ^]^ Copyright 2009, Wiley‐VCH. d) Optical (scale bar 60 µm) and e) AFM (scale bar 0.4 µm) images of Fe metal catalyst patterned by DPN with Fe‐based ink. f,g) SEM images of CNTs grown from the DPN‐patterned catalyst (scale bars 10 µm, 2 µm). Reproduced with permission.^[^ ^268^ ^]^ Copyright 2009, Wiley‐VCH.

There are multiple theories as to why these deactivating metals inhibit CNT growth. These include: they encourage rapid Ostwald ripening of catalyst nanoparticles to excessive sizes,^[^ ^261^ ^]^ they prevent formation of required C—C bonds during CNT growth,^[^ ^262^ ^]^ they have very low carbon solubilities preventing diffusion through Cu catalyst nanoparticles.^[^ ^261^, ^263^ ^]^ More investigation into defining the exact mechanism of deactivation and quantitative analysis of the process is required before this patterning technique is commercially viable. However, there has been some preliminary research into the technique recently. Yemini et al. simply annealed an Fe catalyst‐coated substrate while a Cu stencil was on it, diffusing the Cu into the catalyst and deactivating it in the stencil pattern.^[^ ^264^ ^]^ The deactivated catalyst pattern is most defined when the Cu stencil is flat directly on the catalyst layer, otherwise blurring can occur. An ingenious alternative was demonstrated by Shawat Avraham et al. They patterned catalyst deactivating metals Cu or Cu/Ag underneath an alumina layer and placed a Fe catalyst layer on top of the alumina layer (**Figure** **13**a).^[^ ^261^ ^]^ They used standard EBL and EB evaporation to produce the patterns of deactivating metals. Cu/Ag atoms can diffuse through the alumina layer when annealing, deactivating the catalyst directly above the Cu/Ag patterned regions by alloying with the metal catalyst. The addition of Ag with Cu creates a eutectic alloy that has a lower melting point than pure Cu, allowing faster diffusion and better suppression of CNT growth. The catalyst metal like Fe can also be patterned underneath the alumina layer to instead promote increased growth of the CNTs.^[^ ^265^ ^]^ Varying the composition of the deactivating metal layer can be used to control varying levels of CNT growth in desired patterns simultaneously, broadening the ability of the technique (Figure 13b).

### 5.8 Probe Nanolithography

A variety of probe lithography techniques have been investigated for patterning of CNT growth catalysts, each with different benefits and advantages. AFM involves scanning a nanometer scale probe along a target substrate to obtain nanometer resolution topographical, electrical, and mechanical information about the substrate. This same probe can also be used to modify the substrate surface with nanometer resolution. However, it also is a serial and slow throughput process, and so is not currently suitable for mass production. Applying a potential between the probe and the surface while scanning the probe in the desired pattern can result in nanometer resolution patterns of surface oxidation on an otherwise unoxidized surface; this is AFM nano‐oxidation. This technique is effective in creating hydrophilic SiO_2_ nanoscale patterned areas on hydrophobic Si wafers but can also be used to oxidize other surfaces. AFM nano‐oxidation can be used to pattern CNT growth catalyst by the influence of the substrate oxide patterns. Chiu et al. patterned nanoscale oxide features onto a bare Si substrate, which encourages growth of Fe catalyst nanoparticles only on the oxide‐patterned areas.^[^ ^266^ ^]^ After creating the oxide patterns in the substrate using AFM nano‐oxidation, an Fe thin film was deposited over the whole wafer. They then annealed the substrate at 750 °C, which formed iron silicide on the bare Si areas, but Fe nanoparticles on the SiO_2_ areas. CNTs could then be grown from the Fe catalyst nanoparticles patterns in nanometer resolution. The probe with a bias of 8 V was scanned at 0.25–2 µm s^−1^ for lines and held for ≈2 s for dots, creating oxide line widths down to 100 nm and dot diameters down to 50 nm. SAMs of various molecules can be oxidized on substrate surfaces to create patterned regions with different chemistries for further modification. Druzhinina et al. electro‐chemically oxidized patterns of OTS SAM using a probe tip, converting the end methyl groups to carboxylic acid groups (Figure 13c).^[^ ^75b^ ^]^ The acid groups preferentially attracted precursor iron(II) acetate ions which were then reduced to Fe catalyst nanoparticles located on the oxidized patterns. They then synthesized CNTs in minutes from these Fe catalyst nanoparticles patterns using microwave synthesis, keeping the substrate temperature low (<150 °C) due to selective heating of the Fe nanoparticles.

DPN can also be used to easily pattern CNT growth catalyst, with inks made from suspensions of metal nanoparticle catalysts (Section 3.6). Li et al. used a suspension of trioctylphosphine oxide and oleic acid capped Co nanoparticles as the ink for creating dots and lines of Co nanoparticles with features down to 68 nm.^[^ ^267^ ^]^ From the patterns, ≈1.2 nm diameter SWCNTs were grown via CVD. They found that smaller features had a lower SWCNT uniformity and yield a longer contact time resulted in wider features and multiple nanoparticle layers, due to more time for the ink to transport from the pen tip to the substrate. Kuljanishvili et al. showed Fe catalyst can be deposited with DPN, using an ink containing iron salts of ferric nitrate nonahydrate and ferric chloride hexahydrate (Figure 13d–g).^[^ ^268^ ^]^ Concentration of the iron salts in the ink determined eventual Fe catalyst concentration in the patterns. They also demonstrated the scaling up of this method, using a pen array with multiple pens to simultaneously pattern multiple areas (Figure 13d). The minimum features of catalyst that can be printed is due to factors including ambient temperature and humidity, ink composition (including adulterants), ink viscosity, and probe/surface functionalization. Other metal solutions like ferritin, Al_2_O_3_–FeMo, or NiCl_2_ can also be used as DPN inks to create nanoscale patterns of CNT growth catalysts.^[^ ^269^ ^]^

## 6 Pattering During Synthesis

During CVD growth of the CNTs from a patterned catalyst, controlling the growth direction may be achieved with certain methods. This is critical for certain applications that require the grown CNTs to connect particular areas in a device or align in a certain direction.

Terrones et al.^[^ ^227a^ ^]^ grew CNTs from pattern lines of Co catalyst nanoparticle on a silica substrate. Serendipitously, they found the CNTs grew well aligned. They hypothesized that the cause of alignment was overcrowding during growth, sterically controlling the growth direction. Similarly, Fan et al. created highly aligned vertical arrays of ≈16 nm MWCNTs in desired arbitrary patterns via shadow mask deposition of Fe catalyst. They suggested that dense packing of the catalyst nanoparticles aided in the array alignment, as the growing MWCNT outer walls interacted and strengthened each other. Ren et al. suggested that alignment during PECVD may occur by the catalyst nanoparticles capping the growing CNTs, encouraging vertical growth only at the catalyst end of the CNTs.^[^ ^270^ ^]^ Using PECVD of acetylene and ammonia, they grew highly aligned CNTs from nickel‐coated glass at 666 °C, and no patterning or pores were required to produce the alignment. However, Bower et al. demonstrated that the plasma in PECVD causes an electric field perpendicular to the substrate surface, which aligns the CNTs normal to the substrate.^[^ ^271^ ^]^ When the plasma was off in the same method, curly unaligned CNTs were synthesized. This mechanism of vertical alignment was confirmed by other groups.^[^ ^58^, ^228^, ^237^ ^]^ Matsuda et al. demonstrated that a sufficiently negative bias must be applied to the substrate to obtain vertical alignment during PECVD.^[^ ^237c^ ^]^ Their results suggest that the alignment effect is likely due to electrostatic effects on the CNTs. Any positive bias resulted in no alignment too, and they reasoned this was because a negative bias pushed electrons to the tips of the growing CNTs, allowing the tips to repel nearby CNTs and repel from the substrate. Other experiments using PECVD have also shown this vertical alignment effect.^[^ ^240^, ^248^, ^272^ ^]^

### 6.1 Growing Suspended CNTs

Catalyst patterning can enable creative approaches to fabricating suspended CNTs for electronics and sensing applications. Target areas on devices can be patterned with growth catalyst, enabling researchers to grow CNTs that bridge electrodes and other areas in the devices. Suspended CNTs avoid van der Waals interactions with the substrate, vastly improving both their electric properties and sensitivity to the environment for sensing.

**Figure 14:**
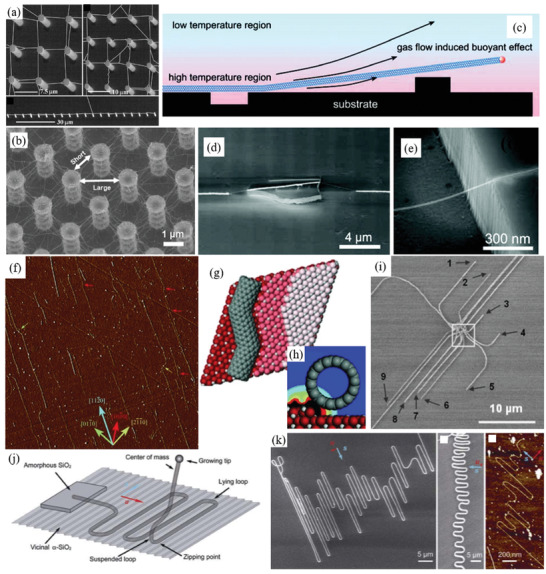
a) SEM images of suspended SWCNTs grown from silicon pillars bridging to other pillars. Reproduced with permission.^[^ ^274^ ^]^ Copyright 2000, Wiley‐VCH. b) SEM image of 3D network of SWCNTs suspended between silicon pillars before casting of PDMS. Reproduced with permission.^[^ ^281^ ^]^ Copyright 2015, Wiley‐VCH. c) Schematic of the buoyant effect on the growing CNT induced by gas density/temperature gradients in low rate gas flow. Note the growing head of the CNT is raised so it can go over obstacles as it grows. SEM images of d) an SWCNT grown over a raised obstacle and e) an SWCNT grown suspended over a trench. Reproduced with permission.^[^ ^286^ ^]^ Copyright 2007, American Chemical Society. f) AFM image and g) model of SWCNTs grown along atomic steps of miscut crystalline sapphire substrates, even growing with kinks to match the atomic steps. The colored arrows on the AFM image indicate the direction of the atomic step edges on the substrate which dictate the growth direction. h) Model of an SWCNT along an atomic step with color gradient representing the interaction energy, demonstrating the increased interaction near the atomic step edge. Reproduced with permission.^[^ ^274^, ^288^ ^]^ Copyright 2004, Wiley‐VCH. i) SEM image of “sickle” SWCNTs grown from DPN‐patterned Co catalyst island on quartz. “Sickle” SWCNTs grow in random direction initially and then align to crystallographic direction after a certain length. Reproduced with permission.^[^ ^267^, ^274^ ^]^ Copyright 2008, Wiley‐VCH. j) Proposed growth mechanism of “serpentine” CNT patterns by directing gas flow (red arrow) perpendicular to the crystallographic step edges (“falling spaghetti” mechanism). k) SEM and AFM images of “serpentine” CNT patterns grown on crystallographic substrates. Red and blue arrows indicate gas flow direction and step edge, respectively. Reproduced with permission from open access.^[^ ^288d^ ^]^ Copyright 2009, Tsinghua University Press and Springer Nature.

One of the earliest papers on growing suspended CNTs with patterned catalyst was by Cassell et al. in 1999.^[^ ^273^ ^]^ They applied a liquid catalyst precursor solution to the top of an array of Si pillars, by applying the solution to a flat PDMS stamp, and then contacting the stamps to the top of the pillars on the substrate, leaving the catalyst precursor solution only on the pillar tops. After calcination, iron nanoparticles on alumina/silica were formed on the pillar tops, which acted as catalysts for SWCNT CVD growth. CVD of methane at 900 °C produced suspended SWCNTs that could bridge the catalyst pillar tops. The authors suggested that the growth direction was random from the pillar top catalyst, with some SWCNTs randomly grown toward other pillars and suspended, while other SWCNTs were grown in other directions and fell along the sidewalls of the pillar due to their own weight. Franklin and Dai used an enhanced growth method to grow up to 150 µm long SWCNTs from Si pillar tops (**Figure** **14**a).^[^ ^274^ ^]^ The increased length allowed the SWCNTs to exhibit strong alignment with the CVD gas flow, allowing single SWCNTs to grow suspending many pillar tops in the same direction. The increased length of the SWCNTs increases the effect of the gas flow “wind” and the SWCNTs and aligning them. Homma et al. investigated further the growth mechanism and the cause of the growth direction and suspension of CNTs.^[^ ^275^ ^]^ They fabricated an array of up to 100 nm diameter Si pillars topped with Fe or Co catalyst, and through CVD of methane‐synthesized SWCNTs from the pillar tops, with ≈44% bridging to another pillar top, and of those ≈85% bridging to the nearest neighbor pillar top. They found that when pillar spacing is comparable to pillar height, most CNTs were bridging to the nearest neighbor. Too large spacing resulted in CNTs contacting the substrate surface. They calculated that lift on the SWCNTs due to methane flow is not strong enough to overcome electric field aligning forces, and so is not responsible for the suspended growth. They also considered vibration and bending of the SWCNTs during CVD growth, as the vibrating cantilever end of the SWCNTs may contact another pillar top and form a bridge. Assuming the SWCNTs are anchored parallel with the pillar top surface and the base is fixed to that plane, they calculated that the thermal and mechanical vibrations of the SWCNT end would not have a large enough amplitude to contact a neighboring pillar. However, assuming that the growing SWCNTs are only anchored to a catalyst nanoparticle and the SWCNTs can swing or revolve around the nanoparticle, the mechanical/thermal vibration amplitude would be large enough to contact neighboring pillars and adhere to them, forming a bridge. This large swing amplitude helps to explain the large percentage of SWCNTs that were bridging nearest neighbor pillars.^[^ ^275^, ^276^ ^]^ Jung et al. improved the technique's yield and CNT density, interconnecting all the neighboring Si posts with bridging CNTs.^[^ ^277^ ^]^ This improvement was mainly through using a SiO_2_ substrate instead of Si, which allowed more stable catalyst nanoparticles to form.

Han et al. in 2001 demonstrated the fabrication of laterally grown MWCNT between nickel catalyst lines patterned with photolithography.^[^ ^278^ ^]^ The 50–100 nm nickel catalyst layer was capped by a 50 nm SiO_2_ layer deposited by microwave plasma‐enhanced CVD. This left only the sidewall of the nickel lines exposed during the 700–1000 °C CVD growth of the MWCNTs, resulting in horizontal growth direction and suppression of vertical growth. The synthesized MWCNTs had diameter proportional to the nickel layer thickness, and single MWCNT bridge lengths were between 0.8 and 2 µm. They also found that higher CVD temperatures resulted in fewer impurities, more wall layers, and lowered resistance in the MWCNTs, likely due to the increased carbon atom diffusivity from higher temperature. However, there was no discussion about the ability to control the location of the synthesized MWCNT bridge along the nickel lines, and it may be that the location is uncontrolled and random. Kong et al. and Franklin et al. instead fabricated laterally grown SWCNTs between alumina‐supported iron catalyst islands.^[^ ^279^ ^]^ The catalyst islands are patterned by applying a catalyst suspension on patterned photoresist, and lifting off the resist to leave patterned catalyst islands on the substrate. They could then grow individualized 1–3 nm diameter SWCNTs with methane CVD at 900–1000 °C. This method allowed Franklin et al. to pattern the catalyst directly onto molybdenum electrodes, effectively bridging the electrodes with 3–10 µm long suspended SWCNTs.^[^ ^279b^ ^]^ Only molybdenum was compatible as electrode material due to the high temperature of CVD growth for synthesizing the suspended SWCNTs, with gold, titanium, tantalum, and tungsten electrodes failing. Ti and Ta become highly resistive, Au melts and balls up, and W inhibits CNT growth. With this technique, Franklin et al. were able to fabricate CNTFETs with p‐type characteristics and a high carrier mobility of ≈10 000 cm^2^ V^−1^ s^−1^, as well as electromechanical sensors. The reason for the SWCNTs to grow preferentially in the direction of the opposing electrode's catalyst island was not discussed, however up to 90% of the electrode pairs fabricated had bridging SWCNTs.

Suspended CNT networks grown between pillar tops can be subsequently transferred to substrates for use in devices. Abrams et al. synthesized suspending SWCNT networks bridging Si pillars topped with catalyst, and then they transferred the whole SWCNT network to various substrates by pressing the pillars top down onto the substrate coated with deionized (DI) water.^[^ ^280^ ^]^ The water was necessary for successful transfer, due to capillary forces creating the adhesion to the target substrate. Seo et al. instead cast PDMS polymer onto suspended SWCNT networks, embedding them into a PDMS matrix for use as a strain sensor (Figure 14b).^[^ ^281^ ^]^ Raman spectroscopy can be used to characterize the location and chirality of the suspended SWCNTs before transfer, which is important for subsequent device fabrication.^[^ ^280^ ^]^ Suspended CNTs have a much higher Raman signal than surface‐contacting CNTs due to the stronger vibrations of the free tube.

### 6.2 Alignment of CNTs During Synthesis

Controlled electric fields can be used to direct CNT growth direction during thermal CVD. Electric fields induce a large dipole in CNTs parallel to their long axis. This allows the electric field to exert a force on the growing CNTs, aligning them parallel to the electric field and dominating the thermal fluctuations and gas flows.^[^ ^282^ ^]^ The alignment effect is similar to that used in DEP patterning (Section 3.3). Joselevich and Lieber demonstrated this method by growing SWCNTs aligned with an electric field from e‐beam lithography patterned lines of ferrihydrite nanoparticle growth catalysts.^[^ ^283^ ^]^ However, they found that shorter, semiconducting CNTs did not align well with the electric field. They proposed this was due to the three orders of magnitude smaller polarizability of semiconducting CNTs compared to metallic. This means that semiconducting CNTs ≤1.5 µm long have a maximum rotation energy that is < kT. Therefore, random thermal rotation dominates over electric field orientation. Metallic CNTs and longer semiconducting CNTs have a higher polarizability allowing orientation to align with the electric field to dominate. Electric field alignment is useful for fabricating CNTs bridging electrodes in devices, as an electric field can simply be applied across electrodes during CNT growth, aligning the growth direction to bridge the electrodes.^[^ ^231^, ^284^ ^]^ However, the electric fields must be well controlled around the electrodes.^[^ ^284a^ ^]^ Growing CNTs follow the field lines with the highest strength, which could direct the growing CNT to contact and strongly adhere to the substrate due to strong van der Waals interactions. Peng et al. bridged 10 µm gap electrode pairs with CNTs grown from Fe catalyst patterned in between the electrodes and patterned on the electrodes.

The direction of the CVD gas flow can also be used to direct the growth of the CNTs. Huang et al. used the gas flow direction to orientate the growth of SWCNTs from patterned nanoparticle catalysts during CVD.^[^ ^236^, ^285^ ^]^ If the substrate was heated quickly to 900 °C, they could produce highly aligned SWCNTs longer than 2 mm. They suggested that quick heating and fast growth were necessary to avoid the growing SWCNTs interacting and adhering to the substrate; similar to the effect in electric field‐oriented growth. They proposed a “kite‐mechanism” operates, where the quickly growing CNT is lifted up away from the substrate due to the intense convection flow from the temperature differences in the growth chamber.^[^ ^285^ ^]^ They could even fabricate SWCNTs grown in perpendicular directions on the same substrate, by growing the SWCNTs in two steps with perpendicular gas flow direction for each step. This method was improved by Jin et al. who instead in stark contrast used ultraslow gas flow to control the buoyancy effect much better.^[^ ^286^ ^]^ The slow gas flow provided much more stable laminar gas flows to direct the buoyant force on the CNTs, and prevented destabilizing secondary flows from occurring. They stated that the buoyant force originates from the gas temperature gradients near the substrate, creating convection flows away from the substrate and lifting the growing CNTs (Figure 14c). Using this method, they could fabricate very aligned arrays of >1 mm long CNTs in batch scale and could direct the growing CNTs over 3 µm gaps and over ≈10 µm high obstacles (Figure 14d,e). Papadopoulos and Omrane found that nanometer‐spaced neighboring catalyst islands help to maintain the direction and straight alignment of the growing CNTs to the gas flow direction.^[^ ^253^ ^]^ The growing CNTs quickly adhere strongly to close catalyst island neighbors from thermal rotations, and the CNTs growth direction is fixed. Gas flow can be used to easily fabricate CNT channels/wires across electrodes. Li et al. fabricated an all‐carbon electric device by using gas flow orientation to direct the growth of single SWCNTs across reduced graphene oxide electrode gaps.^[^ ^287^ ^]^ Electrode gaps >150 µm could be bridged, with close to ohmic contact between the SWCNT and reduced graphene oxide.

CVD growth of CNTs can be laterally directed by the crystallographic directions of the substrate.^[^ ^288^ ^]^ Substrates like sapphire and quartz that have a clean surface can direct the growth of CNTs along crystallographic lattice directions and atomic step edges (Figure 14f,g).^[^ ^288b,d^ ^]^ This is a reliable way to control global growth direction with a high degree of alignment and straightness, and this can be controlled to dominate other growth directors like gas flow or electric field direction. This technique is useful for easily growing evenly spaced, aligned CNT arrays,^[^ ^288^, ^289^ ^]^ with up to 160 CNTs µm^−1^ density^[^ ^289b^ ^]^ and up to ≈91% semiconducting purity.^[^ ^290^ ^]^ Su et al. first found that CVD‐grown CNTs can grow along crystal lattices, reporting growth of SWCNTs in three particular 60°‐separated directions on Si(111) surfaces and in two 90°‐separated directions on Si(100) surfaces.^[^ ^288a^ ^]^ They calculated that the energy minima were along these lattice directions, which was the cause of the SWCNTs’ directed growth. This lattice‐oriented growth is independent of the CNT chirality. Ismach et al. found that electrostatic interactions that occur on the atomic‐step are the strongest contributors to the orientation of growing CNTs to the crystal lattice direction (Figure 14h).^[^ ^288b^ ^]^ They modeled CNT‐step electrostatic interaction energies of ≈50 eV nm^−1^ compared to the van der Waals contribution of ≈2 eV nm^−1^. This was further confirmed by the growing CNTs not aligning to randomly oriented scratches on the miscut sapphire substrates. They also demonstrated that CNTs will even grow with kinks in order to match the atomic step edges of miscut sapphire substrates.^[^ ^288^, ^291^ ^]^ Atomic steps of miscut quartz substrates can be used too.^[^ ^289a^ ^]^ CNTs have been shown to grow aligned on flat faces, with the alignment following lines of the less electronegative atoms along the crystal lattice, although atomic step alignment may dominate.^[^ ^292^ ^]^ Ago et al. and Han et al. separately reported growth of SWCNTs along the rows of Al on flat faces of sapphire (Al_2_O_3_) substrates.^[^ ^288^, ^293^ ^]^ Other substrates like MgO or mica may also be used, which produce different growth directions and angles based on their lattice symmetries.^[^ ^294^ ^]^ Li et al. used single dot islands of Co nanoparticle catalyst to observe the alignment mechanism of growing CNTs along crystallographic directions.^[^ ^267^ ^]^ They found that CNTs initially grow randomly from the Co catalysts, and then bend to align themselves with the^[^ ^100^ ^]^ crystallographic direction of the quartz substrate, forming “sickle” shaped CNTs (Figure 14i). This is opposite to the theory suggested by Ding et al., where they suggested that CNTs initially grow aligned to the crystal direction and then bend to a random direction, forming “sickle” CNTs due to the growing tips’ collision with an unreacted catalyst nanoparticle.^[^ ^295^ ^]^ Li et al. results demonstrate that the initially random and then aligned growth is likely the accurate mechanism, as the origin of the CNT growth is well‐defined (the catalyst island). Previously grown CNTs can also be used to block the growth of subsequently grown perpendicular CNTs.^[^ ^296^ ^]^

Combinations of crystallographic‐oriented, electric field‐oriented, and gas flow‐oriented growth can be used simultaneously to produce more complex CNT architectures in the same CVD growth process.^[^ ^297^ ^]^ Ismach et al. reported SWCNT crossbar architectures fabricated by simultaneously growing SWCNTs along the atomic edge of a miscut sapphire substrate, and growing perpendicular SWCNTs via electric field orientation.^[^ ^297a^ ^]^ Directing gas flow perpendicular to atomic steps of the substrate can produce “serpentine” patterns of the CNTs on the substrate (Figure 14j).^[^ ^288^, ^297^ ^]^ Through this method, CNTs generally grow following the atomic step direction but can intermittently jump over a few atomic steps and make a “U‐turn” due to the gas flow direction. Highly periodic serpentine patterns can be formed using this technique with atomic step jumps of ≈0.5–8 µm (Figure 14k). Adjusting the gas flow rate affects the U‐turn diameter. They suggested a growth mechanism where the tip is growing suspended above the substrate, and the falling tail of the nanotube preferentially lands along the atomic step, periodically coiling around to a following step (Figure 14j). This gas flow‐oriented growth and then subsequent alignment when landing on the substrate are similar to that suggested by Li et al. when they grew CNTs from Co catalyst islands.^[^ ^267^ ^]^ The end of the nanotube lifting from the substrate while growing is also consistent with the growth mechanism proposed and investigated by Jin et al. when using ultraslow gas flow to direct CNT growth.^[^ ^286^ ^]^ Yao et al. slowed down the cooling step after CNT growth, slowing the CNTs landing on the substrate and allowing them to properly arrange into dense serpentines on the substrate.^[^ ^298^ ^]^ Using this process, they were able to fabricate dense CNT arrays for use in CNT devices with *I*_on_/*I*_off_ ratios up to 10^6^. The combined gas flow and substrate topography method of producing serpentine CNT patterns was investigated through simulations by Jawed et al.^[^ ^299^ ^]^ They developed continuum mechanics simulations based on the discrete elastic rods model to determine the pattern morphologies that result from experimental parameters like substrate structure/topography, gas flow, and CNT properties. The framework they developed from the simulations is a helpful guide for constructing electrical circuits of particular desired geometries using this technique.

## 7 Applications

### 7.1 ICs

**Figure 15:**
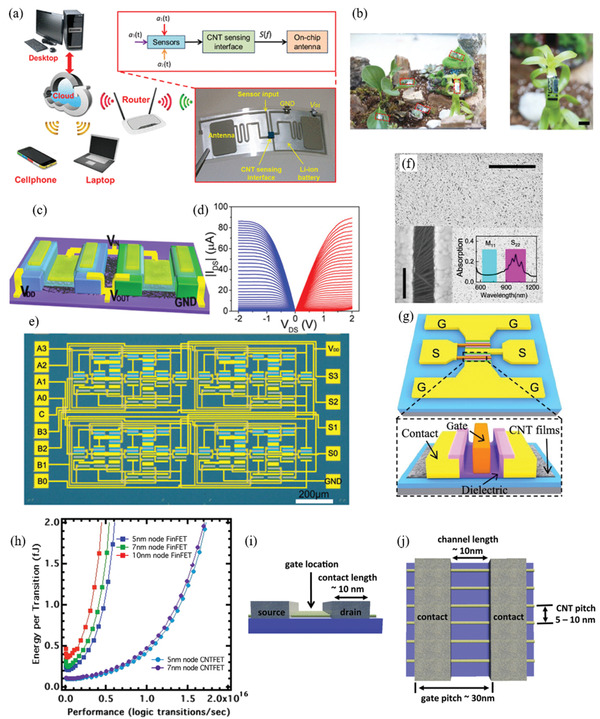
a) A universal wireless sensor interface system based on a CNT IC fabricated from patterned random CNT networks. Reproduced with permission.^[^ ^300f^ ^]^ Copyright 2019, American Chemical Society. b) Degradable environmental monitoring systems that can adhere to plants fabricated in high yield from patterned random CNT networks. Reproduced with permission.^[^ ^306^ ^]^ Copyright 2019, Wiley‐VCH. c) Schematic and d) output characteristics of CMOS CNTFETs fabricated from CNT networks patterned by oxidation etching. Both p‐FETs (blue) and n‐FETs (red) can be fabricated from CNT networks. e) Optical image of a CNT‐CMOS IC (4‐bit adder) fabricated from CNT networks (scale bar 200 µm). Reproduced with permission.^[^ ^216h^ ^]^ Copyright 2017, American Chemical Society. f) SEM image of random dense SWCNT network used for fabricating CNTFETs in CNT ICs (scale bar 2 µm). Insets are an SEM image of the SWCNT channel (scale bar 200 nm) and optical spectra of the semiconducting SWCNTs. g) Schematic of an RF CNTFET that can reach operating frequencies >100 GHz. Reproduced with permission.^[^ ^217a^ ^]^ Copyright 2019, American Chemical Society. h) Modeling of energy against performance for potential CNTFET logic technologies compared to current commercial Si FinFET technology. CNTFETs are projected to have >2‐fold increase to both energy and performance compared to Si FinFETs. i) Side and j) top view of optimized architecture of the 5 nm node CNTFET. Note that precise control of CNT position in the architecture is critical to achieve high performance. Nanoscale CNT patterning techniques are essential to realize these future technologies. Reproduced with permission.^[^ ^88c^ ^]^ Copyright 2014, American Chemical Society.

CNT‐based digital circuits of a variety of types have been fabricated recently, demonstrating the ability of CNTs to be integrating into many components of ICs with many potential benefits compared to conventional materials.^[^ ^78^, ^216^, ^300^ ^]^ CNT ICs are commonly fabricated from thin random networks of CNTs, which are subsequently oxidation etched into desired patterns.^[^ ^215^, ^301^ ^]^ Random networks often have poorer performance and poorer lateral resolution compared to aligned/dense networks, however they can be printed on flexible substrates for flexible devices and have a high manufacturing throughput due to their processability.^[^ ^78^, ^300^, ^301^, ^302^ ^]^ Printing nanoscale CNT ICs may also require an order of magnitude less energy compared to conventional nanoscale electronics manufacturing.^[^ ^303^ ^]^ This energy saving is critical as electronics become more ubiquitous. CNTFETs made from CNT random networks have been fabricated onto flexible substrates to assemble ICs including a 4‐bit adder composed of 140 CNTFETs with down to 2 V driving voltages,^[^ ^304^ ^]^ flexible ring oscillators with 5.7 ns stage delays,^[^ ^305^ ^]^ and recently even a flexible CNT wireless sensor interface circuit with flexible antenna, Li‐ion battery, and up to 1.98 GHz work frequency (**Figure** **15**a).^[^ ^300f^ ^]^ CNTFETs can also be fabricated as rugged, disposable, and biodegradable electronics for new IoT devices like environmental and agricultural monitoring systems (Figure 15b).^[^ ^306^ ^]^

Conversely, high‐performance CNT devices have also been fabricated using patterned semiconducting SWCNTs, demonstrating the ability of CNTs to compete and surpass conventional IC materials for future high‐performance electronics (Figure 15c–e).^[^ ^6^, ^216^, ^217^, ^300^, ^307^ ^]^ Initially, simple ICs made from single randomly deposited CNTs demonstrated the viability of CNTs to fabricate ICs including 3‐transistor circuits,^[^ ^308^ ^]^ and 5‐stage ring oscillators.^[^ ^309^ ^]^ The development of CNT‐based continued feverishly and has culminated in the recent demonstration of a fully operational 16‐bit CNT computer.^[^ ^4^, ^218^ ^]^ CNTFETs from CNT random network films have also been demonstrated to fabricate RF devices with operating frequencies greater than 100 GHz and gate lengths of 100–115 nm (Figure 15f,g).^[^ ^217^, ^310^ ^]^ This is greater than currently commercial CMOS RF devices, allowing these CNT devices to already be useable for current 5G millimeter‐wave wireless technology. However, CNT patterning methods to fabricate high‐performance CNT ICs are usually applied to rigid substrates and involve slower photolithography or EBL patterning steps. This means manufacturing costs and throughput is not drastically improved. Therefore, new CNT patterning techniques that can simultaneously achieve the desired properties of high lateral resolution, high density, alignment, chiral purity, high throughput all with arbitrary desired patterns will unlock the potential of CNTs for future ICs of all types.

IBM researchers have determined through modeling that aligned CNT densities of 125 CNTs µm^−1^, with semiconducting purities >99.9999% are required for CNT ICs to surpass the current high‐performance semiconductor materials.^[^ ^78^, ^88^ ^]^ Post‐synthesis CNT dispersion methods have produced CNT densities of up to 500 CNTs µm^−1^ (nearly full monolayer surface coverage),^[^ ^107^ ^]^ while maximum CNT density obtained during CNT synthesis has been 160 CNTs µm^−1^, demonstrating the possibility that post and pre‐synthesis patterning methods can produce adequate densities.^[^ ^289^, ^311^ ^]^ More importantly though a tight and controlled pitch of ≈5 nm between aligned CNTs has been projected to exhibit CNTFETs with better properties than current Si FinFET logic technologies (Figure 15h–j).^[^ ^88c^ ^]^ This adds to the manufacturing difficulty. Too high lateral density or too thick networks can result in strong tube–tube coupling or screening between proximate CNTs, severely degrading performance.^[^ ^107^, ^108^, ^300^ ^]^ Nanoscale CNT patterning techniques can effectively produce ICs from CNTFETs. Xiao et al. demonstrated inverter and ring oscillator circuits fabricated from DEP patterning of CNTs between electrodes,^[^ ^312^ ^]^ while Akinwande et al. demonstrated a two‐transistor amplifier fabricated using DEP to integrate CNTs into a standard CMOS VLSI system.^[^ ^313^ ^]^ In 2013, the Shulaker group at MIT demonstrated a functional CNT general computer using only CNTFETs.^[^ ^218^ ^]^ Then in 2019 they demonstrated an improved 16‐bit CNT‐based microprocessor with more than 14 000 CNTFETs that could run standard 32‐bit instructions.^[^ ^4^ ^]^ This demonstration proves the viability of CNTs for next‐generation high‐performance full‐scale devices. The latest CNT computer was fabricated through simple oxidation etching of deposited semiconducting SWCNTs through photoresist patterned with photolithography (Section 4.1). A major advantage of this method is the low‐temperature fabrication. This allows metallic interconnects to be fabricated above and below the CNTFETs and consequently improving congestion. Low‐temperature fabrication of CNTFETs also allows them to be integrated in back‐end‐of‐the‐line (BEOL) devices that are fabricated on top of and after the main front‐end‐of‐the‐line (FEOL) components that are high‐temperature sensitive. Fabricating CNT‐based components like power gating transistors at BEOL can free up valuable FEOL area on the substrate for additional components and reduce interconnect lengths.^[^ ^314^ ^]^ Although the patterning method employed is sufficient to produce a CNT computer, advanced nanoscale CNT patterning techniques could improve performance with better CNT alignment and nanoscale features/pitch for device scaling, as well as improve manufacturability. There is a lot of room for improvement still.

CNTs are also viable for memory technologies.^[^ ^315^ ^]^ Static random‐access memory arrays with competitive properties and operating voltages down to 300 mV were fabricated from solution‐deposited semiconducting CNT films that were patterned via photolithographically defined oxidation etching.^[^ ^316^ ^]^ The unique electromechanical properties of CNTs can also be exploited to realize innovative and new memory technologies including nonvolatile memories.^[^ ^315^, ^317^ ^]^ For example, recently Abbasi et al. fabricated a nanoelectromechanical bistable switch by using resist‐assisted DEP to deposit a nanoscale SWCNT bundle over recessed electrodes.^[^ ^317^ ^]^ The SWCNT bundle can switch between nonvolatile states by applying a voltage which pulls the initially suspending SWCNT bundle in contact with recessed electrode. Effective nanoscale CNT patterning is critical to increase storage density for these CNT memory devices.

### 7.2 FETs

**Figure 16:**
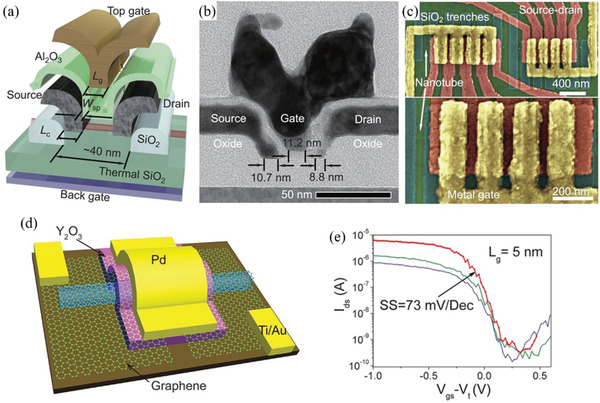
a) CNTFETs have been scaled down to 40 nm footprints, demonstrating high performances. b) TEM cross‐section and c) SEM images of the fabricated 40 nm CNTFETs. Reproduced with permission.^[^ ^319a^ ^]^ Copyright 2017, AAAS. d) CNTFETs from a single SWCNT and 5 nm gate lengths using graphene contacts have been fabricated and demonstrate excellent properties that approach the quantum limit. e) Transfer characteristics from typical 5 nm gate length devices. Reproduced with permission.^[^ ^319b^ ^]^ Copyright 2017, AAAS.

There has been a large amount of recent research exploring different fabrication methods and architectures for CNTFETs that use CNTs as the channel material.^[^ ^6^, ^78^, ^216^, ^300^, ^307^, ^318^ ^]^ There are three main advantages CNTFETs can have over conventional Si or crystalline semiconductor FETs. 1) CNTFETs can have superior electrical and mechanical properties and be scaled to smaller dimensions than possible with standard Si CMOS FETs (**Figure** **16**a–c).^[^ ^132^, ^319^ ^]^ CNTFETs have excellent intrinsic carrier mobility,^[^ ^91^, ^320^ ^]^ small subthreshold slopes that can potentially reach lower than the theoretical minimum of Si FETs (60 mV dec^−1^),^[^ ^321^ ^]^ high on‐state current density up to 900 µA µm^−1^,^[^ ^322^ ^]^ and high transconductance.^[^ ^219^, ^300^, ^307^ ^]^ These properties have the potential to allow Moore's Law to hold true, where conventional Si continues running into issues when scaling down. 2) CNTFETs patterned on flexible substrates can operate as flexible and/or transparent FETs.^[^ ^1^, ^6^, ^215^, ^219^, ^300^, ^301^, ^302^, ^304^, ^305^, ^323^ ^]^ This unlocks many applications for devices, including wearable and biocompatible electronics, transparent electronics, and more. 3) CNTFETs can potentially be fabricated cheaply, simply, and with high‐throughput, allowing electronics to be much more accessible and ubiquitous, which is very important for emerging technologies including IoT, edge computing, sensing, AI, and more.^[^ ^1^, ^5^, ^6^, ^301^, ^306^, ^324^ ^]^ However, these different advantages of CNTFETs are often found in different patterning techniques. High‐throughput CNT dispersion patterning is simple, cheap, and can be incorporated into flexible devices. The electrical properties can be poorer than conventional silicon/copper electronics, but still very good for printable or flexible electronics. Even recently, all‐printed SWCNT thin film transistors have been demonstrated with high *I*_on_/*I*_off_ ratio (10^5^–10^6^), small hysteresis, small subthreshold swing (≈70 mV dec^−1^), and low leakage currents.^[^ ^324c^ ^]^ Alternatively, patterned whole wafer‐aligned CNT films/arrays, DEP patterning, and CVD growth patterning can harness the exceptional electrical/mechanical qualities of individual CNTs. However, these patterning techniques are much slower, more complex, have additional limiting processing requirements (like high temperature), and are generally required to be on hard substrates (often silicon). Although for high‐performance electronics, these processing steps are not necessarily more complex than what is already used for CMOS chip manufacturing. Ultimately, patterning techniques that could precisely place individual, purified, high‐quality CNTs with nanometer precision and high throughput on a variety of substrates is the goal to unlock both the excellent performance and utility of CNTFETs.

Due to its quasi 1D electronic structure, superior gate control can be exerted on CNTFET channels.^[^ ^322^, ^325^ ^]^ Single CNT FETs with 5 nm gate lengths have been demonstrated to approach the quantum limit of FETs (Figure 16d,e), as the use of single electrons for switching operations.^[^ ^132^, ^319^ ^]^ Models have projected that using CNTFETs for high‐performance ICs can provide a potential ninefold energy‐delay product benefit, due to threefold faster processing using threefold less energy.^[^ ^326^ ^]^ While different gate structures for CNTFETs have been explored (back‐gate, top‐gate, gate‐all‐around), researchers have found that back‐gate structures may provide the most benefit, with >1.6 × additional energy‐delay‐product benefit for CNTFETs.^[^ ^319c^ ^]^ Another important advantage of back‐gate structures is that they can be aggressively scaled to 30 nm contacted gate pitch using CNT dispersion patterning/placement techniques.^[^ ^319a,c^ ^]^ These FET dimensions, which are suitable for the future sub‐3 nm technology nodes according to the International Technology Roadmap for Semiconductors (ITRS), are often not possible with other gate structures. This important fact demonstrates that CNT dispersion patterning techniques are strong candidates for fabricating future high‐performance transistors and ICs. Patterning CNT dispersions with substrate modification or templates can also enable self‐alignment when scaling down to sub‐micrometer and nanoscale resolution (Section 2.4.3), simplifying manufacturing. Although single CNT FETs have shown superior performance in experiments comparatively to conventional Si CMOS circuits, there is often very poor uniformity over multiple devices due to the difficulty of creating extremely homogenous CNTs and placing single CNTs at specific locations over whole wafers.^[^ ^300c^ ^]^ For this main reason, CNT networks/arrays are more common for realizing CNTFETs for large‐scale ICs. CNTFETs using CNT networks can also have degraded performance due to CNT inhomogeneity when channel lengths are submicron and the network is unaligned. Still, researchers have already been able to construct CNTFETs from CNT networks with remarkable and competitive properties.^[^ ^4^ ^]^

Many different nanoscale CNT patterning techniques have been used to fabricate high‐quality CNTFETs and some are presented in **Table** **1**. A common nanoscale CNT patterning technique used to produce high‐performance CNTFETs is oxidation etching.^[^ ^132^, ^319^, ^327^ ^]^ This patterning technique is used so that high‐quality, aligned CNTs or CNT arrays produced through other methods can be incorporated into the CNTFET to ensure high performance. The technique is also generally compatible and easy to incorporate in standard CMOS fabrication. However, it does rely on lithography and so is not a high throughput technique. Pre‐synthesis CNT patterning techniques are not as common for CNTFETs. This is due to the difficulty in patterning CNTs to bridge electrodes at particular positions. High temperature from CVD growth also inhibits its applicability. However, some groups have reported using these techniques to fabricate CNTFETs.^[^ ^328^ ^]^ Techniques that pattern CNTs from dispersions have been used very effectively to produce CNTFETs with some unique advantages. High‐quality CNTs can be synthesized and sorted prior to patterning rather than trying to simultaneously achieve high‐quality synthesis and precise positioning.^[^ ^78c^ ^]^ High processing temperatures are also not required, making them more compatible with CMOS and other fabrication processes. However, dispersing agents and CNT functionalizations can impair (sometimes significantly) the properties of the CNTFETs. Some techniques that pattern CNTs from dispersions can potentially be slow batch processes, limiting applicability for high‐throughput manufacturing.

**Table 1:** CNTFETs fabricated using nanoscale CNT patterning techniques

Year/group	CNT patterning technique	CNTFET type	Channel width	Gate length	Channel pitch	Notable characteristics
1998 Dekker^[^ ^336^ ^]^	Dispersion drop cast—No direct patterning	Si back‐gated single‐SWCNT on Si substrate	≈1.4 nm Single CNT	400 nm	–	First room temperature CNTFET. No CNT patterning technique used
1998 Avouris^[^ ^337^ ^]^	Dispersion drop cast—No direct patterning	Si back‐gated single‐SWCNT on Si substrate	< 5 nm Single CNT	1 µm	–	Room temperature CNTFET, transport dominated through holes (p‐type). No CNT patterning technique used
2000 Dai^[^ ^338^ ^]^	CVD catalyst patterning	Si back‐gated single‐SWCNT on Si substrate	≈2 nm Single CNT	3.5 µm	–	Doping of channel CNT from p‐type to n‐type, even making a p‐n junction along the same CNT
2002 Dai^[^ ^339^ ^]^	CVD catalyst patterning onto Mo electrodes^[^ ^279b^ ^]^	Top‐gated single‐SWCNT with 8 nm ZrO_2_ dielectric layer on Si substrate	≈1–3 nm Single CNT	≈2 µm	≈1–5 µm	Demonstration of high‐k dielectrics for CNTFETs. SS ∼ 70 mV dec^−1^, transconductance 6000 S m^−1^, carrier mobility 3000 cm^2^ V^−1^ s^−1^
2002 Avouris^[^ ^340^ ^]^	Spin casting CNT dispersion—No direct patterning	Top‐gated single‐SWCNT with 10–20 nm SiO_2_ dielectric layer on Si substrate	≈1–3 nm Single CNT	260 nm	≈5 µm	SS 130 mV dec^−1^, transconductance 2321 S m^−1^
2003 Braun^[^ ^195^ ^]^	DNA‐templating to self‐assemble SWCNTs between electrodes	Si back‐gated single or bundle SWCNTs on Si substrate	< 100 nm	150–300 nm	–	First DNA‐templated CNTFETs. P‐type behavior, although poor properties due to presence of metallic CNTs
2003 Krupke^[^ ^156^ ^]^	AC DEP of SWCNT in DMF	Si back‐gated bundle SWCNT on Si substrate	3–4 nm CNT bundle	400 nm	10 µm	First demonstration of DEP assembled CNTFETs 70% device yield over 16 devices
2003 IBM^[^ ^341^ ^]^	Dispersion drop cast–No direct patterning	Si back‐gated single or bundle SWCNTs on Si substrate	≈1.4 nm or more Single CNT/bundle	≈300 nm	–	Optical emission in IR region demonstrated from the CNTFET
2004 Avouris IBM^[^ ^321a^ ^]^	Spin casting CNT dispersion—No direct patterning	Si double‐back‐gated single SWCNTs on Si substrate	1–2 nm Single CNT	200 nm	–	Excellent SS of ≈40 mV dec^−1^ due to bandpass‐filter‐like operation of band‐to‐band tunneling device
2004 Fuhrer^[^ ^320^ ^]^	CVD grown from randomly deposited ferric catalyst. Gas flow alignment	Si back‐gated long single SWCNT on Si substrate	≈4–5 nm Single CNT	≈325 µm	–	Extremely high field‐effect mobility 79 000 cm^2^ V^−1^ s^−1^, estimated intrinsic mobility >100 000 cm^2^ V^−1^ s^−1^
2004 Dai^[^ ^342^ ^]^	No direct patterning	Top‐gated single‐SWCNT with 8 nm HfO_2_ dielectric layer on Si substrate	≈1.7 nm Single CNT	50 nm	–	Near‐ballistic conductance saturation current ≈25 µA *I*_on_/*I*_off_ > 10^3^ at *V*_DS_ = 0.3 V SS ∼ 110 mV dec^−1^
2004 Li^[^ ^330^ ^]^	AC DEP of SWCNT in IPA	Si back‐gated SWCNT bundle on Si substrate	≈35 nm CNT bundle	3.6 µm	–	60% yield of bridged electrodes *I*_on_/*I*_off_ 7 × 10^5^ at *V*_DS_ = −0.15 V
2005 Derycke^[^ ^125^ ^]^	CNT dispersion patterning by SAM‐patterned substrate	Si back‐gated single SWCNT on Si substrate	∼1‐3 nm Single CNT	100‐500 nm	–	SAM chemistry can be tuned to selectively dope CNT and decrease Schottky barrier
2005 Jiao^[^ ^185^ ^]^	AC DEP of aqueous SWCNT	Si back‐gated single SWCNT on Si substrate	≈1–3 nm Single CNT	500 nm	–	*I*_on_/*I*_off_ ∼ 10^5^ 26% single CNT device yield
2005 Seidel, Infineon Technologies^[^ ^328a^ ^]^	Patterned CVD growth catalyst via conventional photolithography	Si back‐gated single SWCNT on Si substrate	≈0.7–1.1 nm Single CNT	18–20 nm	–	Nanoscale channel length. Transconductance 4000 S m^−1^ current density > 10^9^ A cm^−2^
2005 Avouris^[^ ^343^ ^]^	Patterning technique not described	Al fully gated single SWCNT on Si substrate	≈1.8 nm Single CNT	40 nm	–	delay time 19 ps µm^−1^ *I*_on_/*I*_off_ > 10^5^ SS 80–120 mV dec^−1^
2005 Zhang^[^ ^147c^ ^]^	AC DEP of aqueous SWCNT	Si back‐gated single SWCNT on Si substrate	≈1.1 nm Single CNT	1 µm	–	*I*_on_/*I*_off_ ∼ 10^8^
2006 Hong^[^ ^110^, ^118^ ^]^	CNT dispersion patterning by SAM‐patterned substrate	Au top‐gated SWCNT network on Si substrate	3 µm for wafer‐scale Also single CNT	4 µm	> 1 µm	First successful demonstration of wafer‐scale CNT dispersion patterning of CNTFETs ≈99% yield of CNT networks connecting 64 000 electrode pairs *I*_on_/*I*_off_ 10^6^
2006 Avouris^[^ ^118f^ ^]^	CNT dispersion patterning by hydroxamic acid functionalized CNTs adhering to metal oxide patterns	Si back‐gated SWCNT network on Si substrate	300 nm	400 nm	–	*I*_on_/*I*_off_ > 10^6^ SS ∼ 115 mV dec^−1^ at *V*_DS_ = −0.2 V 57% working device yield
2006 Dai^[^ ^328c^ ^]^	Patterned CVD growth catalyst via conventional photolithography	Top‐gated single‐SWCNT with 3 nm HfO_2_ dielectric layer on Si substrate	≈1.2 nm Single CNT	100 nm	–	SS 60 mV dec^−1^ at room temp. SS 50 mV dec^−1^ in band‐to‐band tunneling regime
2006 Teo^[^ ^328b^ ^]^	Patterned CVD growth catalyst via conventional photolithography	Top‐gated single‐SWCNT with 5 nm TiO_2_ dielectric layer on Si substrate	≈1.3 nm Single CNT	350 nm	–	SS 67–70 mV dec^−1^ *I*_on_/*I*_off_ ∼ 10^4^ Transconductance 1000 S m^−1^ at *V*_DS_ = 0.2 V
2007 Zhang^[^ ^136^ ^]^	Channel template CNT dispersion patterning and wafer transfer	Si back‐gated SWCNT bundle on Si substrate	20 nm CNT bundle	15 µm	–	
2007 Xiao^[^ ^344^ ^]^	AC DEP of SWCNT in DMF/toluene	Al backside gated SWCNTs on Si substrate	500 nm	800 nm	–	Semiconductor electrodes
2007 Tulevski, Avouris^[^ ^110f^ ^]^	CNT dispersion patterning by hydroxamic acid functionalized CNTs adhering to metal oxide patterns	Si back‐gated few‐SWCNTs with HfO_2_ dielectric layer on Si substrate	800 nm Single CNT	300 nm	–	90% functional device yield from whole wafer Alignment due to nanoscale confinement *I*_on_/*I*_off_ ∼ 10^7^
2007 Krupke^[^ ^2a^ ^]^	Floating‐potential AC DEP of aqueous SWCNTs	Si back‐gated single SWCNT on Si substrate	≈1–3 nm Single CNT	800 nm	≈4 µm	device density >10^6^ cm^−2^, 90% single CNT yield ULSI compatible *I*_on_/*I*_off_ > 10^3^
2008 Khondaker^[^ ^345^ ^]^	AC DEP of SWCNTs in dichloroethane over 115 devices simultaneously	Al local back‐gated single SWCNT on Si substrate	Single or bundle SWCNTs	100 nm	–	35% functional device yield SS 170 mV dec^−1^ *I*_on_/*I*_off_ > 10^4^ Transconductance 200 S m^−1^
2008 Zhang, Peng^[^ ^346^ ^]^	Not mentioned	Top‐gated single‐SWCNT with HfO_2_ dielectric layer on Si substrate	1.5 nm Single CNT	120 nm	–	SS 100 mV dec^−1^ Gate delay time 0.86 ps electron mobility 4650 cm^2^ V^−1^ s^−1^ near ballistic conduction
2009 Weitz^[^ ^347^ ^]^	CNT dispersion patterning by SAM‐patterned substrate	Al local back‐gated single SWCNT on Si substrate	1.5 nm Single CNT	100 nm	–	SS 68 mV dec^−1^ *I*_on_/*I*_off_ 10^7^ Transconductance 40 S m^−1^ no degradation after more than 300 days
2010 Franklin^[^ ^348^ ^]^	CVD growth catalyst patterned by photolith. on aligning quartz substrate and transferred	Pd local back‐gated single SWCNT with 10 nm HfO_2_ dielectric on Si substrate	1.1–1.3 nm Single CNT	15 nm	–	Demonstrates absence of short‐channel effects conductance 0.7 G_0_ transconductance 2666 S m^−1^ *I*_on_/*I*_off_ 10^5^
2010 Khondaker^[^ ^331^ ^]^	AC DEP of aqueous surfactant‐free SWCNTs	Si back‐gated single SWCNT on Si substrate	1.5 nm	1 µm	–	mobility 1380 cm^2^ V^−1^ s^−1^ Max on‐state conductance 6 *μ*S
2010 Krupke Vijayaraghavan^[^ ^158^ ^]^	AC DEP of aqueous semiconducting purified SWCNTs	Si back‐gated few and single SWCNT on Si substrate	1–3 SWCNTs	500 nm	2–3 µm	Demonstration of using semiconducting purified SWCNTs with DEP *I*_on_/*I*_off_ 10^5^
2011 Cao^[^ ^333a^ ^]^	Photoresist‐assisted floating‐potential AC DEP of aqueous SWCNTs	Si back‐gated single SWCNT suspended over Si substrate	50 nm Single CNT	2 µm	> 1 µm	SS 130 mV dec^−1^ *I*_on_/*I*_off_ 10^5^
2011 Krupke Vijayaraghavan^[^ ^349^ ^]^	Floating‐potential AC DEP of aqueous semiconducting SWCNTs	Si back‐gated single SWCNT suspended over Si substrate	1–3 SWCNTs	700 nm	≈1 µm	98% of 100 devices assembled simultaneously *I*_on_/*I*_off_ > 10^5^ Potential ambipolar transport
2011 Khondaker^[^ ^350^ ^]^	Floating‐potential AC DEP of semiconducting SWCNTs	Al local back‐gated single SWCNT on Si substrate	≈1.6 nm Single CNT	100 nm	–	99% functional device yield for 70 devices 20% single CNT yield SS 140 mV dec^−1^ *I*_on_/*I*_off_ ∼ 10^6^
2012 Cao^[^ ^333b^ ^]^	Photoresist‐assisted AC DEP of aqueous SWCNTs	Lateral gated single SWCNT suspended over Si substrate	100 nm Single CNT	800 nm	500 nm	85% single CNT yield over 50 devices *I*_on_/*I*_off_ ∼ 10^7^ SS 133 mV dec^−1^ min current 10^−14^ A
2012 Franklin^[^ ^132b^ ^]^	CVD growth catalyst patterned by photolith. on aligning quartz substrate and transferred. Then oxidation etching of aligned CNTs	W local back‐gated single SWCNT on Si substrate	≈1.3 nm Single CNT	9 nm	–	first sub‐10 nm CNTFET High diameter‐normalized current density 2410 µA µm^−1^ at 0.5 V SS 94 mV dec^−1^
2012 Khondaker^[^ ^351^ ^]^	AC DEP of aqueous semiconducting SWCNTs	Al local back‐gated single SWCNT on Si substrate	1–5 SWCNTs	100 nm	≈20 µm	90% assembly yield with 90% FET behavior *I*_on_/*I*_off_ ∼ 10^6^ *I*_on_ 3 µS Mobility 210 cm^2^ V^−1^ s^−1^
2012 Park^[^ ^2b^ ^]^	CNT dispersion patterning by SAM‐patterned substrate	Si back‐gated single SWCNT on Si substrate	70 nm Single CNT in each trench	100 nm	200 nm *x* axis 500 nm *y* axis	10^9^ cm^−2^ CNTFET density 78–90% single CNT yield from >10 000 devices Average SS 265 mV dec^−1^ from 7066 devices Alignment due to nanoscale confinement
2012 Steiner, Avouris^[^ ^352^ ^]^	AC DEP of semiconducting purified SWCNTs	Local back‐gated SWCNT network with 10 nm HfO_2_ dielectric on Si substrate	2 µm	100 nm	≈20 nm between adjacent CNTs	Aligned CNT densities 50 µm^−1^ Intrinsic current gain and power gain cut‐off frequencies of 153 and 30 GHz
2013 Franklin^[^ ^327a^ ^]^	CVD growth catalyst patterned by photolith. on aligning quartz substrate and transferred. Then oxidation etching of aligned CNTs	Gate‐all‐around single SWCNT with HfO_2_ dielectric on Si substrate	≈1.2 nm Single CNT	20 nm	≈66–1000 nm between adjacent CNTs	Aligned CNT densities 1–15 µm^−1^ Low interference between adjacent CNTs SS 99 mV dec^−1^ *I*_on_/*I*_off_ > 10^4^
2014 Q Cao^[^ ^334^ ^]^	Fringing‐field AC DEP of aqueous semiconducting SWCNTs	Si back‐gated SWCNT arrays on Si substrate	1 µm	100 nm	21 ± 6 nm	Good lateral resolution Self‐limiting pitch 50 aligned CNTs µm^−1^ High transconductance >50 S m^−1^ *I*_on_/*I*_off_ ∼ 10^3^
2014 Dai^[^ ^139c^ ^]^	Semiconducting purified SWCNT dispersion patterning by photoresist templated substrate	Si back‐gated SWCNT bundles on Si substrate	800 nm	130 nm	100–200 nm between adjacent CNT bundles	97% channel length filled *I*_on_/*I*_off_ ∼ 3 × 10^5^ Current density 80 µA µm^−1^
2015 Cao^[^ ^2c^ ^]^	Photoresist‐assisted AC DEP of aqueous SWCNTs	Lateral gated single SWCNT suspended over Si substrate	30 nm Single CNT	≈1 µm	≈1 µm	>10^6^ CNTFETs cm^−2^ 80% single CNT yield *I*_on_/*I*_off_ ∼ 10^4^ SS 220 mV dec^−1^ *I*_off_ at fA regime
2015 Wong/Bao^[^ ^110j^ ^]^	SWCNT dispersion sheared over nanoscale SAM patterned wafer	Si back‐gated SWCNT networks on Si substrate	0.5–10 µm	0.4–1 µm	1–20 µm	Large‐area scalability, and potentially high throughput Alignment during patterning *I*_on_ 10.08 µA µm^−1^ *I*_on_/*I*_off_ ∼ 10^4–7^
2016 Hennrich^[^ ^332^ ^]^	AC DEP of aqueous semiconducting SWCNTs	Si back‐gated aligned semiconducting SWCNT array	1 µm	800 nm	–	>99.7% semiconducting SWCNTs *I*_on_/*I*_off_ ∼ 2 × 10^8^ Hole mobility 297 cm^2^ V^−1^ s^−1^
2016 Lee, Kim^[^ ^2d^ ^]^	Dip pen nanolithography of CNT dispersion ink	Si back‐gated SWCNT network on Si substrate	≈2 µm	≈50 µm	–	Directly patterned CNTFET channels from CNT ink Hole mobility 4.68 cm^2^ V^−1^ s^−1^
2017 Cao^[^ ^319a^ ^]^	Aligned dense semiconducting SWCNT film patterned by EBL and oxidation etching	Si back‐gated aligned semiconducting SWCNT array with HfO_2_ dielectric layer on Si substrate	≈50 nm CNT array	≈11 nm	≈50 nm	40 nm total footprint matching that recommended by ITRS, generally better performance than Si technologies *I*_on_/*I*_off_ ∼ 10^3^ current density 800 µA µm^−1^
2017 Loi^[^ ^134^ ^]^	Thiol‐functionalized CNT dispersion patterning by dip‐coating and adhesion to Au electrodes	Si back‐gated SWCNT on Si substrate	300 nm Single CNT	300 nm	‐	≈100% functioning yield from 50 devices *I*_on_/*I*_off_ ∼ 10^4^
2017 Han^[^ ^78a^ ^]^	Semiconducting CNT dispersion patterning by SAM‐patterned substrate	W embed‐gate aligned semiconducting SWCNT network with HfO_2_ dielectric layer on Si substrate	100 nm	152 nm	200 nm	stage switching frequency 2.82 GHz 100% yield over 192 devices CMOS manufacture compatible
2018 Engel^[^ ^164c^ ^]^	AC DEP of semiconducting SWCNTs using removable graphene electrodes	Local back‐gated SWCNT array on Si substrate	≈1 µm	40 nm	<100 nm	Demonstration of high density, nanometer scalable fabrication of CNTFETs using DEP, and removable electrodes 50 aligned CNTs µm^−1^ *I*_on_/*I*_off_ ∼ 10^5^
2019 Pitner^[^ ^327b^ ^]^	EBL and oxidation etching patterning of aligned individualized CNT array	Pt local back‐gated single SWCNT with HfO_2_ dielectric on Si substrate	Single SWCNT	50 nm	1 µm	≈10 nm side‐contact lengths possible with minimal resistance, 15 nm shorter than current Si CMOS 7 nm node
2019 Shulaker^[^ ^319c^ ^]^	EBL and oxidation etching patterning of random CNT network	Pt local back‐gated single SWCNT with HfO_2_ dielectric on Si substrate	<200 nm	10 nm	–	Current smallest contacted gate pitch 30 nm.
2020 Flavel^[^ ^353^ ^]^	AC DEP of aqueous semiconducting SWCNTs	Si back‐gated aligned semiconducting SWCNT array	1 µm	600 nm	–	(14,6) ‐chirality purity of up to 80% SWCNTs *I*_on_/*I*_off_ ∼ 6 × 10^6^ Hole mobility 10 ± 5 cm^2^ V^−1^ s^−1^

Park et al. manufactured and tested up to 7066 single‐SWCNT transistors on a single chip by depositing a semiconducting SWCNT dispersion on a SAM patterned substrate.^[^ ^2b^ ^]^ The surfactant‐functionalized SWCNTs were selectively adhered to the pyridinium‐ended SAM, which itself was bonded to patterned HfO_2_. Individual SWCNTs could be deposited and self‐aligned at 90% yield on 100 nm wide SAM patterns with 300 nm pitch. The alignment occurred spontaneously due to nanoscale confinement (Section 2.4.3). Ultimate transistor densities achieved by them were >10^8^ transistors cm^−2^, demonstrating scalability of the technique to nanoscale features. The measured average threshold voltage of −0.04 ± 0.44 V and average subthreshold swing of 265.2 ± 120.8 mV dec^−1^ were measured over 7066 devices. This method of using nanoscale SAM‐patterned substrates to deposit purified CNT dispersions was extended by Han et al., demonstrating a CNT ring oscillator with stage switching frequency of 2.82 GHz from simultaneously fabricated CNTFETs.^[^ ^78a^ ^]^ They used SAM patterns of 100 nm width and 200 nm pitch to adhere one or few semiconducting SWCNTs, creating 192 devices with 100% functional yield. Park et al. blade sheared SWCNT dispersions over whole SAM patterned wafers to fabricate nanoscale CNTFET channels. This CNT dispersion patterning technique shows potential for high‐throughput manufacturing and possibly R2R processability in fabricating nanoscale CNTFETs (Figure 5c,d).^[^ ^110j^ ^]^ DPN has also been used to fabricate CNTFETs, although the technique can have quite slow throughput.^[^ ^2d^ ^]^

CNTFETs have also been fabricated via DEP patterning, and this technique presents unique advantages. The DEP‐deposited CNTs are highly aligned, decreasing the resistance and increasing the electrical quality of the CNTFETs. Individual CNTs can also be easily patterned and used as the channel between electrodes, which is very difficult for other patterning techniques to achieve. Krupke et al. fabricated the first basic CNTFETs using DEP in 2003 with SWCNTs deposited over 16 Ag electrode pairs with 400 nm gaps.^[^ ^156^ ^]^ The yield of SWCNT connected electrodes was only ≈70%. They used high‐voltage pulses to destroy deposited metallic SWCNTs through electrical breakdown, establishing semiconducting behavior in the CNTFET with the remaining semiconducting SWCNTs. This semiconductor enrichment technique is common for CNTFETs.^[^ ^329^ ^]^ They demonstrated that Schottky‐barrier CNTFETs with p or n type could be fabricated using DEP. Li et al. were first to demonstrate practical CNTFETs fabricated in ambient conditions, achieving a good *I*_on_/*I*_off_ of 7 × 10^5^ and maximum transconductance of 0.19 µS at 1 V.^[^ ^330^ ^]^ They also implemented high voltage pulses to destroy metallic SWCNTs and improve performance. Zhang et al. were able to achieve a record *I*_on_/*I*_off_ of ≈10^8^ using AC DEP to deposit single SWCNTs for CNTFETs, which is very high for any CNTFETs.^[^ ^147c^ ^]^ Using surfactant‐free SWCNT dispersions, Stokes and Khondaker fabricated SWCNT FETs using AC DEP and achieved field effect mobility of 1380 cm^2^ V^−1^, near the theoretical performance limit, with on‐state conductance of 6 µS and >10^4^ *I*_on_/*I*_off_.^[^ ^331^ ^]^ The improved mobility demonstrates the importance of a clean and nonfunctionalized SWCNT for device performance. Hennrich et al. used DEP to pattern and align >99.7% semiconducting‐enriched SWCNTs for CNTFETs.^[^ ^332^ ^]^ They were able to achieve a remarkable *I*_on_/*I*_off_ of 2 × 10^8^ and hole mobility of 297 cm^2^ V^−1^ s^−1^ even with an SWCNT network due to the very high semiconducting purity.

**Figure 17:**
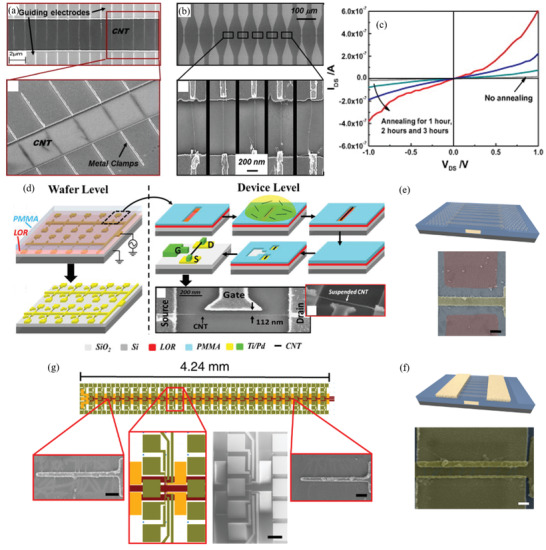
a) SEM images and enlargements of SWCNTs bridging electrode pairs fabricated via photoresist‐assisted DEP CNT patterning, and b) CNTFETs fabricated with the same technique. c) Drain *I*–*V* curves of semiconducting SWCNT arrays fabricated from the DEP patterning technique. Reproduced with permission.^[^ ^333b^ ^]^ Copyright 2011, Elsevier. d) Process of photoresist‐assisted DEP CNT patterning for fabrication of CNTFETs over whole wafers. SEM images show the suspended SWCNTs between electrodes as CNTFETs with lateral gate structure. Reproduced with permission.^[^ ^2c^ ^]^ Copyright 2015, American Chemical Society. e) Illustration and false color SEM image of DEP‐patterned SWCNTs bridging graphene electrodes (red) with embedded metal gate electrode (yellow) for fabrication of CNTFETs (scale bar 200 nm). f) Illustration and false color SEM image of the CNTFETs after removal of the graphene electrodes and deposition of top contact source/drain electrodes (scale bar 100 nm). g) SEM image and enlargements of large‐scale fabrication of many connected CNTFETs using AC DEP patterning and removable graphene electrodes (inset scale bars left to right 200 nm, 50 µm, 200 nm). Reproduced under the terms of the CC BY 4.0 license.^[^ ^164c^ ^]^ Copyright 2018, The Authors, published by Nature.

Photoresist‐assisted AC DEP was used by Cao et al. to create arrays of CNTFETs on wafers (**Figure** **17**a–c). They used trenches in the photoresist to help guide the assembly of CNTs down to nm resolution, and also allowed for suspended CNT structures.^[^ ^2^, ^333^ ^]^ A sample wafer consisting of 50 CNTFETs was fabricated, with minimum CNT period of ≈500 nm with sub‐50 nm error (Figure 17b,c).^[^ ^333b^ ^]^ The yield obtained of a single CNT bridging an electrode pair was very high ≈85%. They reported impressive *I*_on_/*I*_off_ ratios of ≈10^7^ subthreshold swing of 133 mV dec^−1^, and down to 10^−14^ A minimum current. Wafer‐level patterning of CNTFETs was achieved using the same technique, with >10^6^ CNTFETs cm^−2^ fabricated on the wafers (Figure 17d).^[^ ^2c^ ^]^ However, the quality was slightly lower, with ≈80% yield of single CNTs suspended between electrode pairs, *I*_on_/*I*_off_ ratio of ≈10^4^, and subthreshold swing of 220 mV dec^−1^. They did however achieve *I*_off_ at the femtoamp (fA) regime, which is important for fabricating low power consumption devices. Using AC DEP, Cao et al. were able to achieve even greater pitch resolution/control with average intertube pitch up to 21 ± 6 nm, allowing a patterning density of 50 CNTs µm^−1^ along one dimension, and high on‐current density of 43 µA µm^−1^ for subsequently fabricated CNTFETs.^[^ ^334^ ^]^ The density of aligned CNTs is higher than previously reported.^[^ ^335^ ^]^ The key was fabricating very high electric fringing‐field on the edge of gold electrodes, with the silicon wafer substrate as the counter electrode and silicon oxide layer as insulating interlayer. The high electric field allows the dielectrophoretic force to dominate, resulting in a very straight alignment. Also, similar to the effects observed by Chung et al.^[^ ^162a^ ^]^ and Vijayaraghavan et al.,^[^ ^2a^ ^]^ single CNTs were deposited in a self‐limiting process, where placement of one CNT in area of the electrode changed the E‐field to prevent additional CNT deposition. These two effects of the strong fringing‐field allowed single CNTs to be deposited along the electrode with high resolution and uniform spacing/pitch.

Engel et al. recently demonstrated high‐quality CNTFETs fabricated via AC DEP using removable graphene electrodes (Figure 17e,f).^[^ ^164c^ ^]^ The graphene electrodes can be removed after CNT deposition, adding flexibility to the fabrication process of CNTFETs for ICs. The graphene electrodes are also extremely thin, allowing the deposited CNTs to lie flatter on the substrate, improving electrical properties. From this, they could achieve <100 nm resolution fabrication of CNTFETs with up to 50 CNT µm^−1^ all highly aligned over whole wafers and high *I*_on_/*I*_off_ of 10^5^ (Figure 17g). This advancement demonstrates the ability of DEP CNT patterning to fabricate easily high‐performance next‐generation transistors. It should be noted that this is still not a high‐throughput technique, and so other CNT patterning techniques should be explored if high throughput is required.

### 7.3 Interconnects

CNT interconnects have been modeled and compared to conventional copper interconnects. Assuming high density of mostly metallic CNTs, CNT interconnects are expected to outperform conventional copper local, intermediate, and global interconnects.^[^ ^318^, ^354^ ^]^ As IC components continue to scale down to the deep nanoscale regime, conventional Cu interconnects suffer from sharply increasing resistance from grain boundary scattering, surface scattering, and the high resistance diffusion layer surrounding the Cu.^[^ ^354e^ ^]^ The increasing resistance consequently results in much higher self‐heating of the interconnects. CNTs have been proposed by researchers and the ITRS as an alternative interconnect material to deal with these problems in the deep nanoscale regime. Metallic SWCNTs have demonstrated high current capacities up to 10^9^ A cm^−2^ compared to ≈10^6^ A cm^−2^ for Cu, which is one of the most important properties for interconnects.^[^ ^354e^ ^]^ CNTs also have an approximately tenfold higher thermal conductivity compared to Cu; ≈3500 W m^−1^ K^−1^ compared to ≈385 W m^−1^ K^−1^, respectively.^[^ ^355^ ^]^ This is critical for heat management in the ICs, as heat can severely impact performance and degrade the ICs due to electromigration or thermal damage. Additionally, CNT interconnects do not experience electromigration unlike Cu. This is due to CNT activation energy for bonds is much higher 3.6 eV compared to Cu activation energy of ≈1 eV.^[^ ^355^ ^]^ Despite all these advantages though, CNT interconnects are much harder to fabricate, resulting in experimentally fabricated CNT interconnects displaying poorer properties than models suggest. This is generally because the purity and length of the CNTs are difficult to control well. CNT density in the interconnects is also often too low, with ITRS suggesting a minimum density of 10^13^ CNTs cm^−2^.^[^ ^354e^ ^]^ However, some publications have demonstrated it is possible to obtain densities of > 10^13^ CNTs cm^−2^ from CVD synthesis for use as CNT interconnects.^[^ ^356^ ^]^ As mentioned earlier, very high densities of ≈2.5 × 10^13^ CNTs cm^−2^ have been achieved using dispersion deposited techniques,^[^ ^107^ ^]^ however these have not yet been explored for creating interconnects.^[^ ^354d^ ^]^ Recently, composites of CNTs and Cu have been synthesized that can take advantage of the excellent properties of the CNTs without requiring as high CNT densities.^[^ ^357^ ^]^

CVD growth of CNTs on patterned catalyst is a useful and common synthesis route for CNT interconnects in devices. The robust attachment and excellent electric contact between CNT and substrate surface possible through this technique is helpful for interconnect applications. However, the high temperature required for CVD growth of the CNTs limits the usability of this technique in device fabrication, as materials already in the device before the CVD process must be resistant to the very high temperatures. The ballistic conductance for CNTs can also vastly improve performance for interconnects, especially for vertical interconnects. Already, researchers have shown that the typical length for the vertical interconnects is under the electron mean free path for the CNT interconnects, ensuring electrical and thermal ballistic conductance through the vertical interconnects and improving performance and reliability compared to Cu or W.^[^ ^354^, ^358^ ^]^ The higher thermal conductivity of CNTs compared to Cu also make CNTs suited for high‐aspect‐ratio vertical interconnects, a key component for the developing 3D integrated circuits technology.^[^ ^354^, ^359^ ^]^ Contact resistances between the CNTs and metal contacts can significantly contribute to the resistance through interconnects. Some groups have addressed this issue by contacting the CNT interconnects with graphene instead to create all carbon 3D interconnects.^[^ ^360^ ^]^ Generally, vertical CNTs interconnects are grown directly from horizontal graphene interconnects, which produce full direct contact with the graphene interconnects and minimal contact resistance.^[^ ^361^ ^]^ Graphene can also be transferred directly onto vertical CNT interconnects as a top contact graphene layer.^[^ ^362^ ^]^

**Figure 18:**
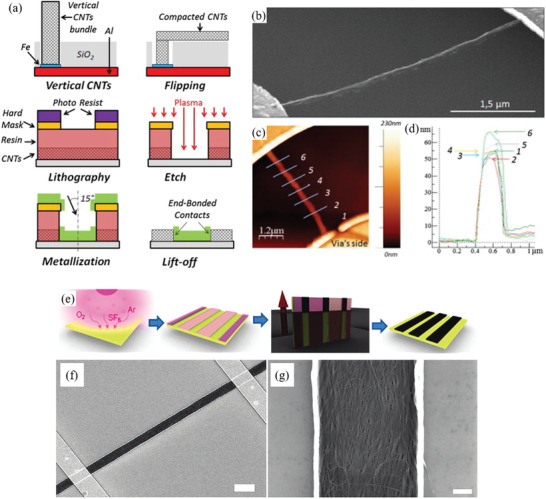
a) Process for fabricating end‐contacted horizontal CNT interconnects via growth catalyst patterning, CNT compaction, and oxidation etching. b) SEM image, c) AFM image, and d) AFM line profile of an end‐contacted horizontal CNT interconnect fabricated with the technique. Reproduced with permission.^[^ ^356c^ ^]^ Copyright 2013, Elsevier. e) Method for nanoscale template‐based CNT fluidic assembly of high density and aligned SWCNT interconnect structures. f) SEM images of the fabricated SWCNT interconnects and g) enlargement of the morphology. Scale bars 2 µm and 200 nm, respectively. Reproduced with permission.^[^ ^110h^ ^]^ Copyright 2009, American Chemical Society.

Zexiang et al. employed an interesting multi‐layered catalyst structure to create vertical CNT interconnects between bottom and top electrodes.^[^ ^363^ ^]^ Layers of catalysts were placed in between a Si substrate and a top Cu electrode layer. Microwave PECVD was then applied to these stacks oriented vertically and horizontally, and up to 5 µm long CNTs were grown in between and perpendicular to the Si and Cu electrode layers, pushing the Cu electrode layer up from the Si substrate. Individual MWCNTs were measured to have a contact resistance of ≈14.7 kΩ, approaching the quantum limit conductance. Awano et al. reported a reliable technique to fabricate vertical interconnects that is compatible for CMOS in VLSI technologies.^[^ ^364^ ^]^ One key issue with CVD growing CNTs for the vertical interconnects is that the temperature must stay below a threshold (usually 450 °C) to avoid damaging the already modified substrate. They were able to vertically grow MWCNTs through a low temperature 365–450 °C CVD process using Co catalyst nanoparticles deposited in holes with down to 40 nm diameter. After CVD growth, chemical mechanical polishing was used to remove excess CNTs and level the interconnects ends, and then top contacts could be patterned on top. The CNT vertical interconnects could maintain a 5.0 × 10^6^ A cm^−2^ current density at 105 °C for 100 h, with resistance similar to that of already used tungsten plugs. Similar demonstrations of CNT vertical interconnects were fabricated by other groups, but with not as beneficial properties.^[^ ^365^ ^]^ Chiodarelli et al. demonstrated horizontally aligned dense‐bundle CNT interconnects with an impressive wall density of ≈10^13^ cm^−2^ and CNT resistivity is as low as 1.1 mΩ cm, two orders of magnitude above copper (**Figure** **18**a–d).^[^ ^356c^ ^]^ Interconnects with diameters down to 50 nm and lengths up to 20 µm were fabricated by an innovative method of horizontally compacting vertical CNT forests CVD grown from a hole in a silica layer (Figure 18a). This technique also allows for identical end‐bonded contacts to be made on the CNT interconnect. Bottom contacts for vertically aligned CNTs are often unoptimized, as oxidation and interface properties are affected uncontrollably during the CNT synthesis step. Li et al. grew catalyst‐patterned dense vertical CNT forests with >100 µm length, and then used liquid‐assisted densification and flattening^[^ ^188^ ^]^ to produce very long horizontal CNT interconnects with excellent resistivity of 1.7–4.1 mΩ cm.^[^ ^366^ ^]^ The grown CNTs were aligned and individual CNTs covered the >100 µm length of the interconnects, ensuring low resistance along the interconnect. Tawfick et al. similarly grew catalyst‐patterned dense CNT forests, except used mechanical force from a rolling pin to densify and flatten the CNT forests.^[^ ^367^ ^]^ Kim et al. demonstrated a CNT dispersion patterning technique to fabricate aligned SWCNT arrays as CNT interconnects (Section 3.2, Figure 18e).^[^ ^110h^ ^]^ They used a substrate with hydrophilic/hydrophobic regions patterned, dipped the substrate in an aqueous SWCNT dispersion, and slowly withdrew the substrate. SWCNTs were deposited aligned on the hydrophilic regions due to evaporative flow, and then they doped the SWCNTs with Pt to increase conductivity. They consequently created SWCNT interconnect structures (Figure 18f,g) that had current densities up to ≈10^7^ A cm^−2^ (comparable to Cu) demonstrating the ability of CNT dispersion‐based methods to also fabricate competitive interconnects. CNT interconnects can also be fabricated through DEP. Close and Wong used DEP to deposit individual MWCNTs between 250 electrode pairs simultaneously, allowing low‐temperature synthesis of individual CNT interconnects.^[^ ^368^ ^]^ However, fabrication complexity can increase because of the need to construct the electrode pairs on the substrate. Also, DEP‐deposited CNTs are side‐contacted, which has a higher contact resistance than the preferred end‐contact. Liang et al. recently grew single ≈30 nm diameter MWCNTs from < 40 nm via holes fabricated by EBL.^[^ ^369^ ^]^ They then horizontally aligned the single MWCNTs and similar to Kim et al., doped with Pt to convert all MWCNT shells to metallic conductance. This technique realized highly conductive horizontal CNT interconnects from doped individual MWCNTs, demonstrating that nanoscale CNT interconnects can now be fabricated from single CNTs.

### 7.4 Emerging Novel IC Technologies

3D architectures for CNT ICs have been explored to increase power and efficiency of chips.^[^ ^307^, ^370^ ^]^ The 3D integration of multiple layers of computing, memory, and sensing can vastly increase the connectivity between the different layers to more than 1000‐fold. Increased connectivity improves performance of the ICs due to smaller distances, less resistance, less delay, higher bandwidth, and greater energy efficiency. With computing power density and data storage density also an important property for edge computing and IoT, these 3D architectures will be very useful for emerging devices.^[^ ^5^ ^]^ Currently, only microscale patterning techniques are being used to construct these 3D CNT ICs. Nanoscale CNT patterning techniques could increase the densities of these 3D ICs further, and so will be important to consider in the future.

**Figure 19:**
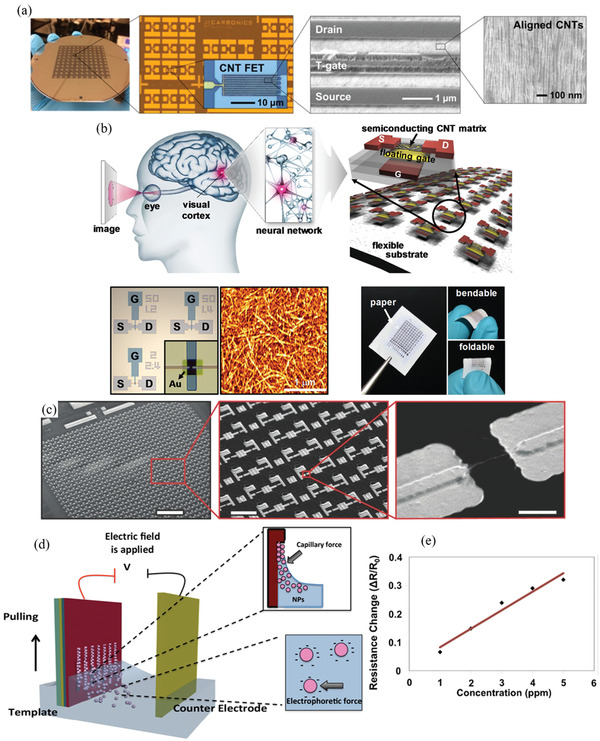
a) Optical and SEM images of synaptic CNTFETs fabricated from patterned aligned SWCNT films for neuromorphic computing. Reproduced with permission.^[^ ^373i^ ^]^ Copyright 2018, American Chemical Society. b) Pattern recognition using synaptic CNTFETs fabricated from patterned random SWCNT networks on flexible substrates. Optical and AFM images of the CNTFETs and random SWCNT networks presented in the bottom left images. Reproduced with permission.^[^ ^373h^ ^]^ Copyright 2017, American Chemical Society. c) SEM image of a nanoscale SWCNT sensor array fabricated via DEP (scale bars left to right 300, 100, and 5 µm). Reproduced with permission.^[^ ^166^ ^]^ Copyright 2017, Wiley‐VCH. d) Electro‐fluidic assembly process used to pattern CNT dispersions on nanoscale templates. Nanoparticles are depicted in the image, but CNTs were patterned in the same manner. e) Plot of resistance change through the CNT‐based NO_2_ gas sensor against the exposed NO_2_ concentration, demonstrating a linear response of the sensor between 1 and 5 ppm. Reproduced with permission.^[^ ^143^ ^]^ Copyright 2017, American Chemical Society.

Neuromorphic computing (brain‐like computing) is a novel computing technology that mimics the function of synapses in biological brains.^[^ ^371^ ^]^ This new computing technology can better incorporate processing and memory functions in the same local device (synapses) compared to conventional von Neumann computers, which require large amounts of data transmission between processing and memory units. This results in a drastically lower energy consumption for neuromorphic computing, and all‐around more efficient computing for complex and unstructured information, including image and spatiotemporal processing, pattern/image/speech recognition, machine learning applications, and more.^[^ ^372^ ^]^ The ability to more efficiently compute complex information with lower power is clearly very beneficial to emerging technologies of edge computing, AI, machine learning, etc. CNTs have been explored as possible material to fabricate the synaptic transistors in neuromorphic computing devices (**Figure** **19**a,b).^[^ ^372^, ^373^ ^]^ Aside from all the beneficial properties CNTs have that are useful for ICs and transistors as explained in Sections 7.1 and 7.2, CNTs also have properties that are particularly useful for neuromorphic computing. Particularly, CNTs can experience charge‐trapping effects, which can produce synaptic behavior by incrementally and controllably altering the channel conductance in a large dynamic range.^[^ ^373h,i^ ^]^ Also, CNTs can form dense, complex, and highly connected structures, increasing the efficiency of the neuromorphic computing devices.^[^ ^373j^ ^]^ These CNT neuromorphic computing devices have been fabricated by a variety of methods including standard oxidation etching patterning from dense CNT arrays/networks (Figure 19a,b)^[^ ^373h,i^ ^]^ and aerosol jet‐printing of CNT inks.^[^ ^373g^ ^]^ However, no method so far has patterned CNTs at nanoscale resolution for neuromorphic computing. Developing new nanoscale CNT patterning techniques and applying them to fabricate CNT neuromorphic devices could vastly increase the efficiency and quality of these devices, while shrinking their size for increased utility.

### 7.5 Sensors

CNTs have a particular advantage for sensor technologies due to their potentially high sensitivity, flexible and robust physical properties, excellent electronic properties, low energy consumption,^[^ ^374^ ^]^ and solution processability. CNTs have already been used to fabricate a variety of sensing including for pressure, strain, bending, thermal effects, many different chemicals present in gas, solute, or liquid form, and biological particles like hormones, antibodies, and others in biological mediums.^[^ ^5^, ^215^, ^375^ ^]^ Sensing CNTs can also be incorporated into monolithic devices that have sensing, processing, and memory components for integrated, independent, and compact devices.^[^ ^216^, ^370^ ^]^ CNTs are consequently a leading material for fabricating future chemical, physical, and biological sensors, including those technologies that will be used for the IoT and medical technologies. However, many of the devices fabricated currently have CNT networks patterned only in micrometer resolution, so are composed of much bigger, visible electric circuits. To decrease resource and energy cost,^[^ ^374^ ^]^ increase power/computing density, increase ubiquitousness, and improve the range of applicability of these devices (even within the human body), the device size will need to decrease. This can be done if the circuits themselves are made smaller with higher‐resolution CNT patterning. Sensors based on individual CNTs also express much higher sensitivities.^[^ ^375^, ^376^ ^]^ It is therefore important to explore possible nanoscale CNT patterning techniques for mass‐production of future sensing devices.

There are some examples listed in **Table** **2** of nanoscale CNT patterning for fabrication of CNT sensors. A common technique is DEP, which can produce experimental nanoscale or individual CNT devices easily and effectively. Seichepine et al. assembled a CMOS microsystem from 1024 CNT sensors using AC DEP.^[^ ^166^ ^]^ A single deposition step was required; placing an aqueous CNT/carboxymethyl cellulose suspension between fixed and floating Pt electrode pairs and applying a 20 V, 300 kHz AC bias for 60 min. Their deposition yielded 80% of 5 µm gap, 100 µm period electrode pairs bridged with one to five CNTs (Figure 19c). As found in previous studies, increased deposition time and increased voltage resulted in more CNTs deposited bridging the electrodes, but not necessarily distributed evenly between electrodes. The 32 × 32 array of CNT devices was used to detect pH changes in solutions and allowed constant monitoring over multiple cycles. The sensors increased in resistance when placed in lower pH solution, and vice versa. CNT sensors have also been made through other nanoscale patterning techniques like template patterning of CNTs from dispersions. Yilmaz et al. demonstrated a NO_2_ gas sensor made from a 300 nm wide CNT channels printed using a nanoscale electro‐fluidic template CNT dispersion patterning technique, followed by transfer onto device substrate (Figure 19d).^[^ ^143^ ^]^ NO_2_ could easily be detected from ambient air down to 1 ppm (Figure 19e). The patterning technique is very quick (<1 min deposition) and can be transferred onto flexible substrates, potentially allowing flexible next‐generation CNT sensors to be manufactured with high throughput and low cost.

**Table 2:** Sensors fabricated using nanoscale CNT patterning techniques

Author	Target analyte	Nanoscale patterning technique	Sensitivity and response	Notes
Suehiro et al.^[^ ^377^ ^]^	NH_3_ gas	DEP	Linear response 0–10 ppm, few minutes response time	Ammonia reduces p‐type MWCNTs, reducing conductance
Chung et al.^[^ ^378^ ^]^	O_2_ gas	DEP	–	Electrical breakdown of the MWCNTs was required to attain O_2_ sensitivity
Suehiro et al.^[^ ^379^ ^]^	H_2_ gas	DEP	0.05% H_2_ at 90 °C	Pd electrodes and/or functionalization required for H_2_ response
Liu et al.^[^ ^380^ ^]^	Nitro‐phenol in aqueous	DEP	Linear response 0.04 × 10^−3^–0.28 × 10^−3^ m, minimum 0.016 × 10^−3^ m, seconds response time	Reusable
Peng et al.^[^ ^381^ ^]^	NH_3_ gas	DEP	178% conductance change per ppm at 20 °C, 32.8 s response time	Ammonia on + bias CNTFETs increase in Schottky barrier height at the Au‐CNT interface. Electrical breakdown of metallic CNTs was required
Chen et al.^[^ ^382^ ^]^	Thermal sensor	DEP	Measured TCR −0.43% °C^−1^	Integrated into CMOS circuitry
Burg et al.^[^ ^383^ ^]^	Strain sensor	DEP	≈0.25 Δ*R* R^−1^ bar^−1^	Low power consumption <40 nW, individual SWCNTs placed between electrodes
Ganzhorn et al.^[^ ^384^ ^]^	H_2_ gas	DEP	100‐fold conductance change at 100 ppm, current change of a decade in >10 s	Hydrogen affects the work function of Pd, altering the Schottky barrier between the Pd electrode and the semiconducting CNT
Yilmaz et al.^[^ ^143^ ^]^	NO_2_ gas	Electro‐fluidic template patterning	1 ppm minimum	300 nm wide CNT channel
Seichepine et al.^[^ ^166^ ^]^	pH	DEP	2.4 nA per pH	1024 CNT devices incorporated in a CMOS system. 80% yield of 1–5 CNTs

### 7.6 Photonic and Optical Applications

**Figure 20:**
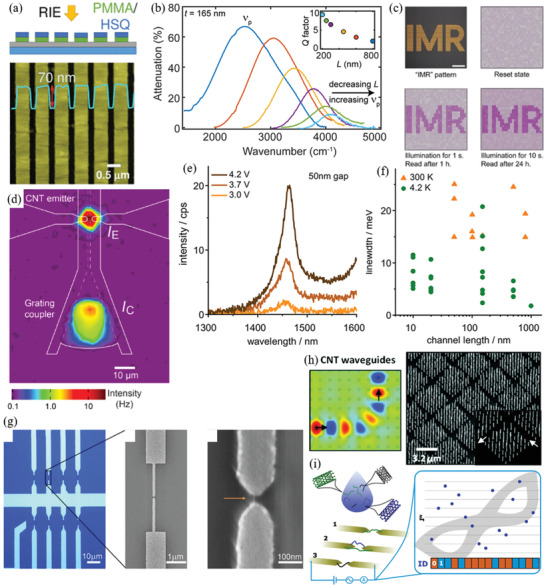
a) Illustration and AFM image of highly aligned SWCNT film cut into nanoscale stripes using oxidation etching through an EBL patterned resist. b) Attenuation due to coherent plasmon resonance in nanoscale patterned SWCNT films. Decreasing SWCNT lengths from 800 to 200 nm results in increasing peak wavenumber. Reproduced with permission.^[^ ^216i^ ^]^ Copyright 2017, American Chemical Society. c) Flexible CNT device incorporating both photodetection and memory applications fabricated by oxidation etching. The device can store sensed images in memory for up to 24 h after only 10 s exposure. Reproduced with permission.^[^ ^216k^ ^]^ Copyright 2020, Wiley‐VCH. d) Spatially resolved light emission of a CNT transducer device fabricated by DEP CNT patterning. Strong and stable emission is observed from the SWCNTs bridging the electrodes coupled with a waveguide. Reproduced under the terms of CC BY 4.0 license.^[^ ^395a^ ^]^ Copyright 2017, The Authors, published by Beilstein‐Institut. e) Typical electroluminescence spectra of individual (9,8) SWCNT devices with short channels at room temperature. 50 nm channel (gap) is presented at varying bias voltages. f) Peak full‐width at half‐maximum for varying channel lengths at room and cryogenic temperatures. g) Optical (left) and SEM image (middle) of the devices and SEM image (right) of a (9,8) SWCNT deposited via DEP between electrodes in the device. Reproduced with permission.^[^ ^396^ ^]^ Copyright 2020, American Chemical Society. h) Vertical CNT array waveguide metamaterial fabricated by EBL patterning of CNT growth catalyst and subsequent CVD growth. Reproduced with permission.^[^ ^398^ ^]^ Copyright 2011, American Chemical Society. i) Heterogenous mixture of CNTs can be patterned by DEP CNT patterning onto a device to produce randomly generated resistances for each device. These resistances can be read as a physically unclonable function for cryptographic identification. Reproduced with permission.^[^ ^401b^ ^]^ Copyright 2019, American Chemical Society.

Semiconducting SWCNTs express strong photoluminescence and electroluminescence^[^ ^341^ ^]^ and their emission wavelength can be tuned through 850 nm to 2 µm simply by selecting specific chiralities.^[^ ^385^ ^]^ This makes SWCNTs very useful for many photonic applications.^[^ ^16^, ^385^ ^]^ SWCNTs can be used for nanoscale photonic devices including: efficient light‐emitting diodes,^[^ ^386^ ^]^ electrically driven bright thermal light emitters,^[^ ^387^ ^]^ and room‐temperature photodetectors.^[^ ^388^ ^]^ Nanoscale CNT patterning techniques can also be used as optical CNT devices like terahertz/infrared antennas^[^ ^216i^ ^]^ or SERS‐active substrates.^[^ ^389^ ^]^ Chiu et al. used nanoscale patterning of pre‐aligned SWCNT films with oxidation etching to create uniform length SWCNTs that promote coherent plasmon resonances (**Figure** **20**a,b).^[^ ^216i^ ^]^ These patterned films have vastly increased absorption due to the plasmon resonances, allowing them to be used as high‐quality terahertz and IR antennas. Controlling the uniform length of these SWCNTs tunes the wavelength from terahertz to near‐IR (1.4–200 µm) (Figure 20b).^[^ ^216^, ^390^ ^]^ This feature emphasizes the importance of precise patterning during manufacturing to obtain desired device properties. Individualized metallic SWCNTs have also recently been formed as ultraclean nanocavities that can be used as nanoscale low‐loss Fabry–Perot plasmonic resonators.^[^ ^391^ ^]^ These plasmonic resonators can be used as components in future nanophotonic circuitry.

Preliminary fully integrated optoelectronic devices have also recently been fabricated using CNT patterning, albeit with microscale resolution. Liu et al. demonstrated a monolithic 3D optoelectronic IC based on patterned CNTs which included CNT photovoltaic receivers, CNT electrically driven transmitters, and electronic circuits in multiple layers.^[^ ^216j^ ^]^ Using the excellent optoelectronic properties of CNTs, they were able to monolithically incorporate connections of memory and computing through parallel and rapid optical communication between stacked 3D functional layers. Qu et al. similarly incorporated photodetection and memory applications into a single monolithic flexible device fabricated by photolithography and oxidation etching of CNTs.^[^ ^216k^ ^]^ The device could detect light and store the data for up to 24 h using the same flexible IC with only 10 s exposure time (Figure 20c).

SWCNTs also have potential as quantum light sources with the ability to become excellent single‐photon sources at room temperature with tunability to telecom wavelengths (1.3–1.55 µm).^[^ ^385^, ^392^ ^]^ These quantum light sources are used for emerging technologies such as quantum computing, photonic computing, and quantum cryptography. However, individualized SWCNTs are required to be accurately placed in particular locations in order to study their quantum optics/emissions and use the quantum light source in devices. SWCNTs can also suffer from low brightness/low quantum yields when used in isolation. Coupling SWCNTs with microcavities by patterning individualized SWCNTs over microcavities can improve emission brightness/intensity by one to two orders of magnitude while narrowing the emission spectrum.^[^ ^385^, ^392^, ^393^ ^]^ Cavity‐coupling has allowed SWCNT device emission quantum yields to reach ≈62%, emission rate to 15 MHz, and line widths down to 18 μeV.^[^ ^393e^ ^]^ Nanoscale CNT patterning techniques are therefore critical to realize high‐quality CNT quantum light devices. DEP has proven to be an extremely useful and scalable technique in placing individualized SWCNTs as quantum light sources for nanophotonic circuits.^[^ ^393^, ^394^ ^]^ Recently, Khasminskaya et al. fabricated a photonic circuit using DEP to pattern individual SWCNTs between electrodes over waveguides.^[^ ^393c^ ^]^ The SWCNTs could then be biased to emit photons into the waveguides which were terminated with nanowire single‐photon detectors. The group demonstrated further examples of waveguide‐coupled SWCNT devices assembled through DEP patterning achieving light pulse generation up to 2 GHz and <80 ps decay times (Figure 20d).^[^ ^395^ ^]^ Single SWCNTs can also be grown directly over microcavities from patterned CNT growth catalyst.^[^ ^393a^ ^]^ Single SWCNT devices with ultrashort channels of 10 nm were recently assembled via DEP and demonstrated stable emission at telecom wavelengths (Figure 20e–g),^[^ ^396^ ^]^ showing the potential of SWCNT photonic devices to be implemented in ULSI nanoscale photonic circuitry. Advanced nanoscale CNT patterning techniques will help to realize commercial manufacturing of these CNT nanophotonic devices.

Periodic arrays of vertically aligned metallic CNTs can be easily created with periodically patterned catalyst and subsequent PECVD. Metallic CNTs can be synthesized this way with very high aspect ratios. This easy fabrication of 2D, subwavelength periodic, high‐aspect ratio, metallic structures makes PECVD‐grown CNTs an excellent candidate for photonic metamaterials. The ability to change the dimensions of the CNT diameters and spacing allows CNT arrays to be tailored to interact with different EM frequency ranges. Periodic CNT arrays can operate as metamaterial high pass frequency filters.^[^ ^397^ ^]^ CNT arrays have plasma frequencies much lower than metal and so can be utilized for filtering microwave and terahertz frequencies. Exact diameters and spacings are needed for these arrays, as filter cut‐off frequencies are dependent on CNT array geometry. Butt et al. calculated and experimentally realized near‐IR cut‐off frequency for periodic MWCNT arrays with 50 nm diameter and 400 nm spacings.^[^ ^397^ ^]^ They used EBL to pattern 100 nm diameter periodic Ni catalyst dots, for which they used PECVD to grow 1–2 µm tall MWCNTs with 50 nm diameter. These CNT array metamaterials can also be operated as wave guides when patterned with wider gaps as the guides (Figure 20h),^[^ ^398^ ^]^ or as iridescent biconvex microcavities with high *Q* factors.^[^ ^399^ ^]^

### 7.7 Other Applications

Vertically aligned CNT arrays can operate as field electron emitters for a variety of applications, including electron gun sources for electron microscopes and electron lithography. The field enhancement factor, a figure of merit for field electron emitters, is quite high for vertically aligned CNT arrays due to their high aspect ratios, prompting significant research interest.^[^ ^228^, ^237^, ^241^, ^243^, ^400^ ^]^ It is important to obtain individual and spaced CNTs for use in field emission devices. This is to ensure high quality and precise emission without interference between adjacent emitters. Teo et al. found that to achieve a high yield of individualized CNTs during the PECVD process, patterned catalyst domains of <300 nm diameter are required, with <100 nm required for ≈100% yield.^[^ ^272^ ^]^ For catalyst domains with >300 nm diameter, average number of CNTs grown from the domains is approximately proportional to domain size.

Intrinsically heterogenic mixtures of SWCNTs with different chirality and impurities can be useful for creating more cryptographically secure identification systems.^[^ ^401^ ^]^ Heterogenic SWCNTs patterned by DEP or substrate modification patterning can create unique and random electrical properties for each element of a device array (Figure 20i). This randomized array is used as a physically unclonable function, where the unique conduction of each element produces an unreproducible code that can be used for cryptographic identification. This is important to increase the security of digital systems and electronic devices as the evolving IoT produces many more devices that require unhackable security.

Microelectromechanical devices using dense CNT forests have been fabricated using water‐assisted densification and oxidation etching (Figure 10g–j).^[^ ^188^, ^216^ ^]^ Patterns of vertically aligned CNT forests can be used as stamps for precise and high aspect ratio NIL.^[^ ^402^ ^]^ In a similar way, the patterned vertically aligned CNT forests can effectively hold ink and be used for high‐resolution flexographic patterning.^[^ ^403^ ^]^ Nanoscale CNT electrodes can be fabricated by using AC DEP to bridge a CNT between larger electrodes, and then cutting the CNT with EB oxidation.^[^ ^404^ ^]^ This provides an easy method to fabricate nanoscale electrodes with <20 nm gaps. Ye et al. demonstrated a large‐scale method of fabricating CNT probe tips for AFM, by employing EBL and PECVD in a refined process.^[^ ^405^ ^]^ They deposited CNT growth catalyst through an EBL patterned resist onto silicon‐on‐insulator substrates, and they employed further standard photolithographic and etching steps to reveal a fabricated probe with catalyst patterned on the end of the cantilever. They then used PECVD under optimized conditions to produce singular, vertically aligned CNTs with 40–80 nm diameters and 2–6 µm lengths at the catalyst sites. Normally EBL is too slow for mass production, but they reasoned it was viable because they only patterned a small area, taking ≈20 min per wafer. The fabricated CNT probes were able to measure down to 200 nm in 90 nm wide trenches, showing the ability of CNT tips.

## 8 Summary and Future Directions

The past few decades have seen many different nanoscale resolution CNT patterning techniques developed and explored, each with particular advantages and disadvantages. Some techniques have been developed that have allowed excellent control of individual CNT placement with nanoscale precision. Other techniques developed have attained submicron lateral resolution while simultaneously achieving relatively high throughput and potential R2R compatibility.

High‐performance CNT devices have mostly been fabricated using nanoscale oxidation etching through resists with considerable success. The main advantage is the ease of incorporating this approach into standard CMOS manufacturing processes while obtaining high resolution and being able to use chirally pure and aligned SWCNTs. There are still limitations in resolution and manufacturability though, and the technique is not high throughput. Modeling showed that highly aligned SWCNTs placed with controlled spacing of 5–10 nm on device substrates could maximize the performance of CNTFETs and allow them to supersede current state‐of‐the‐art FETs. Further research into CNT patterning techniques that can achieve this requirement should be investigated to realize these advanced FET technologies. Potential investigations could include using directed self‐assembly (DSA) of block copolymers to create nanotemplates with 5–10 nm pattern period to guide aligned, controlled spacing SWCNT deposition, possibly incorporating (di)electrophoresis to increase deposition speed and precision.^[^ ^406^ ^]^ Spacing between surfactant‐dispersed SWCNTs deposited on SAM patterned substrates may also potentially be controlled by controlling the surfactant length. The surfactant length can determine the effective “shielding effect” radius, which is the excluded area SWCNTs will not deposit near an already deposited SWCNT.^[^ ^124^ ^]^ DNA linkers could be explored to obtain precise spacing between deposited aligned SWCNTs through DNA segments with defined lengths.^[^ ^407^ ^]^

Patterning CNTs from dispersions using modified substrate or templates has shown great promise recently to achieve both high resolution and high throughput. Sub‐micron resolution has been demonstrated in R2R compatible techniques, with no apparent limit yet to the simultaneous improvement of resolution and throughput. The self‐alignment effect at nanoscale dimensions also increases the potential utility (Section 2.4.3). To realize the potential of CNT dispersion patterning techniques, further research and investigation should be conducted into bypassing some of the trade‐offs inherent in CNT dispersion pattering (Section 2.1.6). Addressing the underlying physical and chemical aspects that cause the current limits is crucial for further advances. Research should be conducted to determine methods of obtaining dispersions of high‐quality CNTs at much higher concentration without impacting the rheological properties, possibly through exploration of novel functionalizations, dispersing agents, and even dispersing mediums. Better CNT dispersions will enable faster and more precise deposition of higher‐quality CNTs onto substrates. Methods of increasing the velocity of dispersed CNTs through the dispersing medium should be explored for increasing throughput, including by adding elements like (di)electrophoresis, magnetophoresis, or even diffusiophoresis to current techniques. Methods of increasing the flow of CNT dispersions while maintaining nanoscale resolution will also help to increase throughput. Nanoscale patterned templates, filters, or even micro/nanofluidics have fixed geometries that will help maintain nanoscale resolution for patterning while increasing the CNT dispersion flow velocity for higher throughput. However controlling flow of colloidal dispersions through confined micro/nanoscale geometries presents more challenges that should be researched further. DEP patterning has also shown grown potential allowing whole wafer nanoscale resolution patterning of individual CNTs. DEP patterning's main limitation is the electrodes, although the recent demonstration of removable graphene electrodes has helped to alleviate this issue. DEP could also be improved by increased throughput. Perhaps DEP could be configured in a way to allow R2R compatibility to increase throughput, requiring novel physical/chemical techniques or innovative engineering. Many of these techniques could also benefit from incorporating a sophisticated transfer process from the patterning substrate onto the device substrate. In this way, the requirements of the patterning substrate (SAM features, patterned resist, DEP electrodes, etc.) can be decoupled from the device substrate, removing limitations and requirements on device substrates and possibly allowing for a R2R process.

Pre‐synthesis patterning has been implemented in a wide variety of ways, but they all are restricted by the subsequent high temperature required for CNT growth. The subsequently patterned CNTs can be extremely straight, especially when using crystalline substrates for directing growth. This is important for low device‐to‐device variability during manufacturing. These patterning techniques could be greatly advanced by further research into colder CNT synthesis methods that remove the current high‐temperature limitation.

The development of CNT patterning has recently been driven mainly by the requirements of the intended applications. Generally, if the patterning technique is sufficient to fabricate a workable device, then it is implemented without much modification. However, if CNT patterning techniques were further developed without prompt from current application requirements, new applications and advanced devices may be unlocked that were not previously considered. There is still much that can be improved in the field, as large‐scale, high throughput, nanometer resolution patterning of individualized CNTs has not been achieved. Advanced techniques could also at least improve the commercial prospects of advanced CNT devices as manufacturability or device performance increases. CNTs have not been employed in commercial manufacturing as much as expected yet due to challenges with synthesizing CNTs with consistent and high‐quality properties as well as patterning of CNTs in devices. Improving CNT patterning techniques in terms of quality and manufacturability may vastly increase the commercial viability of CNT devices and initiate the era of CNT devices. In the future with continued research, we may see high‐performance CNT devices and even computers that can be patterned cheaply and quickly on flexible substrates for future ubiquitous sensing, communication, memory, and computing.

## Conflict of Interest

The authors declare no conflict of interest.
